# Dentifragilones A–B and Other Benzoic Acid Derivatives from the European Basidiomycete *Dentipellis fragilis*

**DOI:** 10.3390/molecules29122859

**Published:** 2024-06-16

**Authors:** Winnie Chemutai Sum, Sherif S. Ebada, Mahmoud A. A. Ibrahim, Harald Kellner, Marc Stadler

**Affiliations:** 1 Department of Microbial Drugs, Helmholtz Centre for Infection Research (HZI) and German Centre for Infection Research (DZIF), DZIF Partner Site Hannover-Braunschweig, Inhoffenstrasse 7, 38124 Braunschweig, Germany; winnie.sumchemutai@helmholtz-hzi.de (W.C.S.); or sherif_elsayed@pharma.asu.edu.eg (S.S.E.); 2 Institute of Microbiology, Technische Universität Braunschweig, Spielmannstraße 7, 38106 Braunschweig, Germany; 3 Department of Pharmacognosy, Faculty of Pharmacy, Ain Shams University, Cairo 11566, Egypt; 4 Computational Chemistry Laboratory, Chemistry Department, Faculty of Science, Minia University, Minia 61519, Egypt; m.ibrahim@compchem.net; 5 School of Health Sciences, University of KwaZulu-Natal, Westville, Durban 4000, South Africa; 6 Department of Bio- and Environmental Sciences, Technische Universität Dresden-International Institute Zittau, Markt 23, 02763 Zittau, Germany; harald.kellner@tu-dresden.de

**Keywords:** Basidiomycota, Hericiaceae, crassinervic acid

## Abstract

A chemical and biological exploration of the European polypore *Dentipellis fragilis* afforded two previously undescribed natural products (**1** and **2**), together with three known derivatives (**3**–**5**). Chemical structures of the isolated compounds were confirmed through 1D/2D NMR spectroscopic analyses, mass spectrometry, and by comparison with the reported literature. The relative and absolute configurations of **1** were determined according to the ROESY spectrum and time-dependent density functional theory electronic circular dichroism (TDDFT-ECD), respectively. Furthermore, the absolute configuration of dentipellinol (**3**) was revisited and revealed to be of (*R*) configuration. All the isolated compounds were assessed for their cytotoxic and antimicrobial activities, with some being revealed to have weak to moderate antimicrobial activity, particularly against Gram-positive bacteria.

## 1. Introduction

Fungal-based natural products have made an immense contribution to the modern-day medical and agrochemistry sectors, with their vast chemical novelties unmatched by many sources. Over the last two centuries, regions rich in biodiversity have undoubtedly proved to be invaluable sources of therapeutic targets useful for the global pharmaceutical industries [1]. Nonetheless, Basidiomycota of the temperate zones, assumed to be well sampled, still offer great opportunities for novelty [2]. The rare Basidiomycota of Europe in particular have been neglected, but constitute potential reservoirs of new pharmacotherapeutic agents [3].

During our investigations of the seldom-found Basidiomycetes of Germany, we encountered *Dentipellis fragilis*, a red-listed member of the wood-rot fungi of the Hericiaceae family. The fungal genus *Dentipellis* was coined by Anton Donk in 1962 and, thus far, *D. fragilis* is the only studied species of the genus with regards to its secondary metabolites. It is worth mentioning that *D. fragilis* has been demonstrated to be quite ‘talented’ in relation to the diversity of its produced natural compounds, with bioactive phthalide, cyathane, benzofuranone, and drimane derivatives having recently been reported from the fungus [4,5,6,7,8,9]. In particular, the discovery of cyathanes is in strong accordance with the placement of *Dentipellis* in the Hericiaceae family, since the same compound class is also characteristic of cultures of other genera in this family like *Hericium* and *Laxitextum* [2,6]. The current paper is dedicated to describing additional secondary metabolites (SMs) of the strain studied by Sum et al. [6] that were obtained upon modifying the culture conditions.

## 2. Results and Discussion

### 2.1. Chemical Characterization of **1**–**5** (Figure 1)

Compound **1** was isolated as a white amorphous solid. The HR-ESI-MS of **1** revealed a protonated molecular ion and sodium adduct peaks at *m/z* 227.1277 [M+H]^+^ (calculated 227.1278) and 249.1098 [M+Na]^+^ (calculated 249.1097), respectively, determining its molecular formula as C_12_H_18_O_4_ and hence indicating four degrees of unsaturation. The ^13^C NMR and HSQC spectral data of **1** (Table 1, Figures S4 and S7) unveiled the presence of twelve carbon resonances that can be classified into five unprotonated carbon atoms separated into one carbonyl carbon at δ_C_ 195.8 (C-1), two olefinic carbon atoms at δ_C_ 144.0 (C-2), 130.0 (C-3), and two sp^3^ carbon atoms at δ_C_ 44.8 (C-7) and 80.4 (C-6). In addition, the ^13^C NMR spectral data revealed one methylene sp^3^ carbon atom at δ_C_ 36.7 (C-4) and one methylidene sp^2^ carbon atom at δ_C_ 113.2 (C-9). The 1D (^1^H and ^13^C) NMR data and HSQC spectrum of **1** (Table 1, Figure S7) revealed the presence of three olefinic protons at δ_H_ 6.04 (dd, *J* = 17.4, 10.7 Hz, H-8; δ_C_ 143.6), δ_H_ 5.02 (d, *J* = 10.7 Hz, H-9α), and δ_H_ 5.09 (d, *J* = 17.4 Hz, H-9β) that were both correlated to sp^2^ methylene carbon at δ_C_ 113.2 (C-9). The ^1^H–^1^H COSY spectrum of **1** (Figure 2 and Figure S5) revealed the presence of one spin system between three olefinic protons ascribed to H-8 and H_2_-9, indicating their presence as an allyl moiety. The ^1^H–^1^H COSY spectrum of **1** (Figure 2 and Figure S5) also revealed an additional spin system from H-5 (δ_H_ 4.18, ddd, *J* = 9.4, 5.8, 2.8 Hz) to two diastereotopic methylene protons at δ_H_ 2.56/2.74 (H_2_-4). The HMBC spectrum of **1** (Figure 2 and Figure S6) revealed the presence of two diastereotopic singlet methyl groups at δ_H_ 1.07 (H_3_-11; δ_C_ 24.4) and 1.26 (H_3_-12; δ_C_ 23.9) that revealed correlations with three different carbon atoms assigned as C-6, and C-7 and two allylic carbon atoms, C-8/C-9. Furthermore, the HMBC spectrum of **1** (Figure 2) revealed key correlations between H_3_-10 and two unprotonated sp^2^ carbons C-2/C-3, together with a long-range “ω” correlation with the carbonyl carbon (C-1), indicating their existence as an α,β-unsaturated carbonyl moiety in **1**.

**Figure 1:**
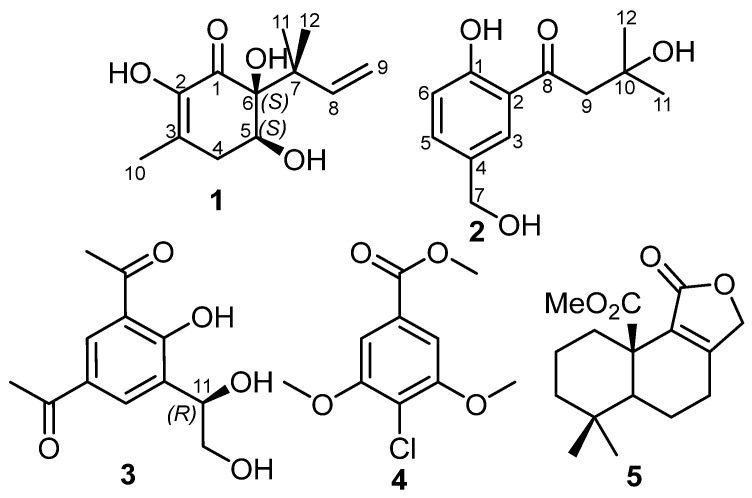
Chemical structures of **1**–**5**.

**Figure 2:**
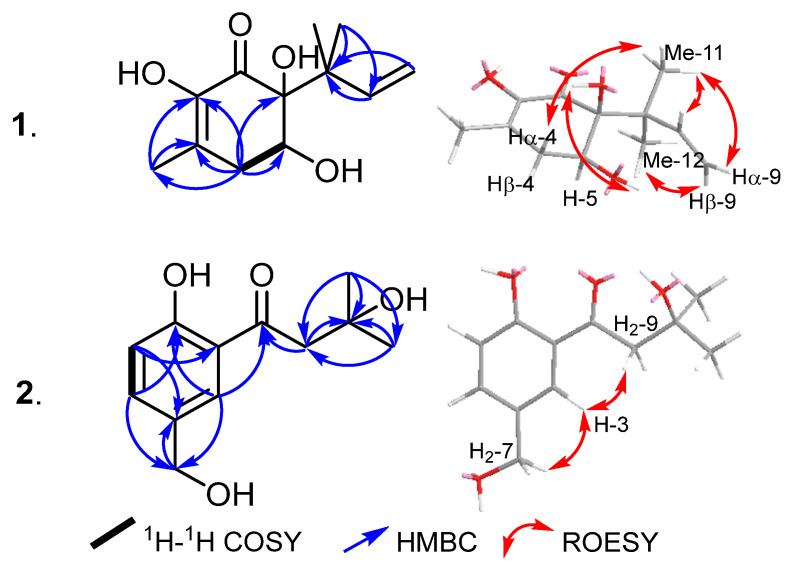
Key ^1^H–^1^H COSY, HMBC, and ROESY correlations of **1** and **2**.

**Figure 3:**
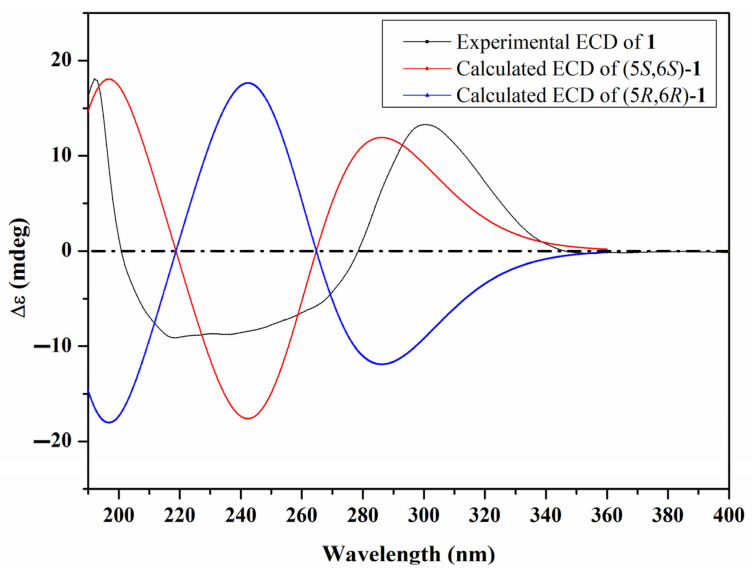
Experimental and calculated ECD spectra of **1**.

A literature search of **1** revealed its common structural features of strobiloscyphones [10], pestallic acids [11], dentipellin [4], and the recently reported lachnoic acids [12], where they shared the presence of a 2-cyclohexenone moiety in their structures. A careful interpretation of the obtained 1D and 2D NMR spectral data of **1** (Table 1, Figure 2) suggested the structure depicted in Figure 1, with a 1,1-dimethyl-2-propenyl functionality being attached at C-6. The relative configuration of **1** at C-5 and C-6 was determined by its ROESY spectrum, which revealed an ROE correlation between two exchangeable broad singlet proton signals at δ_H_ 3.44 and 2.37 assigned to 5-OH and 6-OH, respectively. This thus indicated their cofacial orientation, while the 1,1-dimethyl-2-propenyl moiety is projected toward the opposite face of the molecule. The ROESY spectrum of **1** (Figure 2 and Figure S8) also revealed key ROE correlations between the two diastereotopic methyl groups, H_3_-11 and H_3_-12, and H-4β, H-5, H-8, and H_2_-9, confirming the depicted structure of **1**. Accordingly, the ROESY spectrum suggested the relative configurations at C-5 and C-6 were either (5S*,6S*) or (5R*,6R*). To determine the absolute configuration of **1**, its ECD spectrum was acquired and, hence, compared to the calculated TDDFT-ECD spectra of (5*S*,6*S*) and (5*R*,6*R*) enantiomers. The obtained results (Figure 3) revealed a coherence between both the experimental and calculated ECD spectra of (5*S*,6*S*) configuration. Based on the obtained results, compound **1** was identified as a previously undescribed natural product named dentifragilone A.

Compound **2** was purified as an off-white amorphous solid. The HR-ESI-MS revealed a protonated molecular ion peak and a sodium adduct peak at *m/z* 225.1117 [M+H]^+^ (calculated 225.1121) and 247.0937 [M+Na]^+^ (calculated 247.0941), respectively, determining its molecular formula as C_12_H_16_O_4_ and indicating five degrees of unsaturation. The ^1^H NMR spectral data and the ^1^H–^1^H COSY spectrum of **2** (Table 2, Figure S12) revealed three aromatic proton signals at δ_H_ 7.75 (d, *J* = 2.1 Hz), 7.49 (dd, *J* = 8.5, 2.1 Hz), and 7.00 (d, *J* = 8.5 Hz) that were correlated as one spin system, suggesting their presence on a 1,2,4-trisubstituted aromatic ring. In addition, the ^1^H NMR spectral data of **2** (Table 2) revealed the presence of three singlet proton resonances that were categorized, according to their integration indices, into two methylene groups at δ_H_ 4.66 and δ_H_ 3.20 (each of an integration index of two), together with two magnetically equivalent methyl groups at δ_H_ 1.37 integrated for six protons. The ^13^C NMR spectral data and DEPTQ spectrum (Table 2, Figure S16) revealed the presence of five unprotonated carbon signals resolved into one carbonyl carbon at δ_C_ 207.2, three sp^2^ carbon atoms (δ_C_ 162.3, 131.4, and 119.2), and one oxygenated sp^3^ carbon (δ_C_ 70.0). A literature search for **2** revealed its close resemblance to methyl 4-hydroxy-3-(3-methylbutanoyl) benzoate, a fungal metabolite that has been previously reported from scrap cultivation beds of *Hericium erinaceus* (published by Ueda et al. [13] under the grammatically incorrect name “*H. erinaceum*”).

Further characterization for the suggested structure of **2** was obtained via the HMBC spectrum (Figure 2 and Figure S13), which revealed key correlations between two aromatic protons assigned to H-3 (δ_H_ 7.75) and H-5 (δ_H_ 7.49), the hydoxymethylene carbon at δ_C_ 64.2 (C-7), and an oxygenated aromatic carbon at δ_C_ 162.3 (C-1), indicating that the hydroxymethylene group is bound at C-4. The HMBC spectrum of **2** also revealed key correlations from two magnetically equivalent methyl groups (H_3_-11/H_3_-12) to C-10 (δ_C_ 70.0) and C-9 (δ_C_ 48.1), whereas the methylene group at δ_H_ 3.20 (H_2_-9) and H-3 revealed correlations to a carbonyl carbon (C-8), indicating the presence of a 3-hydroxy-3-methylbutanoyl moiety at C-2 on the aromatic ring. The ROESY spectrum of **2** (Figure 2) revealed key correlations from H-3 to both H_2_-7 and H_2_-9, confirming the depicted arrangement of substituents on the aromatic ring at C-1, C-2, and C-4. A literature search revealed that compound **2** revealed a reduced primary alcohol derivative related to crassinervic acid, an antifungal metabolite from *Piper crassinervium* [14]. Based on the obtained results, compound **2** was identified as a previously undescribed natural product and it was given a trivial name, dentifragilone B.

Compound **3** was obtained as an off-white amorphous solid. Its molecular formula was established to be C_12_H_14_O_5_, indicating six degrees of unsaturation. A literature search of **3**, based on its molecular formula and the 1D (1H and ^13^C) NMR spectral data (Table S1), revealed that the measured values were in close accordance with those recently reported for dentipellinol [5]. Although the absolute configuration of dentipellinol (**3**) was reported to be (*S*) configuration [5], herein its structure was revisited and determined by comparing its experimental and calculated ECD spectra (Figure S4). The results in Figure S4 indicate a closer coherence to the calculated ECD spectrum of (*R*) configuration than that of (*S*). Moreover, the 3D coordinates provided in the Supplementary Material of Ki et al.’s study confirmed the (*R*) configuration of dentipellinol (**3**), which was misinterpreted as (*S*) [5].

In addition, compounds **4** and **5** were identified as methyl 4-chloro-3,5-dimethoxybenzoate and 10-methoxycarbonyl-10-norisodrimenin, respectively, based on their HR-ESI-MS data and 1D/2D NMR spectral analyses compared to the reported literature [14,15].

### 2.2. Biological Assays

To assess the antimicrobial activity of compounds **1**–**4**, a serial dilution assay was conducted against several Gram-positive and Gram-negative bacteria as well as fungal strains. Compound **5** was not tested, since similar activities had been reported by our group in a recent study [15]. Notably, compound **4** demonstrated moderate or weak antibiotic effects against *Staphylococcus aureus* with a MIC value of 66.6 µg/mL. Compounds **1**–**3** were inactive in the antimicrobial tests.

An evaluation of the cytotoxic activities of the isolated compounds was first conducted against the two most sensitive cell lines, namely mouse fibroblast (L929) and human endocervical adenocarcinoma (KB3.1). The compounds had no apparent cytotoxic effects; hence, further tests were not conducted.

## 3. Materials and Methods

### 3.1. General Experimental Procedures

Optical rotations (OR) were recorded on a MCP-150 polarimeter (Anton Paar; Seelze, Germany) at 20 °C using methanol (Uvasol, Merck; Darmstadt, Germany). UV-Vis spectra measurements were acquired using a UV-Vis spectrophotometer UV-2450 (Shimadzu; Kyoto, Japan), while electronic circular dichroism (ECD) spectra were measured using a J-815 spectropolarimeter (Jasco, Pfungstadt, Germany).

Nuclear magnetic resonance (NMR) spectra were recorded using an Avance III 500 MHz spectrometer equipped with a BBFO (plus) SmartProbe (^1^H: 500 MHz, ^13^C: 125 MHz; Bruker, Billerica, MA, USA) and an Avance III 700 MHz spectrometer equipped with a 5 mm TCI cryoprobe (^1^H: 700 MHz, ^13^C: 175 MHz; Bruker, Billerica, MA, USA) (sample temperature: 298 K). The NMR data were referenced to selected chemical shifts δ of chloroform-*d* (^1^H, δ = 7.27 ppm; ^13^C, δ = 77.2 ppm) and methanol-*d*_4_ (^1^H, δ = 3.31 ppm; ^13^C, δ = 49.0 ppm). Electrospray ionization mass (ESI-MS) spectra were acquired with an UltiMate^®^ 3000 Series uHPLC (Thermo Fisher Scientific; Waltman, MA, USA) employing a C_18_ Acquity^®^ UPLC BEH column (50 × 2.1 mm, 1.7 μm; Waters, Milford, MA, USA) (temperature of the column: 40 °C) and connected to an amaZon^®^ speed ESI-Iontrap-MS (Bruker; Billerica, MA, USA). The following parameters were used to set up the HPLC system: solvent A: Deionized H_2_O + 0.1% formic acid (FA) (*v*/*v*); solvent B: acetonitrile (MeCN) + 0.1% FA (*v*/*v*) as the mobile phase; gradient: 5% B for 0.5 min increasing to 100% B in 19.5 min and maintaining isocratic conditions at 100% B for 5 min; flow rate: 0.6 mL/min; and Diode-Array Detection (DAD) at 190–600 nm. The crude extracts and pure compounds were dissolved in a solution of acetone and methanol (1:1) to achieve concentrations of 4.5 mg/mL and 1.0 mg/mL, respectively. High-resolution electrospray ionization mass spectrometry (HR-ESI-MS) spectra were measured through an Agilent 1200 Infinity Series HPLC–UV system (Agilent Technologies, Böblingen, Germany) with the same conditions as for ESI-MS spectra, connected to a maXis^®^ ESI-TOF mass spectrometer (Bruker; Daltonics, Bremen, Germany)) (scan range 100–2500 *m/z*, capillary voltage 4500 V, dry temperature 200 °C).

### 3.2. Fungal Material

The fungus was collected on a decaying beech (*Fagus sylvatica*) in the Bavarian Forest National Park (49.098387 N, 13.246003 E) in August 2015 [16] and cultured by one of the authors (HK). The mycelial culture was deposited at the Deutsche Sammlung von Mikroorganismen und Zellkulturen (DSMZ), Braunschweig, designated as DSM 105465. Its identification was reported in our previous study [15] and an ITS-nRDNA sequence of the strain is deposited at the GenBank under the accession number MK463979. We would, however, like to point out that ITS sequences are unreliable for fungal identification and the morphological characters of the specimen already allowed for an unambiguous assignment to the taxon *Dentipellis fragili*s.

### 3.3. Fermentation and Extraction

The fungal strain was cultured in Erlenmeyer flasks containing either YMG or rice media. For submerged YMG cultivation, fermentation was carried out in 18 × 1 L shaker flasks containing 400 mL of medium (10 g/L malt extract, 4 g/L D-glucose, 4 g/L yeast extract, pH 6.3 before autoclaving), as previously described [6], with each inoculum consisting of 10 well-grown mycelial plugs. The cultures were incubated under shaking conditions in the dark at 140 rpm and 23 °C, and the fermentation process was monitored by checking the concentration of free glucose with Medi-Test glucose (Macherey-Nagel, Düren, Germany). The free glucose was fully consumed after 45 days, and extraction was performed after 3 days of glucose depletion. Alternatively, the rice substrate cultures were cultivated as previously reported [15]. Basically, 10 × 500 mL Erlenmeyer flasks consisting of 90 mg of rice in 90 mL distilled water were prepared and autoclaved. These were used to inoculate fungal plugs, as similarly carried out for the YMG cultures. However, the rice medium cultures were cultivated under static conditions at 23 °C, and the cultures were extracted after 30 days.

To extract the secondary metabolites from the liquid cultures, the supernatant and mycelia were first separated by vacuum filtration. The supernatant was decanted with an equal amount of EtOAc in a separatory funnel. The obtained organic phase was filtered through anhydrous sodium sulfate and the filtrate was evaporated to dryness under a vacuum at 40 °C with a rotary evaporator (Heidolph Instruments GmbH & Co. KG, Schwabach, Germany; pump: Vacuubrand GmbH & Co. KG, Wertheim am Main, Germany) in order to produce a solid residue of the total extract. The secondary metabolites from the mycelia (from either submerged or rice medium cultures) were extracted by initial soaking the mycelia in acetone, followed by immediate sonication for 30 min at 40 °C using an ultrasonic bath (Sonorex Digital 10 P, Bandelin Electronic GmbH & Co. KG, Berlin, Germany). The acetone was evaporated under reduced pressure at 40 °C, the resulting aqueous phase was decanted with an equal amount of ethyl acetate, and the total extract was obtained, as previously described for the supernatant phase. The overall process yielded 881 mg, 367 mg, and 1.6 g of supernatant, mycelia, and rice crude extracts, respectively.

### 3.4. Isolation of Compounds **1**–**5**

To further separate the compounds, the mycelial and supernatant crude extracts were first combined due to their similar chemical profiles. The total extract (1.25 g) was dissolved in methanol (MeOH) and pre-fractionated using a Reveleris X2 flash chromatography system (W.R. Grace and Co., Columbia, MD, USA) equipped with a 40 g silica pre-packed column (Reveleris^®^). Dichloromethane (CH_2_Cl_2_) (solvent A) and CH_2_Cl_2_:MeOH (ratio 8:2) (solvent B) were used as eluents, with a flow rate of 60 mL/min. The gradient of separation started with 0% to 30% B in 30 min, isocratic holding at 30% B for 2 min, 30% to 60% B in 15 min, isocratic holding at 60% B for 2 min, and 60% to 100% B in 10 min. UV detections were obtained at 190, 210, and 280 nm, and several fractions were obtained from this separation and further purified on the Gilson preparative reversed-phase HPLC (PLC 2020, Gilson, Middleton, WI, USA). A Synergi^TM^ 10 µm Polar-RP 80 Å (250 × 50 mm) AXIA™ packed column (Phenomenex Inc., Aschaffenburg, Germany) was used as the stationary phase. Deionized H_2_O + 0.1% formic FA (*v*/*v*) (solvent A) and acetonitrile (MeCN) + 0.1% FA (*v*/*v*) (solvent B) were used as the mobile phase with a flow rate of 40 mL/min. Separation was carried out with an elution gradient beginning isocratically at 5% solvent B for 10 min, followed by a gradient increase to 65% B in 30 min, then an increase from 65% B to 100% B in 10 min, and ending with an isocratic hold at 100% B for 15 min. UV detection was performed at 190, 210, and 280 nm to yield compounds **1** (*t*_R_ = 14.0 min), **3** (*t*_R_ = 12.0 min), and **2** (*t*_R_ = 16.0 min). The purity of the fractions was checked using HPLC-DAD-ESI-MS.

The rice crude extract was divided into two portions. One portion was fractionated directly on the Gilson system after initially passing it through a RP solid-phase cartridge (Strata-X 33 μm Polymeric Reversed Phase; Phenomenex, Aschaffenburg, Germany) to remove fatty acids. A similar solvent system and UV detections were used on the instrument as mentioned for the YMG extract. However, the stationary phase in this case was a C_18_ VP-Nucleodur column 100-5 (250 × 40 mm, 7 μm: Machery-Nagel, Düren, Germany). The gradient was operated with isocratic conditions at 5% B for 10 min, followed by an increase from 5% B to 10% B in 10 min, from 10% B to 80% B in 40 min, from 80% to 100% B in 5 min, and a final isocratic step at 100% B for 10 min. This yielded compounds **4** (*t*_R_ = 41.0 min) and **5** (*t*_R_ = 55.0 min).

Dentifragilone A (**1**): White amorphous solid; 1.14 mg; ${\left[\alpha \right]}_{D}^{20}$ = +69° (*c* 0.1, methanol); UV/Vis (MeOH): λ_max_ (log *ε*) = 196.6 (1.7), 279.6 (0.6) nm; NMR data (^1^H NMR: 500 MHz, ^13^C NMR: 125 MHz in chloroform-*d*) see Table 1; HR-(+)ESIMS: *m/z* 209.1167 [M-H_2_O+H]^+^ (calcd. 209.1172 for C_12_H_17_O_3_^+^), 227.1277 [M+H]^+^ (calcd. 227.1278 for C_12_H_19_O_4_^+^), 249.1098 [M+Na]^+^ (calcd. 249.1097 for C_12_H_18_NaO_4_^+^); *t*_R_ = 3.90 min (HR-LC-ESIMS). C_12_H_18_O_4_ (226.11 g/mol).

Dentifragilone B (**2**): Off-white amorphous solid; 0.84 mg; UV/Vis (MeOH): λ_max_ (log *ε*) = 196.6 (1.7); NMR data (^1^H NMR: 500 MHz, ^13^C NMR: 125 MHz in chloroform-*d* and methanol-*d_4_*) see Table 2; HR-(+)ESIMS: *m/z* 225.1117 [M+H]^+^ (calcd. 225.1121 for C_12_H_17_O_4_^+^), 247.0937 [M+Na]^+^ (calcd. 247.0941 for C_12_H_16_NaO_4_^+^); *t*_R_ = 4.04 min (HR-LC-ESIMS). C_12_H_16_O_4_ (224.09 g/mol).

Dentipellinol (**3**): Off-white amorphous solid; 0.62 mg; ${\left[\alpha \right]}_{D}^{20}$ = –42° (*c* 0.1, methanol); UV/Vis (MeOH): λ_max_ (log *ε*) = 196.6 (1.7); NMR data (^1^H NMR: 700 MHz, ^13^C NMR: 125 MHz in chloroform-*d*) see Table S1 comparable to the reported literature [5]; *m/z* 221.0805 [M-H_2_O+H]^+^ (calcd. 221.0808 for C_12_H_13_O_4_^+^), 239.0911 [M+H]^+^ (calcd. 239.0914 for C_12_H_15_O_5_^+^), 261.0734 [M+Na]^+^ (calcd. 261.0733 for C_12_H_14_NaO_5_^+^); *t*_R_ = 2.86 min (HR-LC-ESIMS). C_12_H_14_O_5_ (238.10 g/mol).

Methyl 4-chloro-3,5-dimethoxybenzoate (**4**): Off-white amorphous solid; 5.43 mg; UV/Vis (MeOH): λ_max_ = 217, 263, 301 nm; NMR data (^1^H NMR: 500 MHz, ^13^C NMR: 125 MHz in chloroform-*d*) comparable to the reported literature [14]; HR-(+)ESIMS: *m/z* 231.4012 [M+H]^+^ (calcd. 231.4019 for C_10_H_12_ClO_4_^+^), 253.0236 [M+Na]^+^ (calcd. 253.0238 for C_10_H_11_ClNaO_4_^+^); *t*_R_ = 8.56 min (HR-LC-ESIMS). C_10_H_11_ClO_4_ (230.37 g/mol).

10-Methoxycarbonyl-10-norisodrimenin (**5**): Off-white amorphous solid; 1.43 mg; UV/Vis (MeOH): λ_max_ = 219 nm; NMR data (^1^H NMR: 500 MHz, ^13^C NMR: 125 MHz in chloroform-*d*) comparable to the reported literature [16]; HR-(+)ESIMS: *m/z* 279.1588 [M+H]^+^ (calcd. 279.1591 for C_16_H_23_O_5_^+^), 301.1411 [M+Na]^+^ (calcd. 301.1410 for C_16_H_22_NaO_5_^+^); *t*_R_ = 9.75 min (HR-LC-ESIMS). C_16_H_22_O_5_ (278.14 g/mol).

### 3.5. Antimicrobial Assay

The Minimum Inhibitory Concentration (MIC) of the isolated compounds was determined following the method previously described [16]. Accordingly, the compounds were tested against bacteria and fungi on a serial dilution assay performed on 96-well microtiter plates. YMG medium was used to culture the yeasts and filamentous fungi, whereas MHB media (Müller–Hinton Broth: SNX927.1, Carl Roth GmbH, Karlsruhe, Germany) was used for bacteria.

### 3.6. Cytotoxicity Assay

The in vitro cytotoxicity (IC_50_) of isolated compounds was evaluated against an array of mammalian cell lines using a colorimetric tetrazolium dye MTT assay with epothilone B as a positive control. The cell lines, L929 (mouse fibroblasts) and KB3.1 (human endocervical adenocarcinoma), were employed, following established methodologies [16].

### 3.7. Density Functional Theory Calculations

In order to elucidate the electronic circular dichroism (ECD) spectra, a conformational analysis was principally executed to extract all possible conformations of compounds **1** and **2**, employing Omega2 software 2.5.1.4 [17] within an energy window value of 10 kcal/mol [18]. The resulting configurations were set to the geometry optimization process and then frequency computations at the B3LYP/6-31G* level of theory. The time-dependent density functional theory (TDDFT) calculations were then performed in methanol to determine the first fifty excitation states. The solvent effect was incorporated using the polarizable continuum model (PCM). The calculated ECD spectra were graphed using the SpecDis 1.71 [18,19]. The extracted ECD spectra were finally Boltzmann-averaged. All quantum calculations were performed using Gaussian09 software [20].

## 4. Conclusions

Two previously undescribed and two known benzoic acid derivatives (**1**–**4**), in addition to a previously isolated drimane sesquiterpenoid (**5**), were derived from submerged and solid-state cultures of *D. fragilis*. The fungus is rarely encountered in temperate zones and has proven to be a significant source of novel chemical components. However, compounds **1** and **2**, herein isolated, were unprecedented. Thus, it would not be surprising to discover new useful compounds from *D. fragilis* in the future. Although only moderate to weak antibiotics effects of the compounds have been realized, the compounds are potential candidates for alternative bioactivity studies that were not attained within the realm of the current study. Our findings also provide further insights into the chemodiversity of *D. fragilis*, highlighting the potential role of alternative media components in the versatile production of secondary metabolites.

## Figures and Tables

**Table 1:** (^1^H and ^13^C) 1D NMR data of **1**.

pos.	δ_C_, *^a^*^,*c*^ Type	δ_H_ *^b^* Multi (*J*[Hz])
1	195.8, CO	
2	144.0, C	
3	130.0, C	
4	36.7, CH_2_	*α* 2.56 dd (18.4, 5.8) *β* 2.74 dd (18.4, 9.8)
5	75.7, CH	4.18 ddd (9.4, 5.8, 2.8)
6	80.4, C	
7	44.8, C	
8	143.6, CH	6.04 dd (17.4, 10.7)
9	113.2, CH_2_	*α* 5.02 d (10.7) *β* 5.09 d (17.4)
10	17.0, CH_3_	1.92 s
11	24.4, CH_3_	1.07 s
12	23.9, CH_3_	1.26 s
1-OH	-	-
2-OH	-	5.76 s
5-OH	-	3.44 br s
6-OH	-	2.37 br s

Measured in chloroform-*d* at *^a^* 125/*^b^* 500 MHz. *^c^* Assignment confirmed by HMBC and HSQC spectra.

**Table 2:** (^1^H and ^13^C) 1D NMR data of **2**.

pos.	δ_C_, *^a^*^,*e*^ Type	δ_H_ *^b^* multi (*J*[Hz])	δ_C_, *^c^*^,*e*^ Type	δ_H_ *^d^* multi (*J*[Hz])
1	162.3, C		163.0, C	
2	119.2, C		121.6, C	
3	128.5, CH	7.75 d (2.1)	131.1, CH	7.91 d (2.1)
4	131.4, C		133.4, C	
5	135.8, CH	7.49 dd (8.5, 2.1)	136.8, CH	7.51 dd (8.5, 2.1)
6	118.7, CH	7.00 d (8.5)	119.1, CH	6.93 d (8.5)
7	64.2, CH_2_	4.66 s	64.5, CH_2_	4.57 s
8	207.2, CO		207.5, CO	
9	48.1, CH_2_	3.20 s	50.8, CH_2_	3.22 s
10	70.0, C		71.3, C	
11,12	29.4, CH_3_	1.37 s	29.9, CH_3_	1.36 s
1-OH	-	12.13 br s	-	-

Measured in chloroform-*d ^a^* at 125 MHz/*^b^* at 500 MHz. Measured in methanol-*d*_4_ *^c^* at 125 MHz/*^d^* at 500 MHz. *^e^* Assignment confirmed by HMBC and HSQC spectra.

## Data Availability

Data are contained within the article and Supplementary Materials.

## Supplementary Materials

The following supporting information can be downloaded at https://www.mdpi.com/article/10.3390/molecules29122859/s1: Figures S1–S8: HPLC, LR-/HR-ESI-MS, 1D/2D NMR spectra of **1**; Figure S9–S20: HPLC, LR-/HR-ESI-MS, 1D/2D NMR spectra of **2**; Figure S21–S29: HPLC, LR-/HR-ESI-MS, 1D/2D NMR, ECD spectra of **3**; Table S1: 1D (^1^H and ^13^C) NMR data of compound **3** and dentipellinol; Figures S30–S37: HPLC, LR-/HR-ESI-MS, 1D/2D NMR spectra of **4**; Figures S38–S45: HPLC, LR-/HR-ESI-MS, 1D/2D NMR spectra of **5**.
