# Supplementary material for: Dentifragilones A–B and Other Benzoic Acid Derivatives from the European Basidiomycete Dentipellis fragilis
Source: Molecules. 2024 Jun 16;29(12):2859. doi: 10.3390/molecules29122859 (PMC11206269; doi:10.3390/molecules29122859)
Supplement: Supplementary file 1 [file molecules-29-02859-s001.zip › molecules-3042948-supplementary-1.pdf]

SUPPLEMENTARY MATERIALS FOR:

Dentifragilones A–B and Other Benzoic Acid Derivatives From the European Basidiomycete *Dentipellis fragilis*

Winnie Chemutai Sum[1,2], Sherif S. Ebada[1,3], Mahmoud A. A. Ibrahim[4,5], Harald Kellner[6], and Marc Stadler[1,2]*

[1] Department of Microbial Drugs, Helmholtz Centre for Infection Research GmbH (HZI), Inhoffenstraße 7, 38124 Braunschweig, Germany

[2] Institute of Microbiology, Technische Universität Braunschweig, Spielmannstraße 7, 38106 Braunschweig, Germany

[3] Department of Pharmacognosy, Faculty of Pharmacy, Ain Shams University, 11566 Cairo, Egypt

[4] Computational Chemistry Laboratory, Chemistry Department, Faculty of Science, Minia University, Minia 61519, Egypt

[5] School of Health Sciences, University of KwaZulu-Natal, Westville, Durban 4000, South Africa

[6] Department of Bio- and Environmental Sciences, Technische Universität Dresden-International Institute Zittau, Markt 23, 02763 Zittau, Germany

* Correspondence: *Marc.Stadler@helmholtz-hzi.de* (M.S.); Tel.: +49-531-6181-4240; Fax +49-531-6181-9499

TABLE OF CONTENTS

#	Contents	Page
**1**	Figure S1. LR-ESI-MS of **1**.	**S4**
**2**	Figure S2. HR-ESI-MS of **1**.	**S5**
**3**	Figure S3. ^{1}H NMR spectrum of **1** in chloroform-*d* at 500 MHz.	**S6**
**4**	Figure S4. ^{13}C NMR spectrum of **1** in chloroform-*d* at 125 MHz.	**S7**
**5**	Figure S5. ^{1}H-^{1}H COSY spectrum of **1** in chloroform-*d* at 500 MHz.	**S8**
**6**	Figure S6. HMBC spectrum of **1** in chloroform-*d* at 500 MHz.	**S9**
**7**	Figure S7. HSQC spectrum of **1** in chloroform-*d* at 500 MHz.	**S10**
**8**	Figure S8. ROESY spectrum of **1** in chloroform-*d* at 500 MHz.	**S11**
**9**	Figure S9. LR-ESI-MS of **2**.	**S12**
**10**	Figure S10. HR-ESI-MS of **2**.	**S13**
**11**	Figure S11. ^{1}H NMR spectrum of **2** in chloroform-*d* at 500 MHz.	**S14**
**12**	Figure S12. ^{1}H-^{1}H COSY spectrum of **2** in chloroform-*d* at 125 MHz.	**S15**
**13**	Figure S13. HMBC spectrum of **2** in chloroform-*d* at 500 MHz.	**S16**
**14**	Figure S14. HSQC spectrum of **2** in chloroform-*d* at 500 MHz.	**S17**
**15**	Figure S15. ^{1}H NMR spectrum of **2** in methanol-d_4 at 500 MHz.	**S18**
**16**	Figure S16. DEPTQ spectrum of **2** in methanol-d_4 at 125 MHz.	**S19**
**17**	Figure S17. ^{1}H-^{1}H COSY spectrum of **2** in methanol-d_4 at 500 MHz.	**S20**
**18**	Figure S18. HMBC spectrum of **2** in methanol-d_4 at 500 MHz.	**S20**
**19**	Figure S19. HSQC spectrum of **2** in methanol-d_4 at 500 MHz.	**S21**
**20**	Figure S20. ROESY spectrum of **2** in methanol-d_4 at 500 MHz.	**S22**
**21**	Figure S21. LR-ESI-MS of **3**.	**S23**
**22**	Figure S22. HR-ESI-MS of **3**.	**S24**
**23**	Figure S23. ^{1}H NMR spectrum of **3** in chloroform-*d* at 700 MHz.	**S25**
**24**	Figure S24. DEPTQ NMR spectrum of **3** in chloroform-*d* at 175 MHz..	**S26**
**25**	Figure S25. ^{1}H-^{1}H COSY spectrum of **3** in chloroform-*d* at 700 MHz.	**S27**
**26**	Figure S26. HMBC spectrum of **3** in chloroform-*d* at 700 MHz.	**S28**
**27**	Figure S27. HSQC spectrum of **3** in chloroform-*d* at 700 MHz.	**S29**
**28**	Figure S28. ROESY spectrum of **3** in chloroform-*d* at 700 MHz.	**S30**
**29**	Table S1. 1D (^{1}H and ^{13}C) NMR data of compound **3** and dentipellinol.	**S31**
**30**	Figure S29. Experimental and calculated ECD spectra of **3**.	**S32**
**31**	Figure S30. LR-ESI-MS of **4**.	**S33**
**32**	Figure S31. HR-ESI-MS of **4**.	**S34**
**33**	Figure S32. ^{1}H NMR spectrum of **4** in chloroform-*d* at 500 MHz.	**S35**
**34**	Figure S33. DEPTQ spectrum of **4** in chloroform-*d* at 125 MHz.	**S36**
**35**	Figure S34. ^{1}H-^{1}H COSY spectrum of **4** in chloroform-*d* at 500 MHz.	**S37**

**36**	Figure S35. HMBC spectrum of **4** in chloroform-*d* at 500 MHz.	**S38**
**37**	Figure S36. HSQC spectrum of **4** in chloroform-*d* at 500 MHz.	**S39**
**38**	Figure S37. ROESY spectrum of **4** in chloroform-*d* at 500 MHz.	**S40**
**39**	Figure S38. LR-ESI-MS of **5**.	**S41**
**40**	Figure S39. HR-ESI-MS of **5**.	**S42**
**41**	Figure S40. ^{1}H NMR spectrum of **5** in chloroform-*d* at 500 MHz.	**S43**
**42**	Figure S41. ^{13}C NMR spectrum of **5** in chloroform-*d* at 125 MHz.	**S44**
**43**	Figure S42. ^{1}H-^{1}H COSY spectrum of **5** in chloroform-*d* at 500 MHz.	**S45**
**44**	Figure S43. HMBC spectrum of **5** in chloroform-*d* at 500 MHz.	**S46**
**45**	Figure S44. HSQC spectrum of **5** in chloroform-*d* at 500 MHz.	**S47**
**46**	Figure S45. ROESY spectrum of **5** in chloroform-*d* at 500 MHz.	**S48**

Display Report

Analysis Info

		Acquisition Date	24.03.2021 16:04:52
Analysis Name	S:\DATA\AmaZon\wsu20_Winnier Sum Chemutai\DENT STMA 17004\Dent YM 6.3\Dent F4F1_RB1_01_27278.d		
Method	27278.m	Operator	esu
Sample Name	Dent F4F1	Instrument	amaZon speed
Comment			

Acquisition Parameter

Ion Source Type	ESI	Ion Polarity	Negative	Alternating Ion Polarity	on
Mass Range Mode	UltraScan	Scan Begin	100 m/z	Scan End	2000 m/z
Accumulation Time	3870 μs	RF Level	100 %	Trap Drive	71.3
SPS Target Mass	1000 m/z	Averages	6 Spectra		

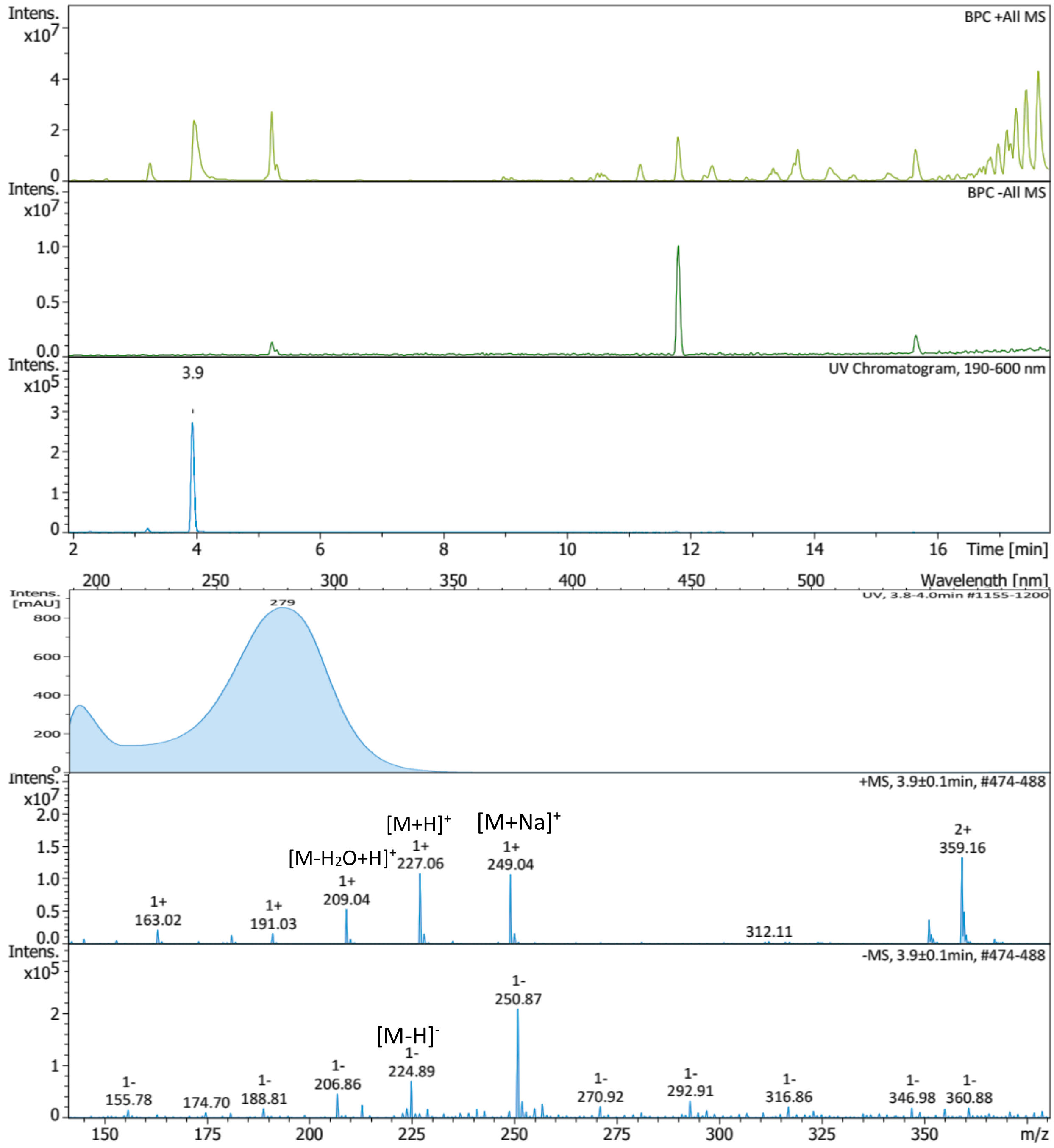

Figure S1. LR-ESI-MS of **1**.

Display Report

Analysis Info

		Acquisition Date	07.06.2021 16:39:26
Analysis Name	F:\Volume D\HZI Projects\Winnie\11-Dentipellis fragilis\Maxis\Dent F4F1_P1-C-2_01_7566.d		
Method	pos_säure_10000_screening_ms_100_2500_line.m	Operator	ate06
Sample Name	Dent F4F1	Instrument	maXis
Comment	Screening01<br>Waters Acquity UPLC BEH C_18 1,7um 2.1x50mm		

Acquisition Parameter

Ion Polarity Positive

SPS Target Mass

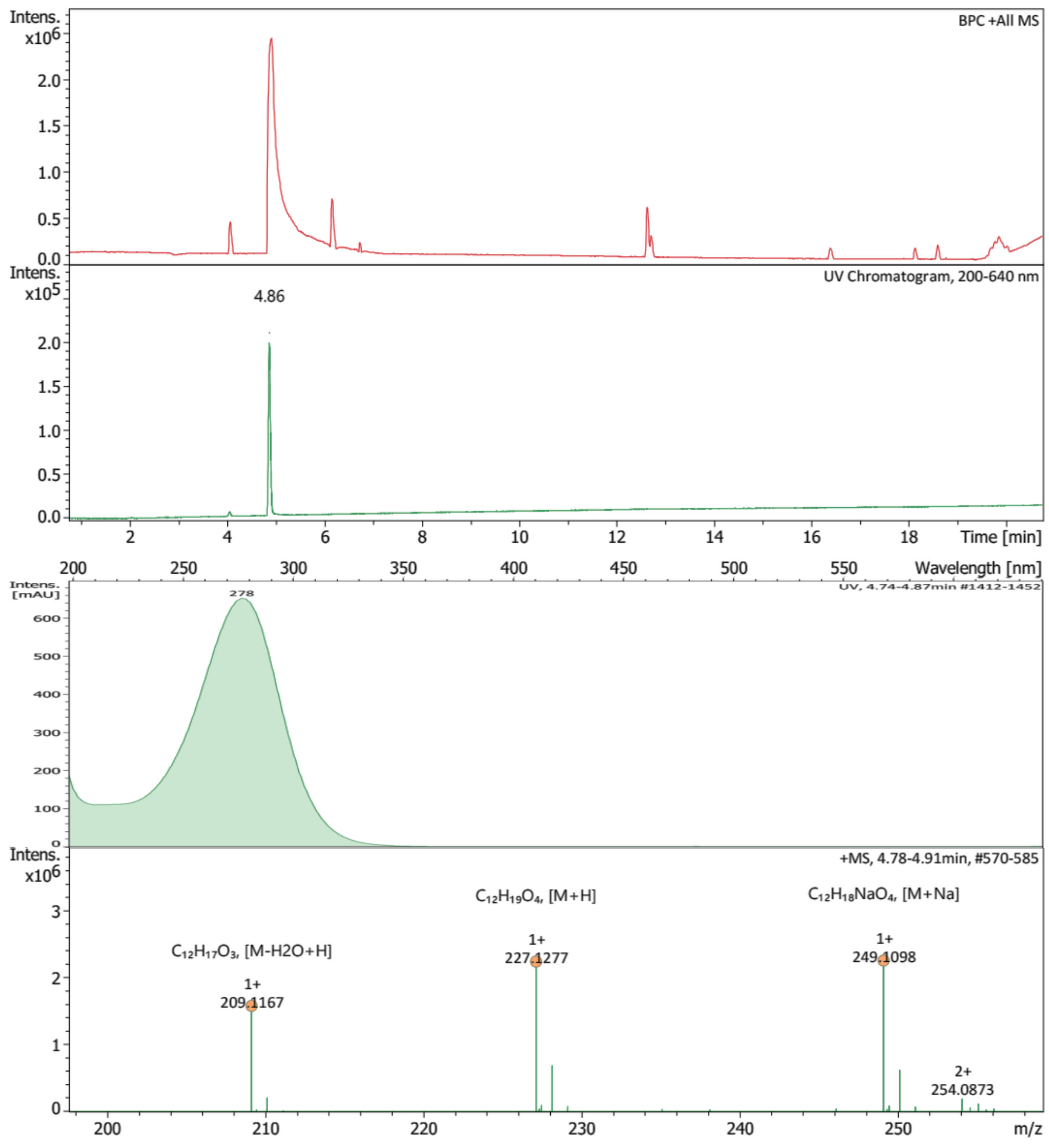

Bruker Compass DataAnalysis 6.1 printed: 10.04.2024 10:34:41 by: sel22 Page 1 of 1

Figure S2. HR-ESI-MS of **1**.

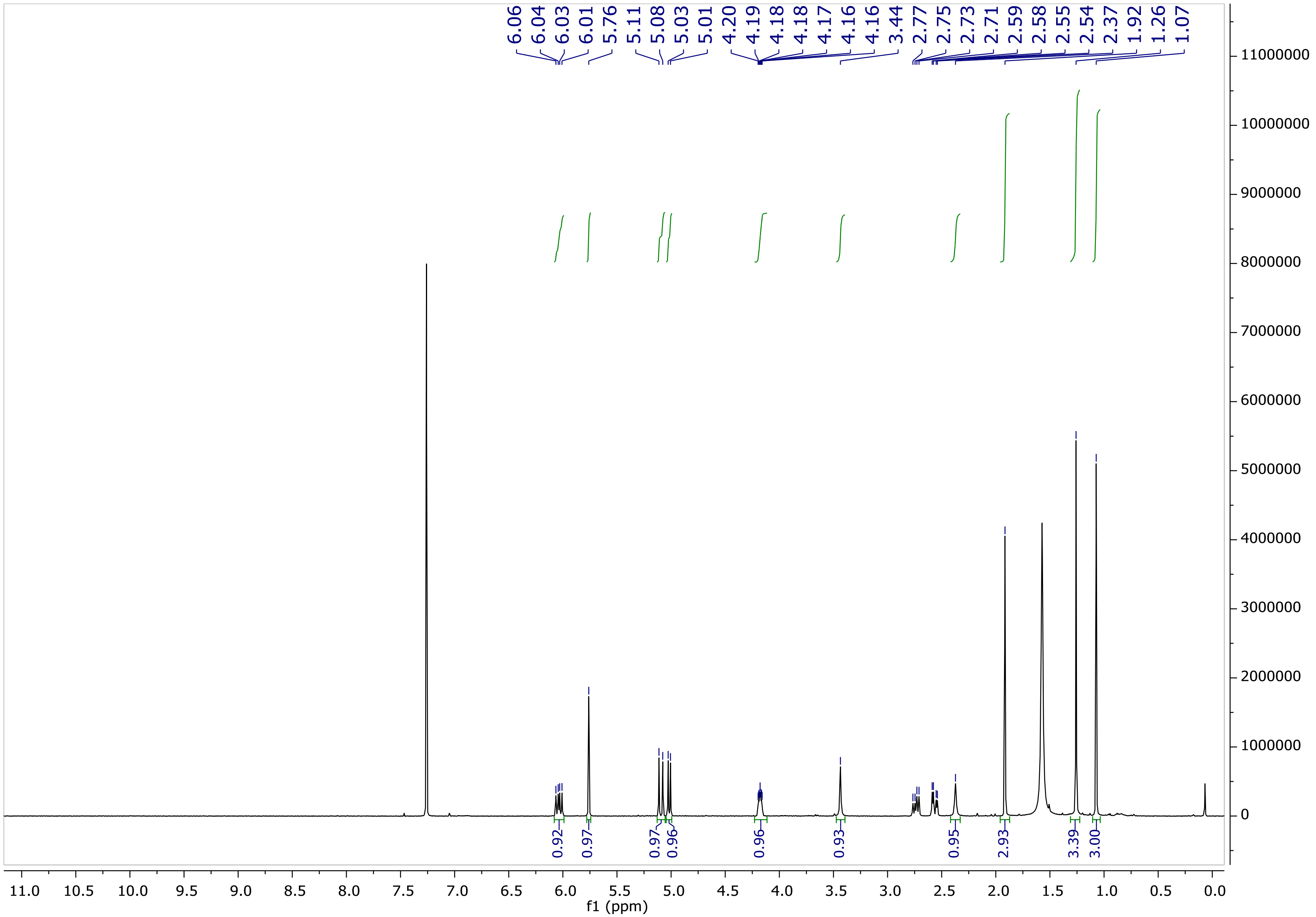

Figure S3. ^{1}H NMR spectrum of **1** in chloroform-*d* at 500 MHz.

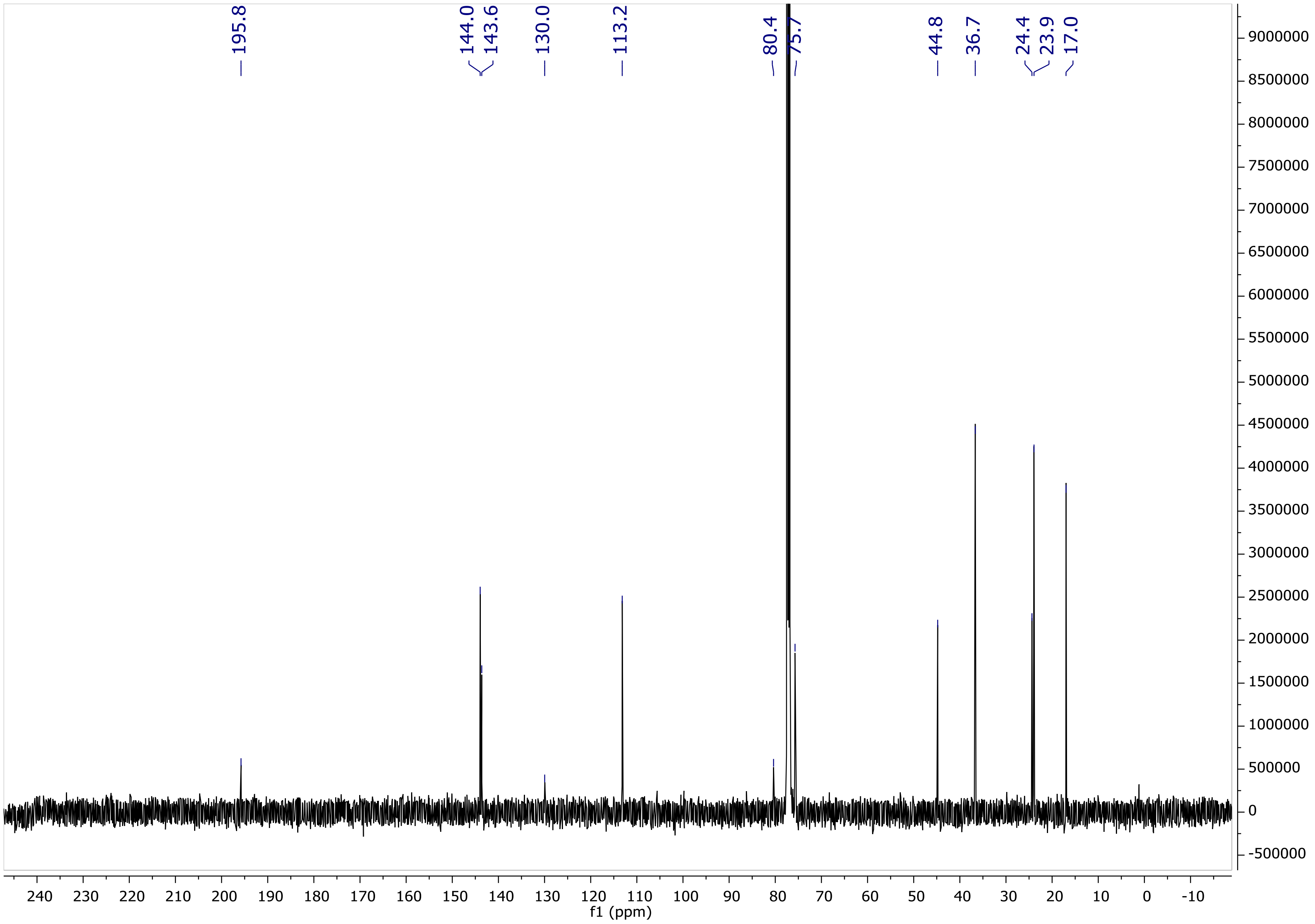

Figure S4. ^{13}C NMR spectrum of **1** in chloroform-*d* at 125 MHz.

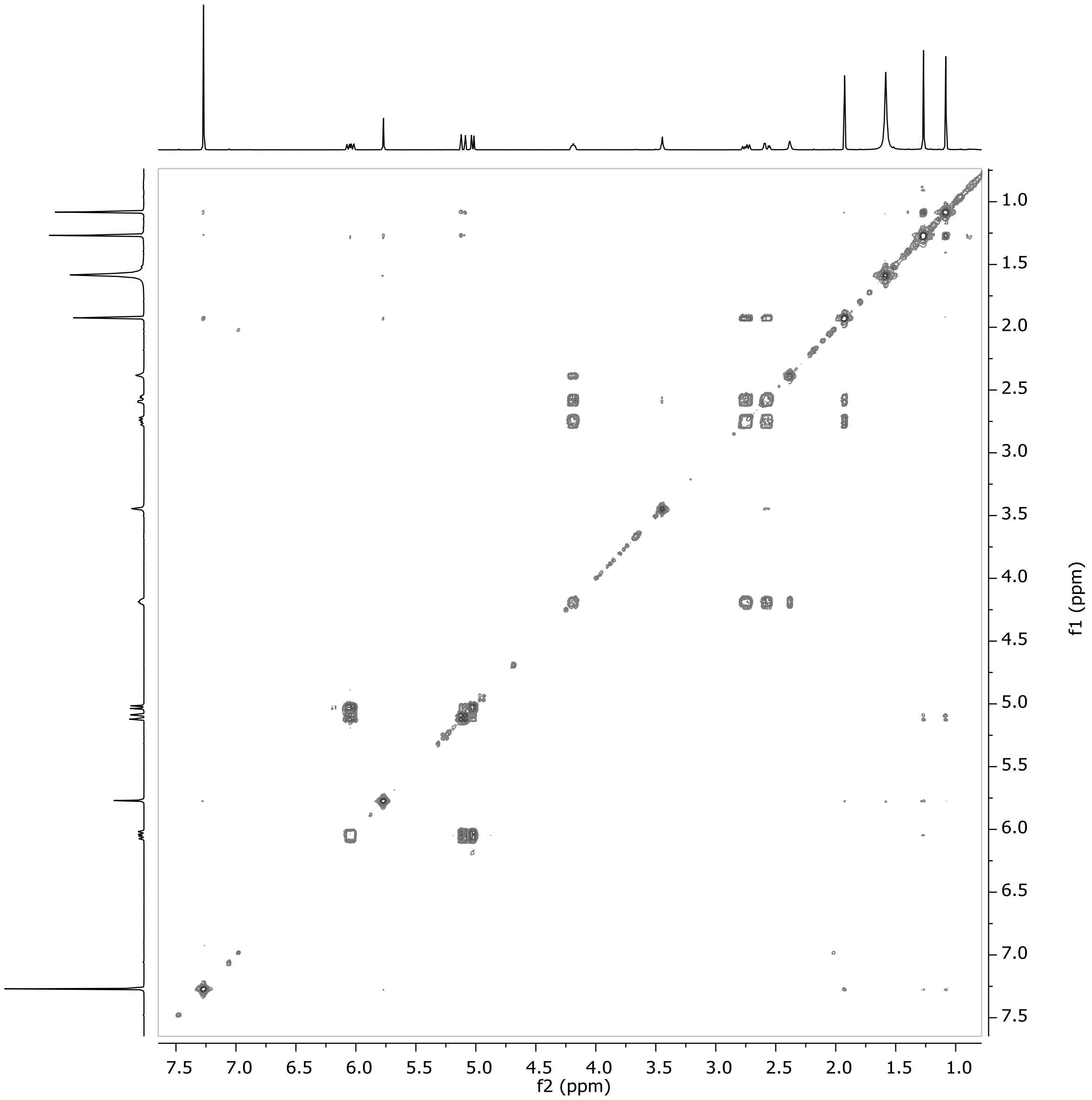

Figure S5. ^{1}H-^{1}H COSY spectrum of **1** in chloroform-*d* at 500 MHz.

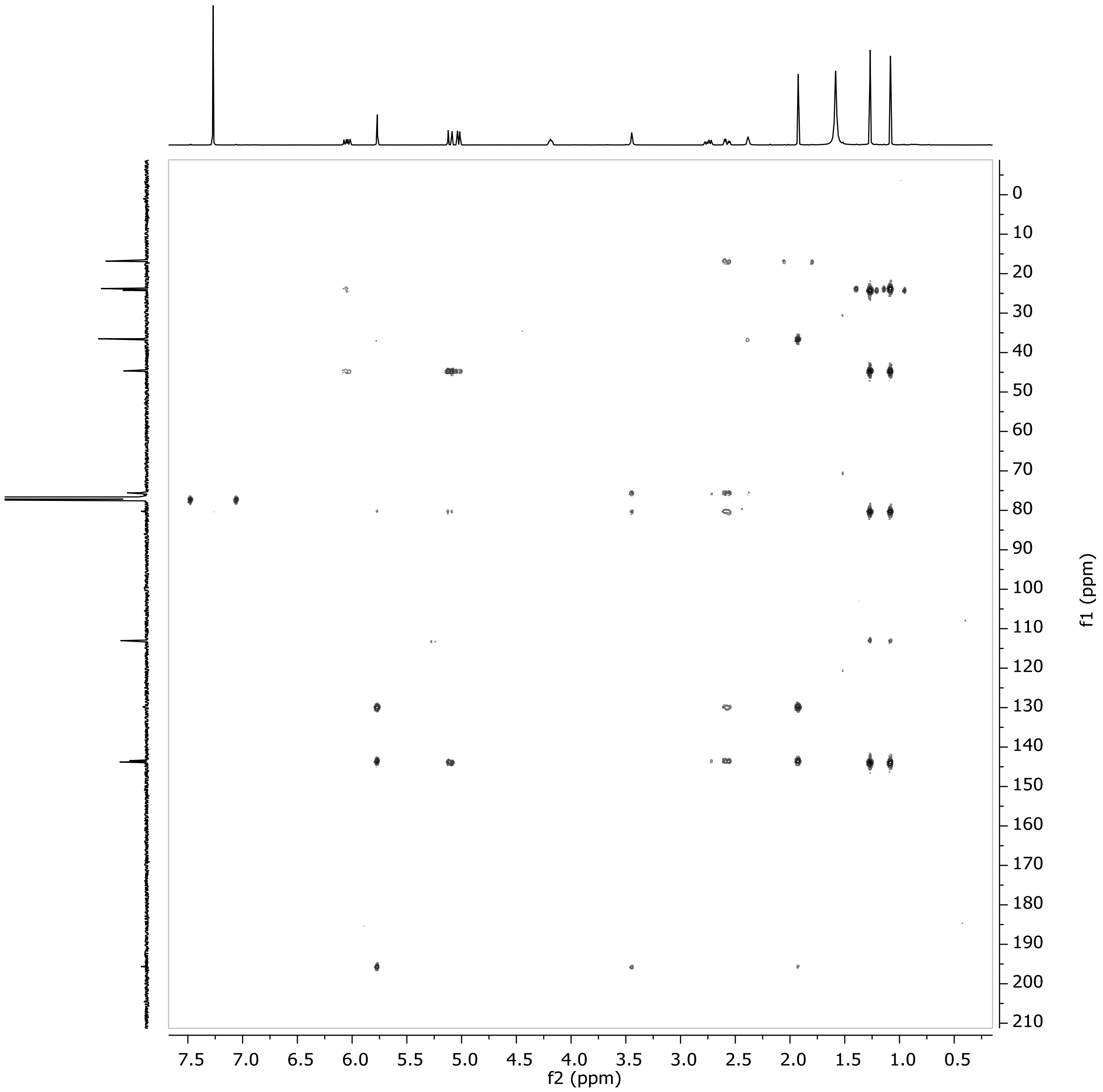

Figure S6. HMBC spectrum of **1** in chloroform-*d* at 500 MHz.

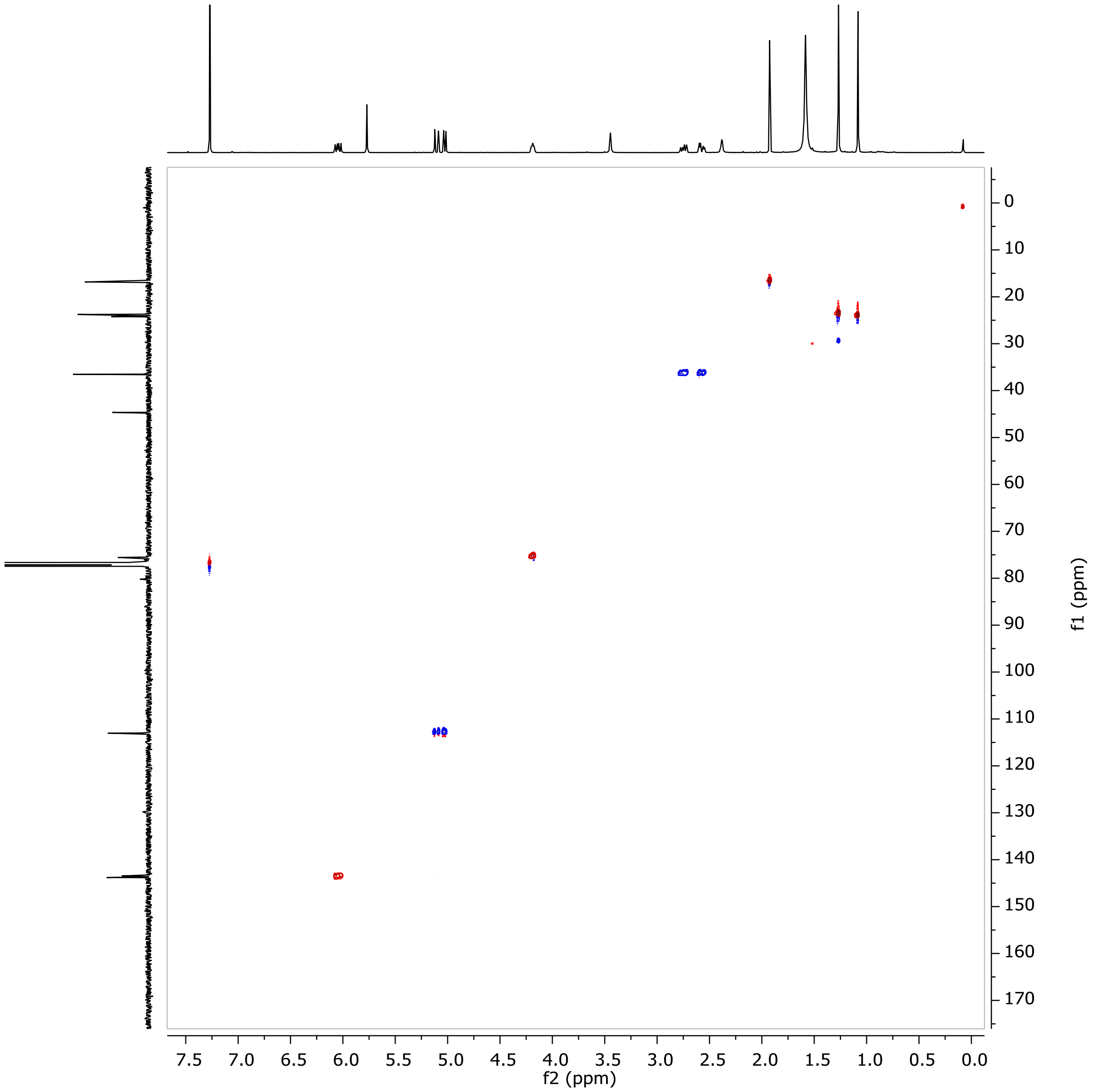

Figure S7. HSQC spectrum of **1** in methanol-d_4 at 500 MHz.

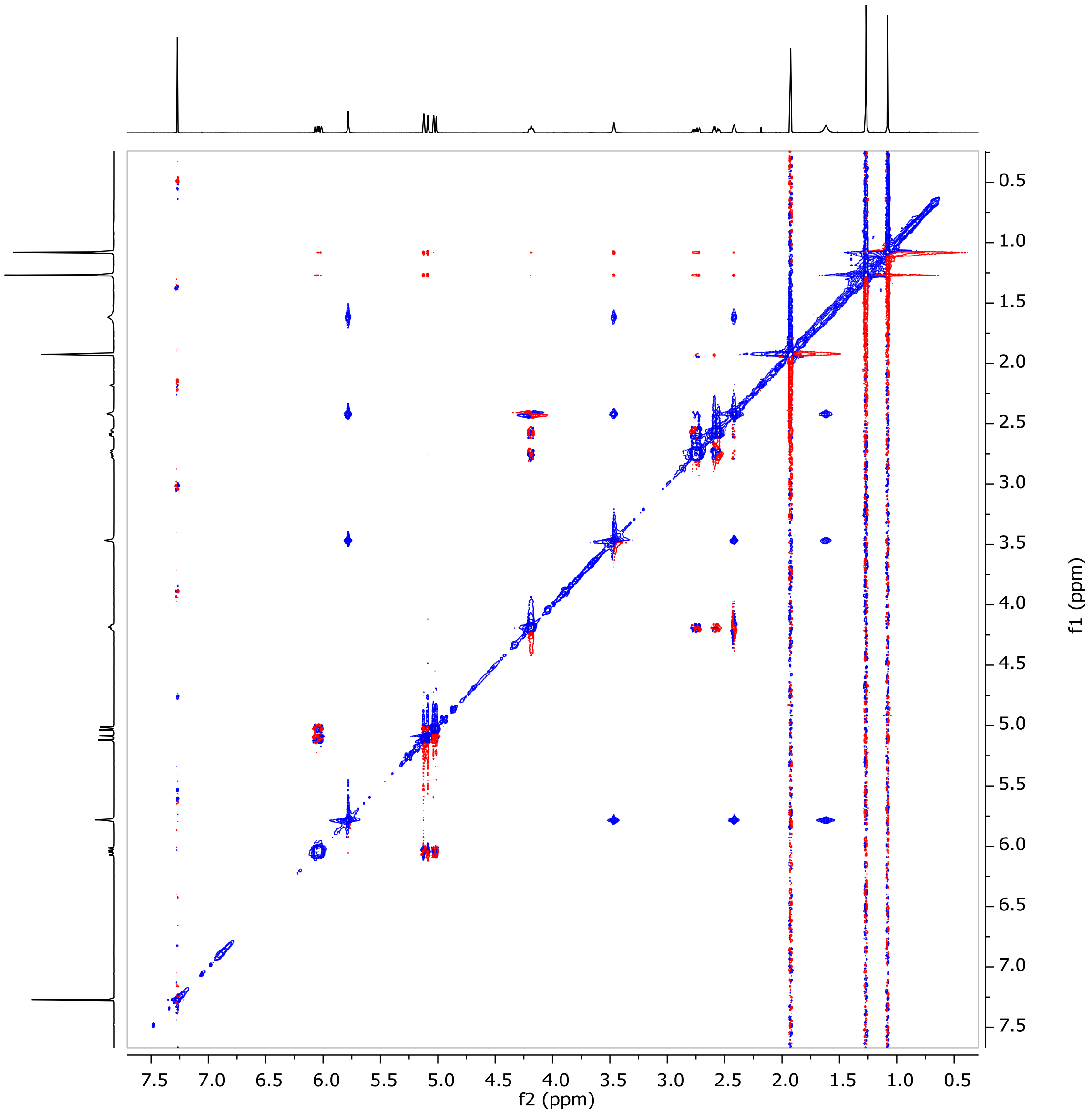

Figure S8. ROESY spectrum of **1** in methanol-d_4 at 500 MHz.

Display Report

Analysis Info

Analysis Name	F:\Volume D\HZI Projects\Winnie\11-Dentipellis fragilis\Amazon\Dent 7B_BA7_01_29109.d	Acquisition Date	28.05.2021 22:23:48
Method	29109.m	Operator	esu
Sample Name	Dent 7B	Instrument	amaZon speed
Comment			

Acquisition Parameter

Ion Source Type	ESI	Ion Polarity	Negative	Alternating Ion Polarity	on
Mass Range Mode	UltraScan	Scan Begin	100 m/z	Scan End	2000 m/z
Accumulation Time	4000 µs	RF Level	100 %	Trap Drive	71.3
SPS Target Mass	1000 m/z	Averages	6 Spectra		

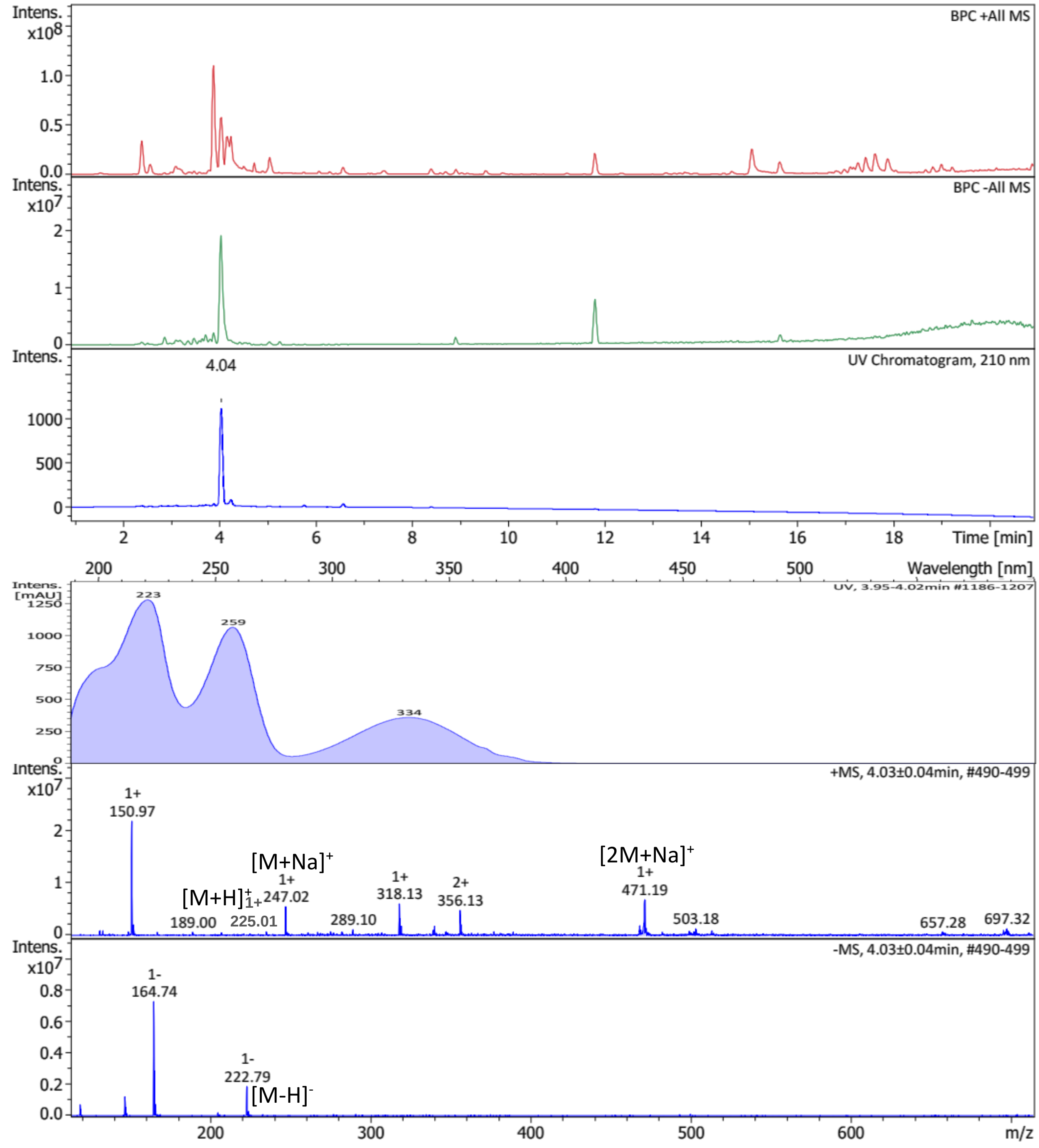

Bruker Compass DataAnalysis 6.1 printed: 10.04.2024 09:36:53 by: sel22 Page 1 of 1

Figure S9. LR-ESI-MS of **2**.

Display Report

Analysis Info

		Acquisition Date	23.02.2022 11:36:13
Analysis Name	S:\DATA\MaXis\wsu20_Winnie Sum Chemutai\22_02\Dent Rice F19_P1-B-1_01_9608.d		
Method	pos_säure_10000_screening_ms_100_2500_line.m	Operator	ate06
Sample Name	Dent Rice F19	Instrument	maXis 255552.00037
Comment	Screening01<br>Waters Acquity UPLC BEH C_18 1,7um 2.1x50mm		

Acquisition Parameter

Source Type	ESI	Ion Polarity	Positive	Set Nebulizer	4.0 Bar
Focus	Not active	Set Capillary	4500 V	Set Dry Heater	200 °C
Scan Begin	50 m/z	Set End Plate Offset	-500 V	Set Dry Gas	10.0 l/min
Scan End	2500 m/z	Set Charging Voltage	0 V	Set Divert Valve	Waste
		Set Corona	0 nA	Set APCI Heater	0 °C

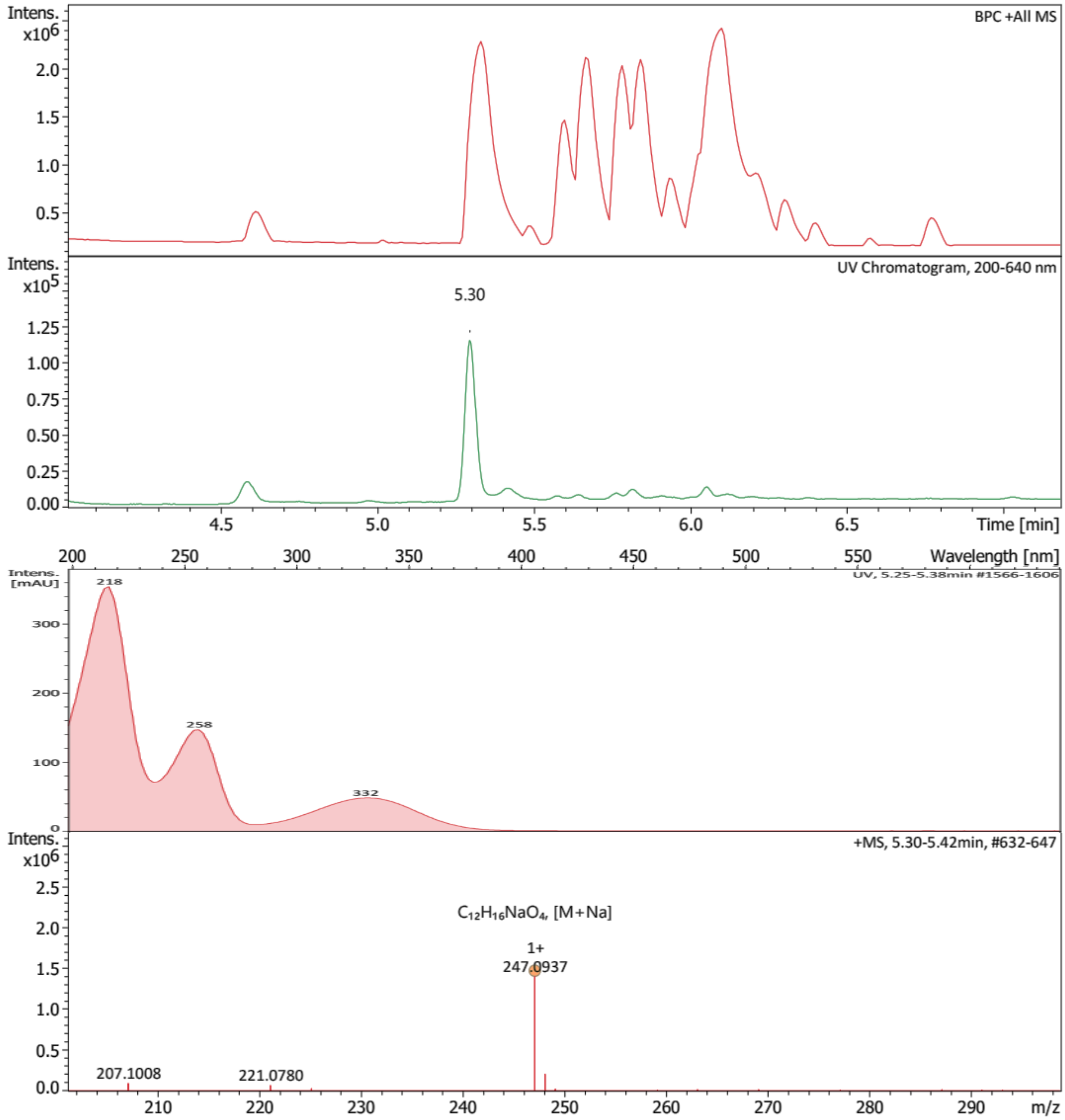

Dent Rice F19_P1-B-1_01_9608.d

Bruker Compass DataAnalysis 6.1 printed: 05.04.2024 10:52:15 by: sel22 Page 1 of 1

Figure S10. HR-ESI-MS of **2**.

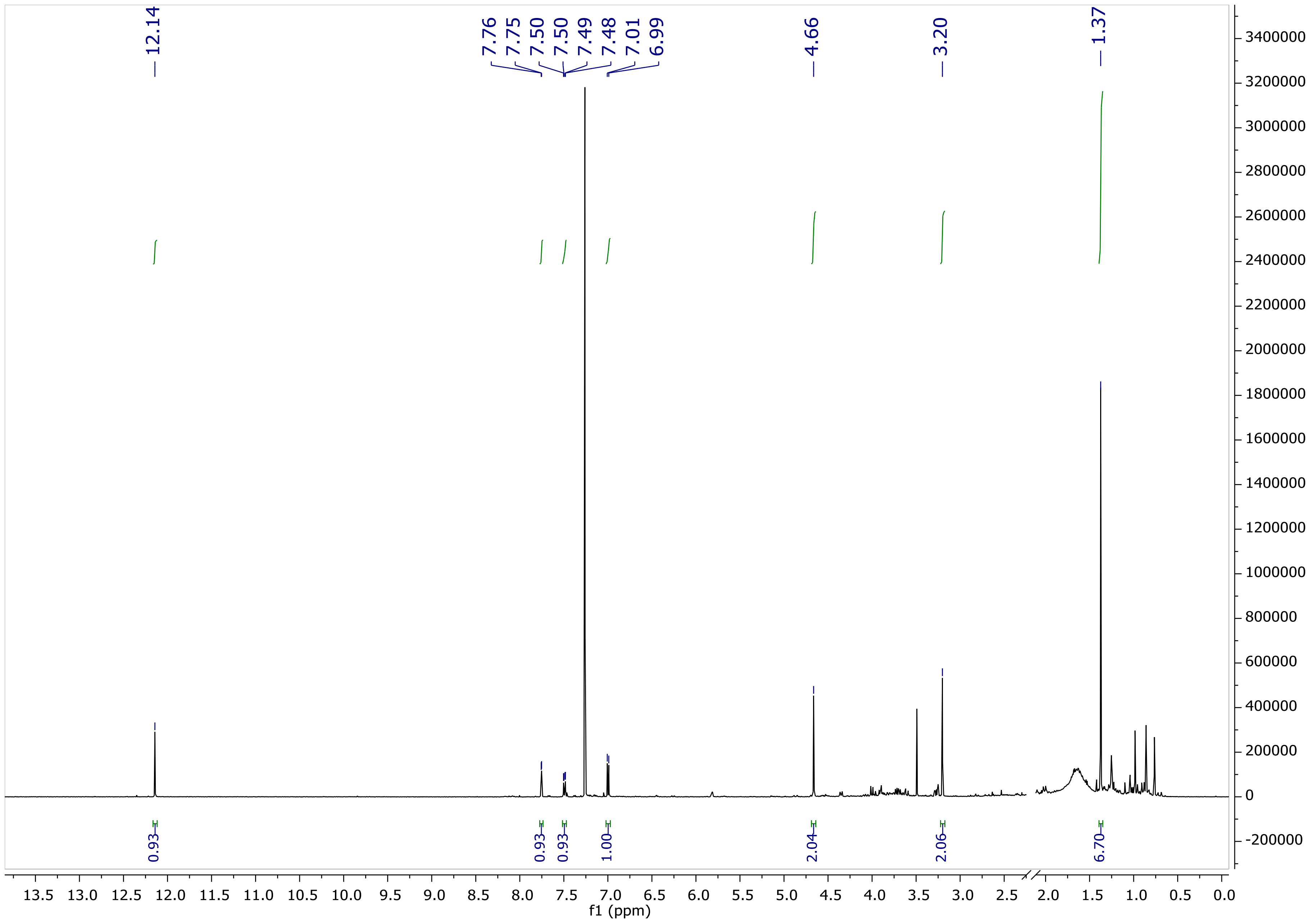

Figure S11. ^{1}H NMR spectrum of **2** in chloroform-*d* at 500 MHz.

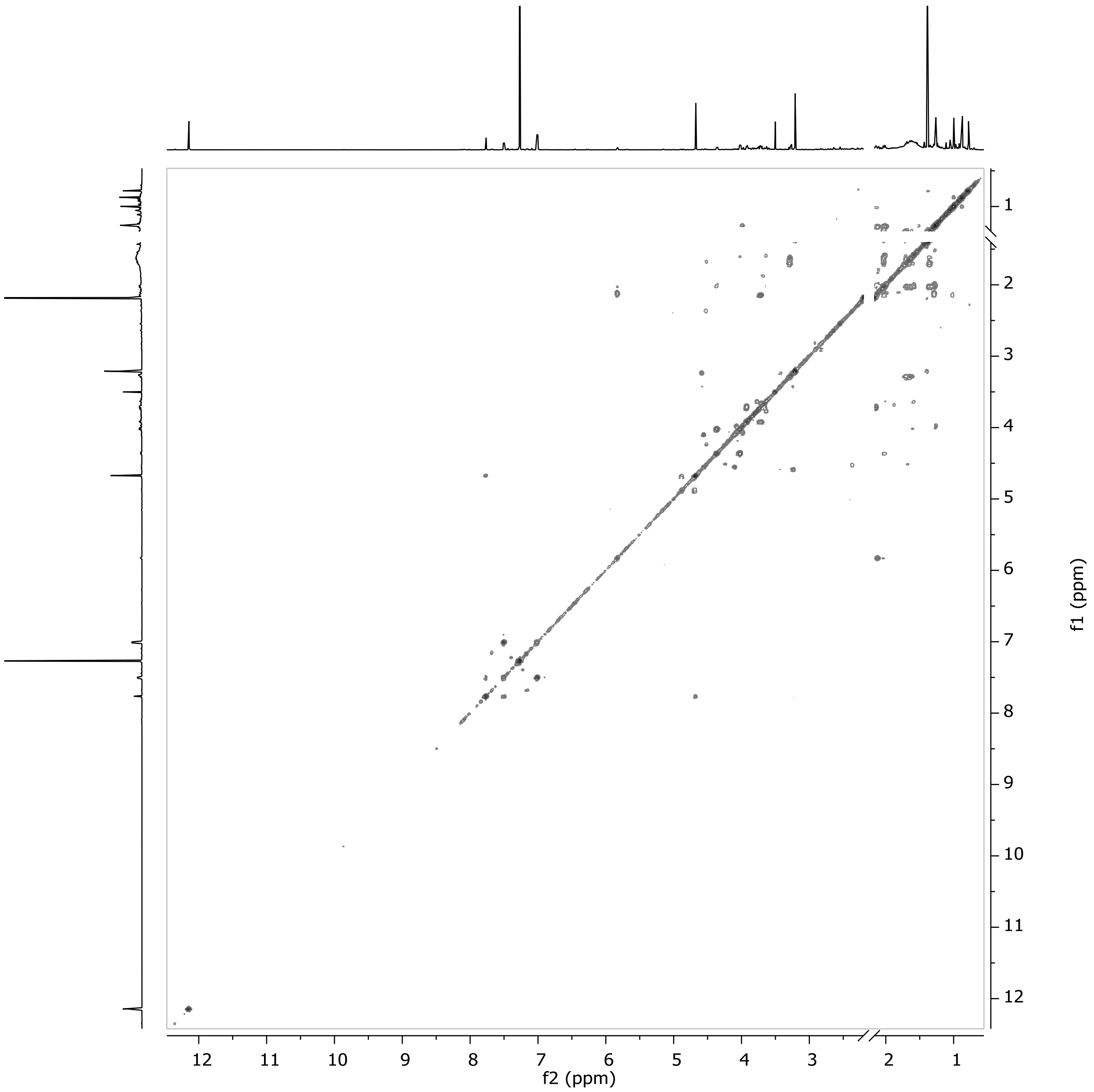

Figure S12. ^{1}H-^{1}H COSY spectrum of **2** in chloroform-*d* at 500 MHz.

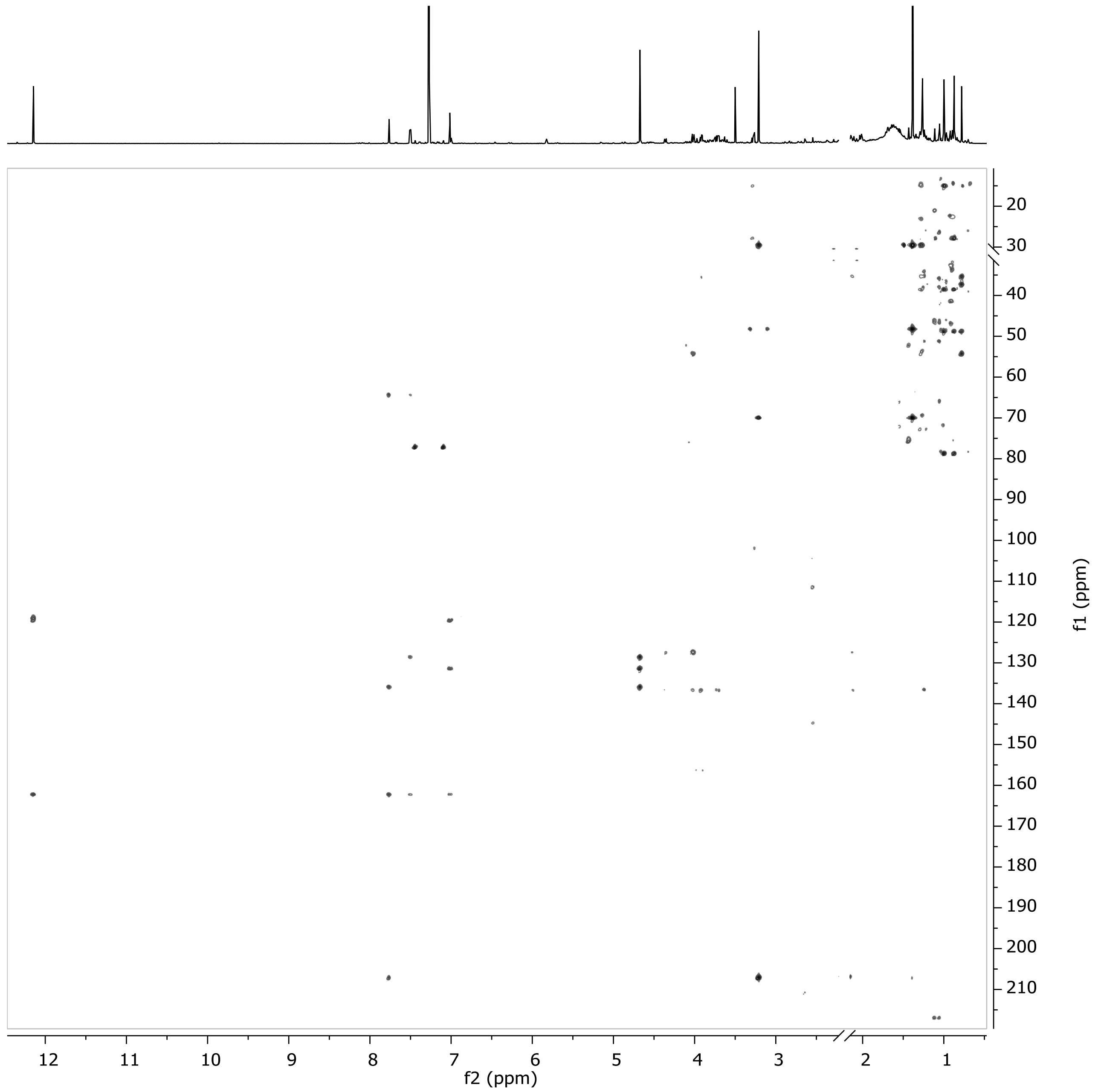

Figure S13. HMBC spectrum of **2** in chloroform-*d* at 500 MHz.

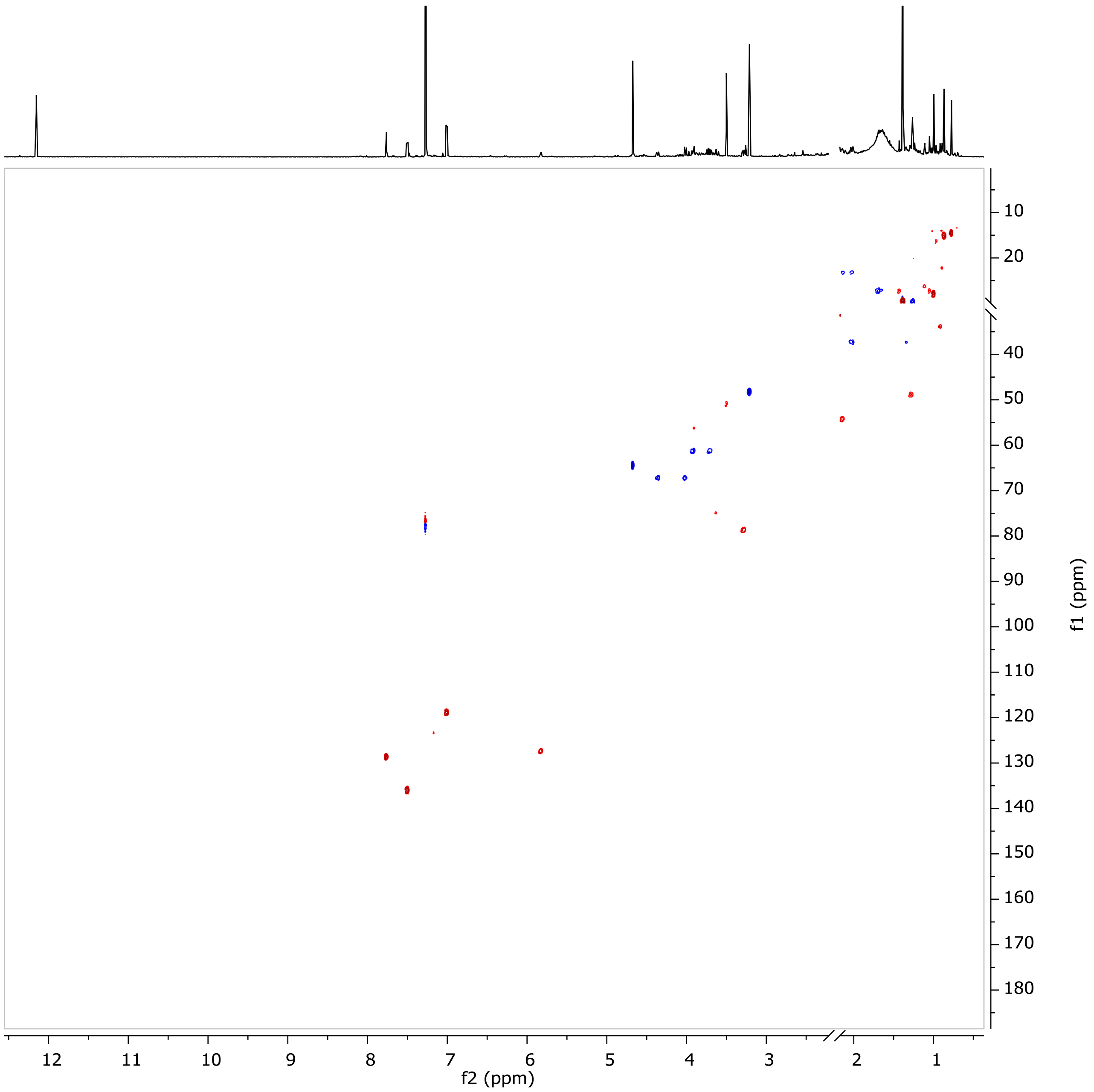

Figure S14. HSQC spectrum of **2** in methanol-d_4 at 500 MHz.

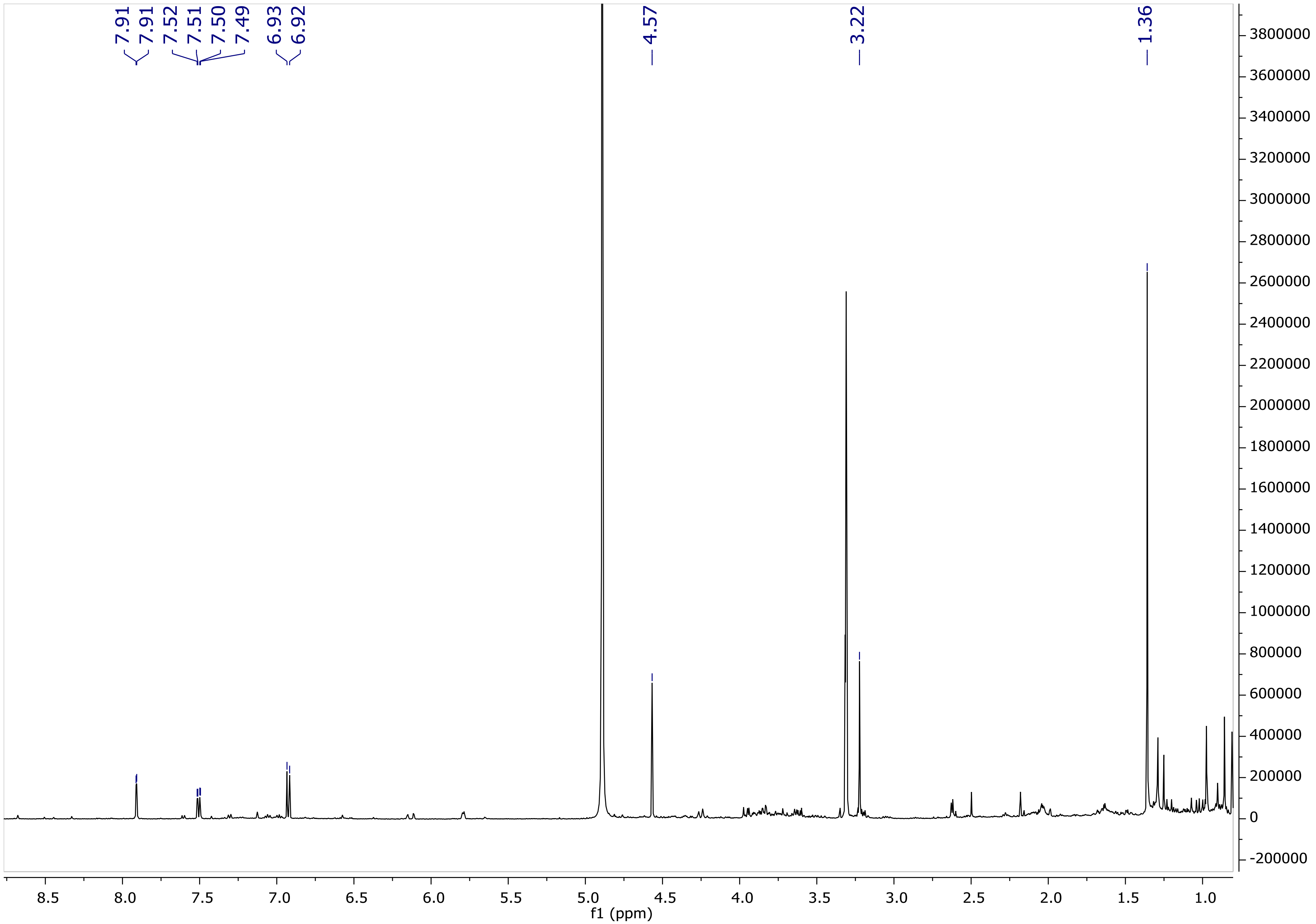

Figure S15. ^{1}H NMR spectrum of **2** in methanol-d_4 at 500 MHz.

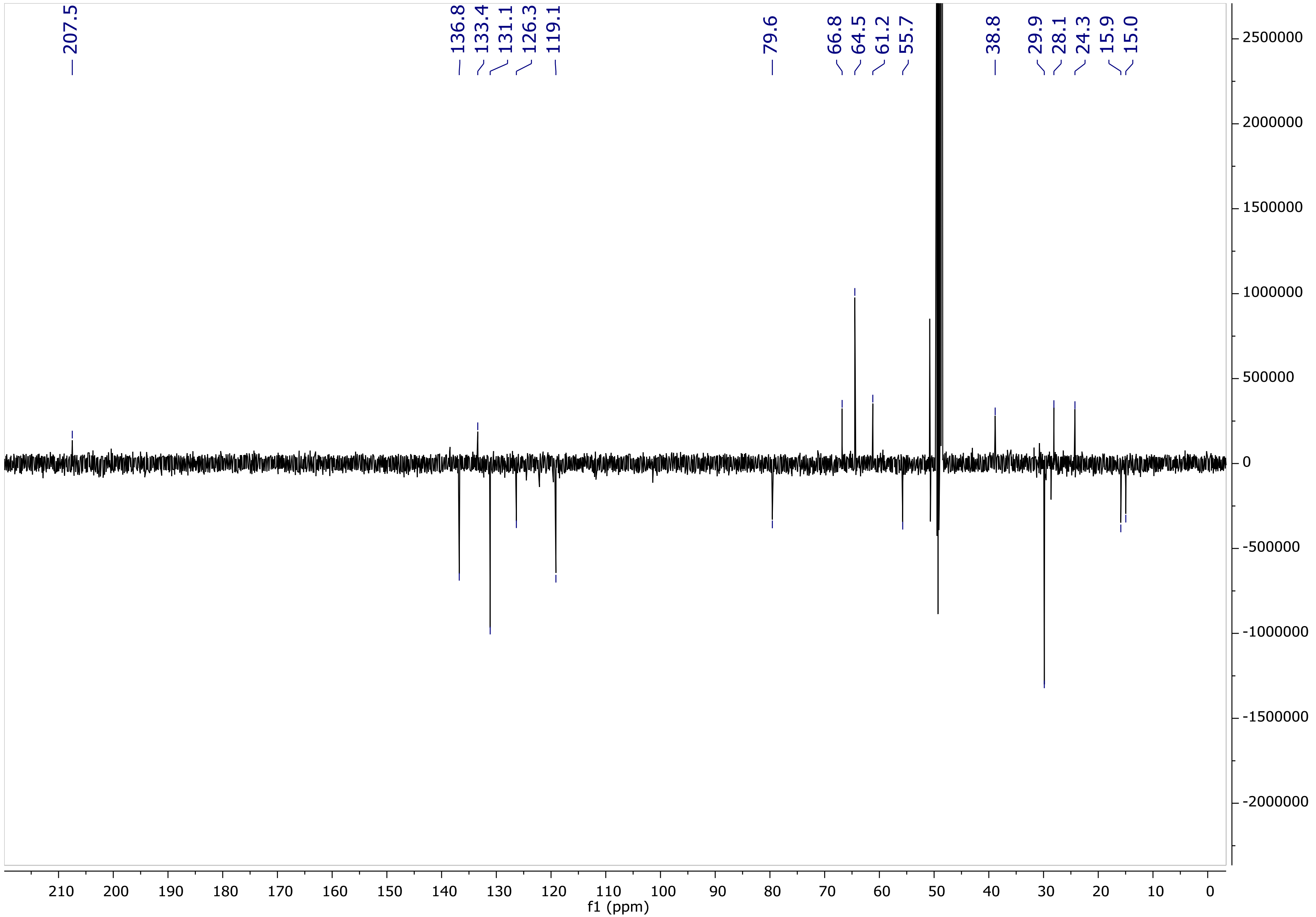

Figure S16. DEPTQ spectrum of **2** in methanol-d_4 at 125 MHz.

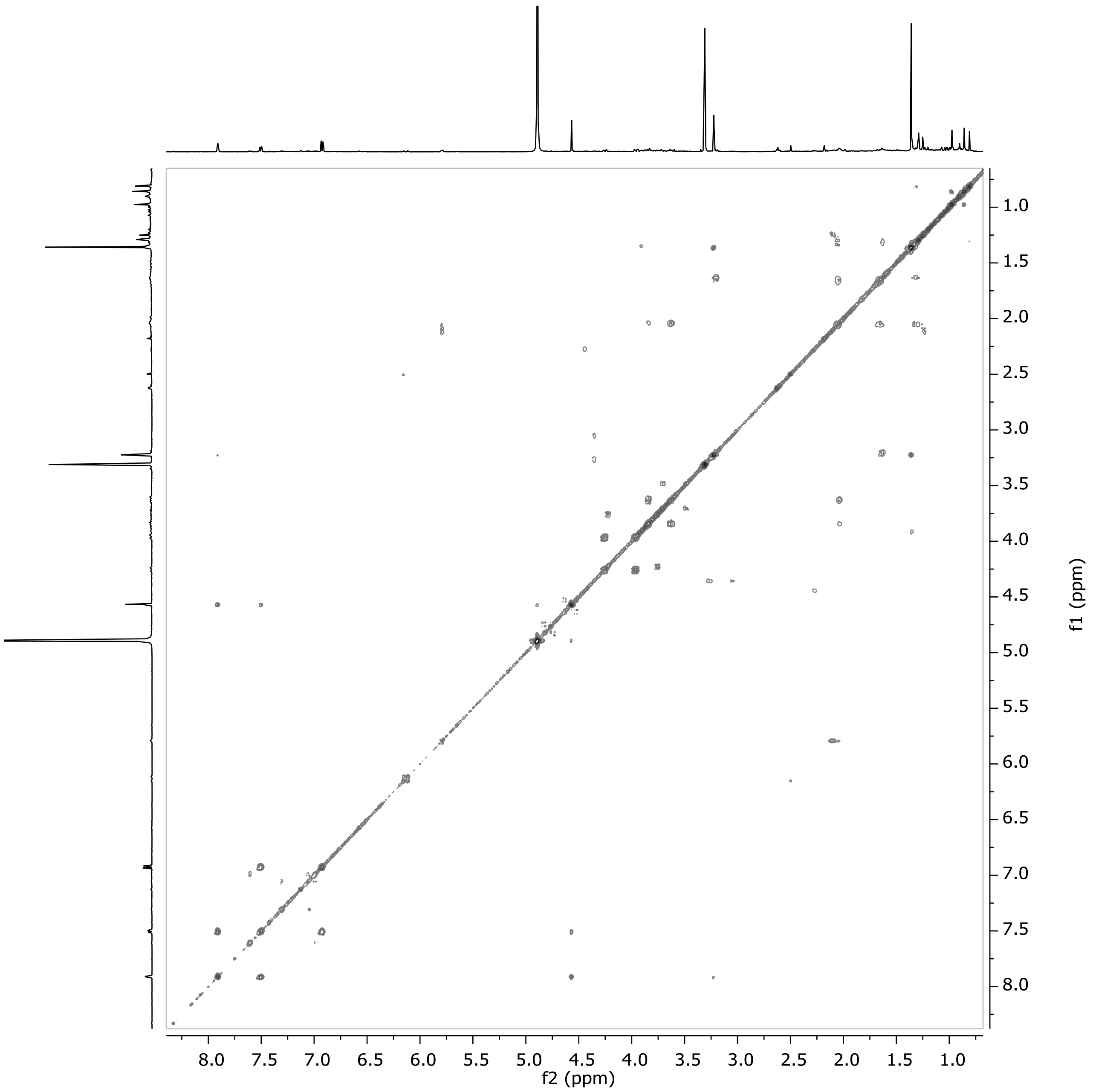

Figure S17. ^{1}H-^{1}H COSY spectrum of **2** in methanol-d_4 at 500 MHz.

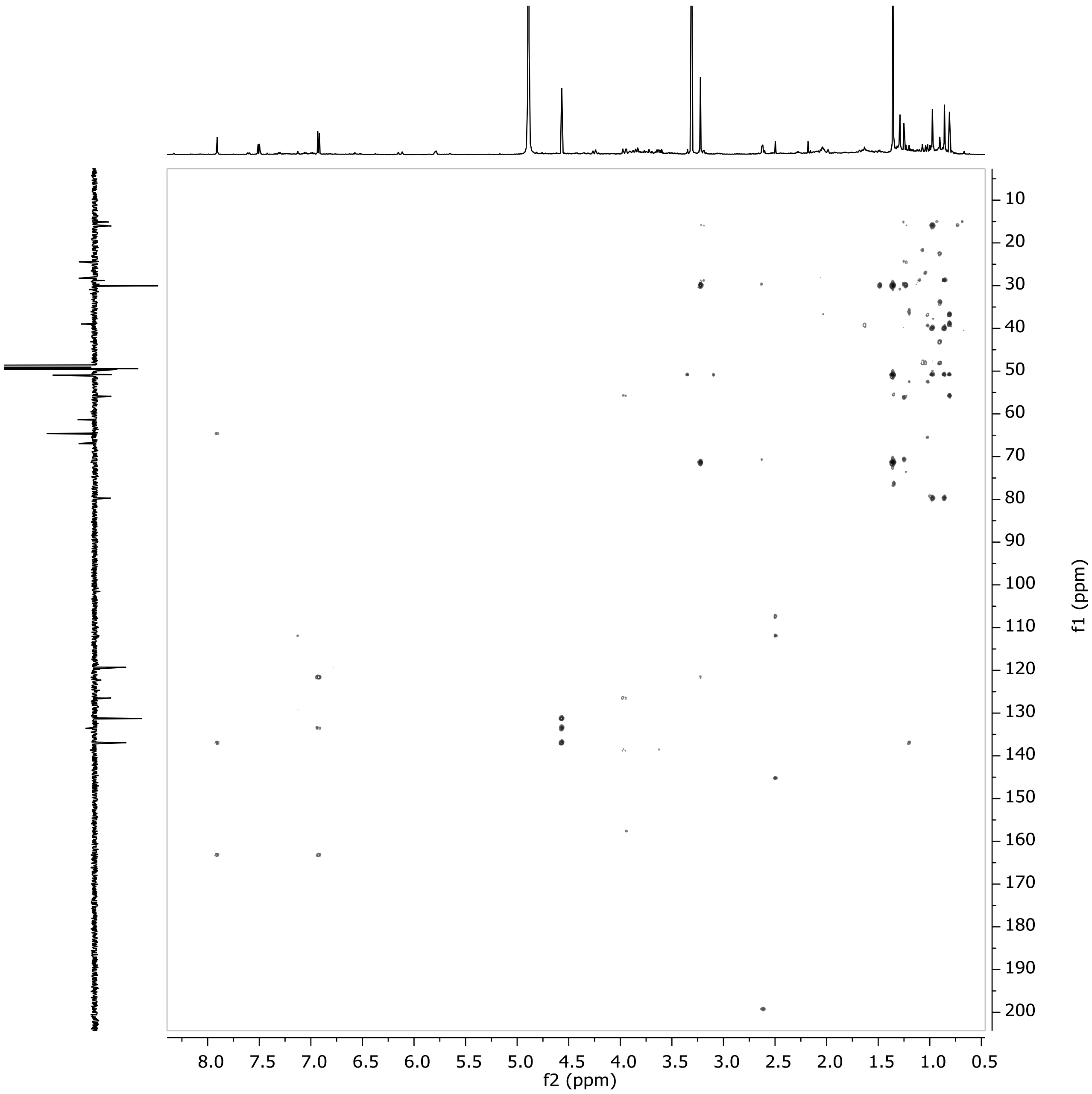

Figure S18. HMBC spectrum of **2** in methanol-d_4 at 500 MHz.

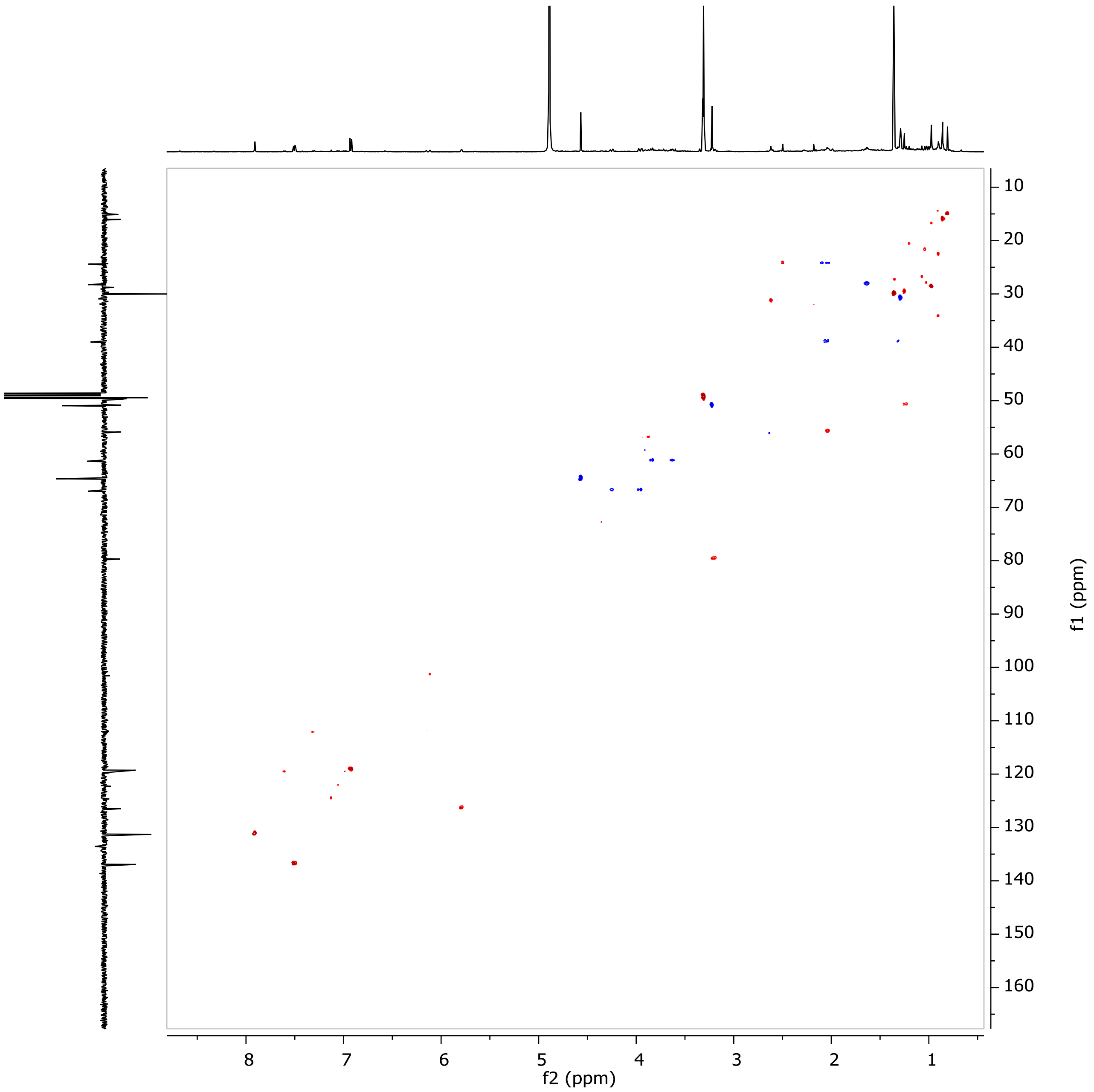

Figure S19. HSQC spectrum of **2** in methanol-d_4 at 500 MHz.

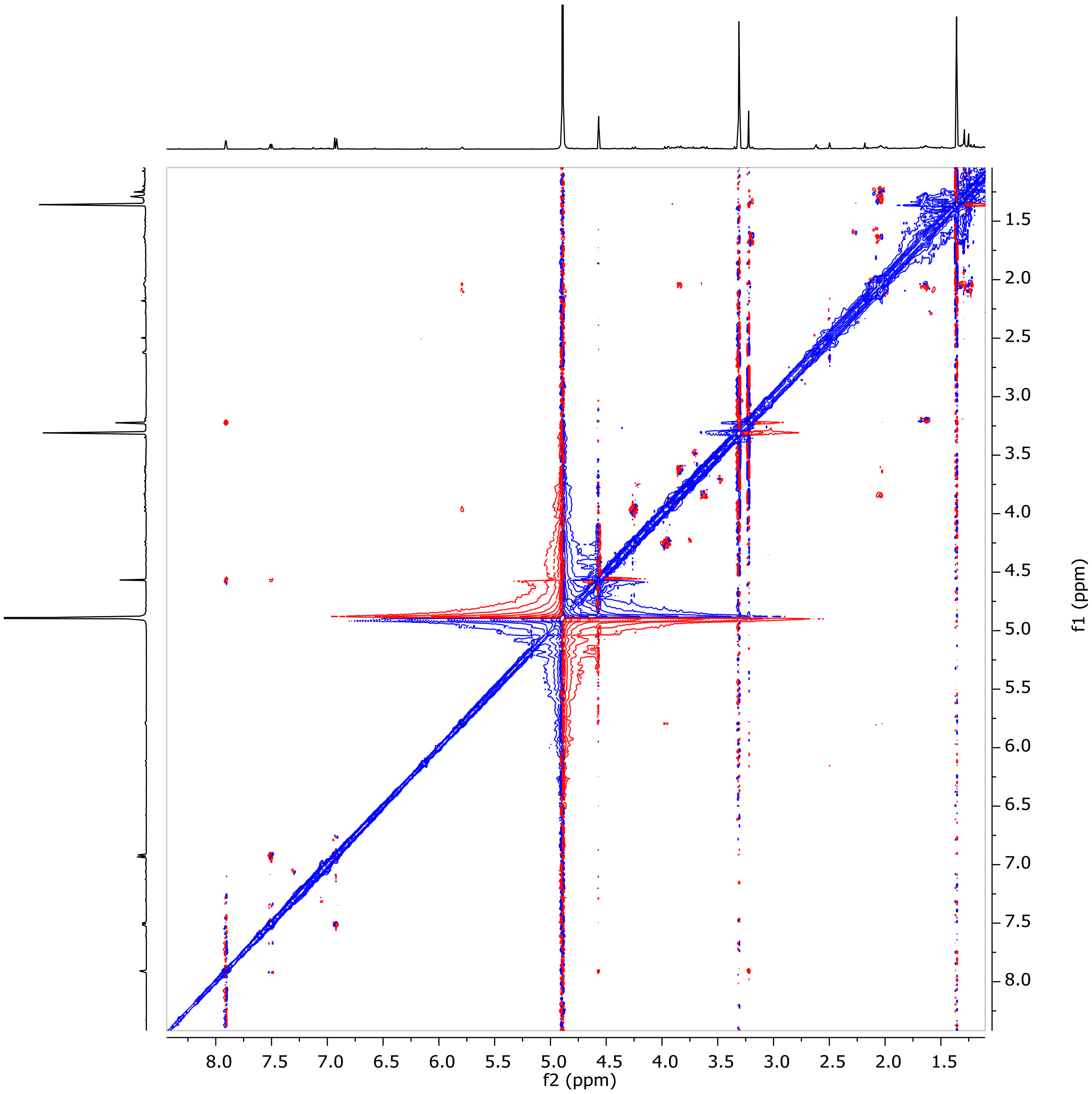

Figure S20. ROESY spectrum of **2** in methanol-d_4 at 500 MHz.

Display Report

Analysis Info

Analysis Name	F:\Volume D\HZI Projects\Winnie\11-Dentipellis fragilis\Amazon\Dent 6B_BA6_01_29108.d	Acquisition Date	28.05.2021 21:47:42
Method	29108.m	Operator	esu
Sample Name	Dent 6B	Instrument	amaZon speed
Comment			

Acquisition Parameter

Ion Source Type	ESI	Ion Polarity	Negative	Alternating Ion Polarity	on
Mass Range Mode	UltraScan	Scan Begin	100 m/z	Scan End	2000 m/z
Accumulation Time	4000 µs	RF Level	100 %	Trap Drive	71.3
SPS Target Mass	1000 m/z	Averages	6 Spectra		

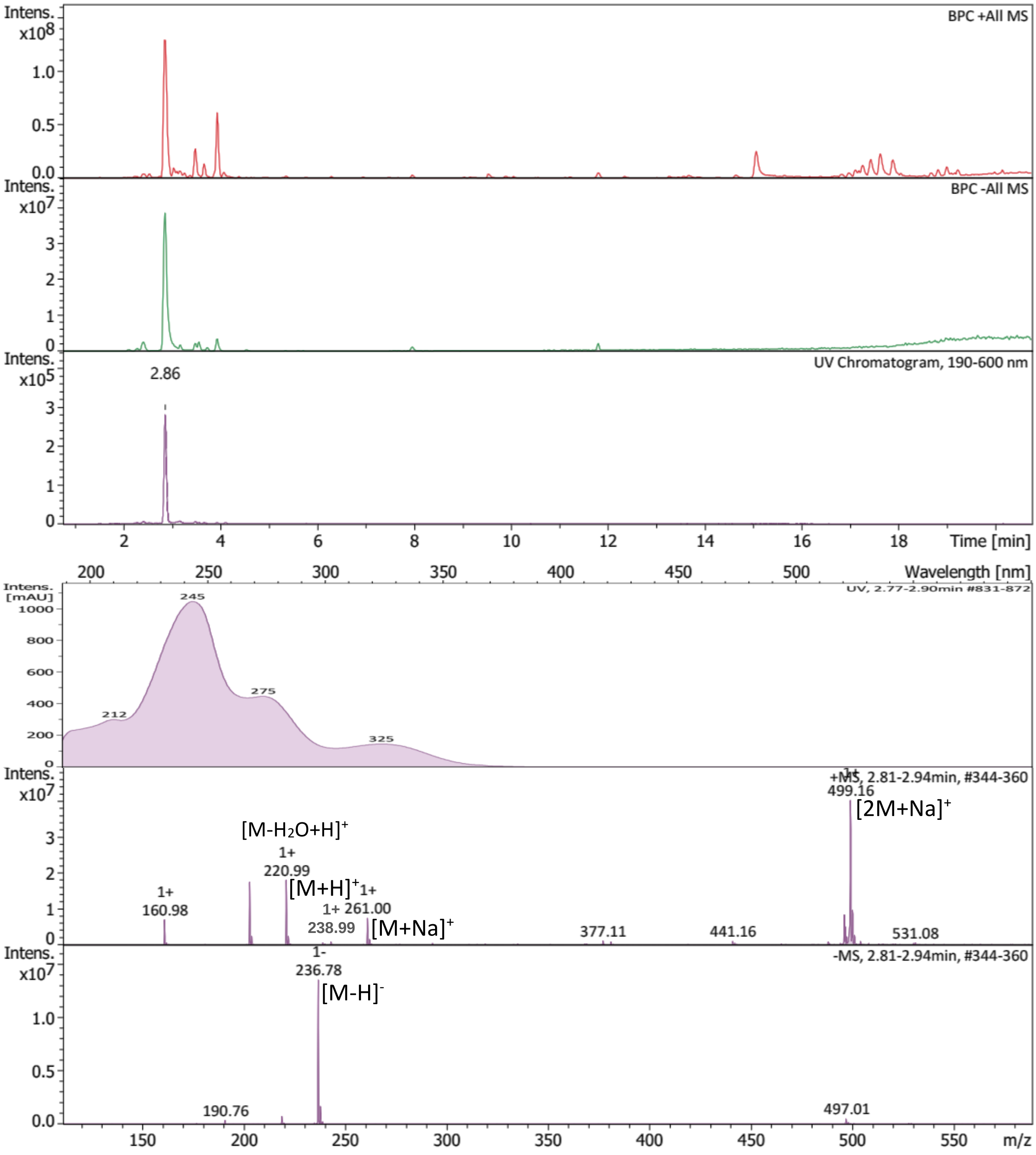

Bruker Compass DataAnalysis 6.1 printed: 10.04.2024 10:19:23 by: sel22 Page 1 of 1

Figure S21. LR-ESI-MS of **3**.

Display Report

Analysis Info

		Acquisition Date	02.06.2021 18:51:46
Analysis Name	F:\Volume D\HZI Projects\Winnie\11-Dentipellis fragilis\Maxis\Dent 6B_P1-C-2_01_7533.d		
Method	pos_säure_10000_screening_ms_100_2500_line.m	Operator	ate06
Sample Name	Dent 6B	Instrument	maXis
Comment	Screening01<br>Waters Acquity UPLC BEH C_18 1,7um 2.1x50mm		

Acquisition Parameter

Ion Polarity Positive

SPS Target Mass

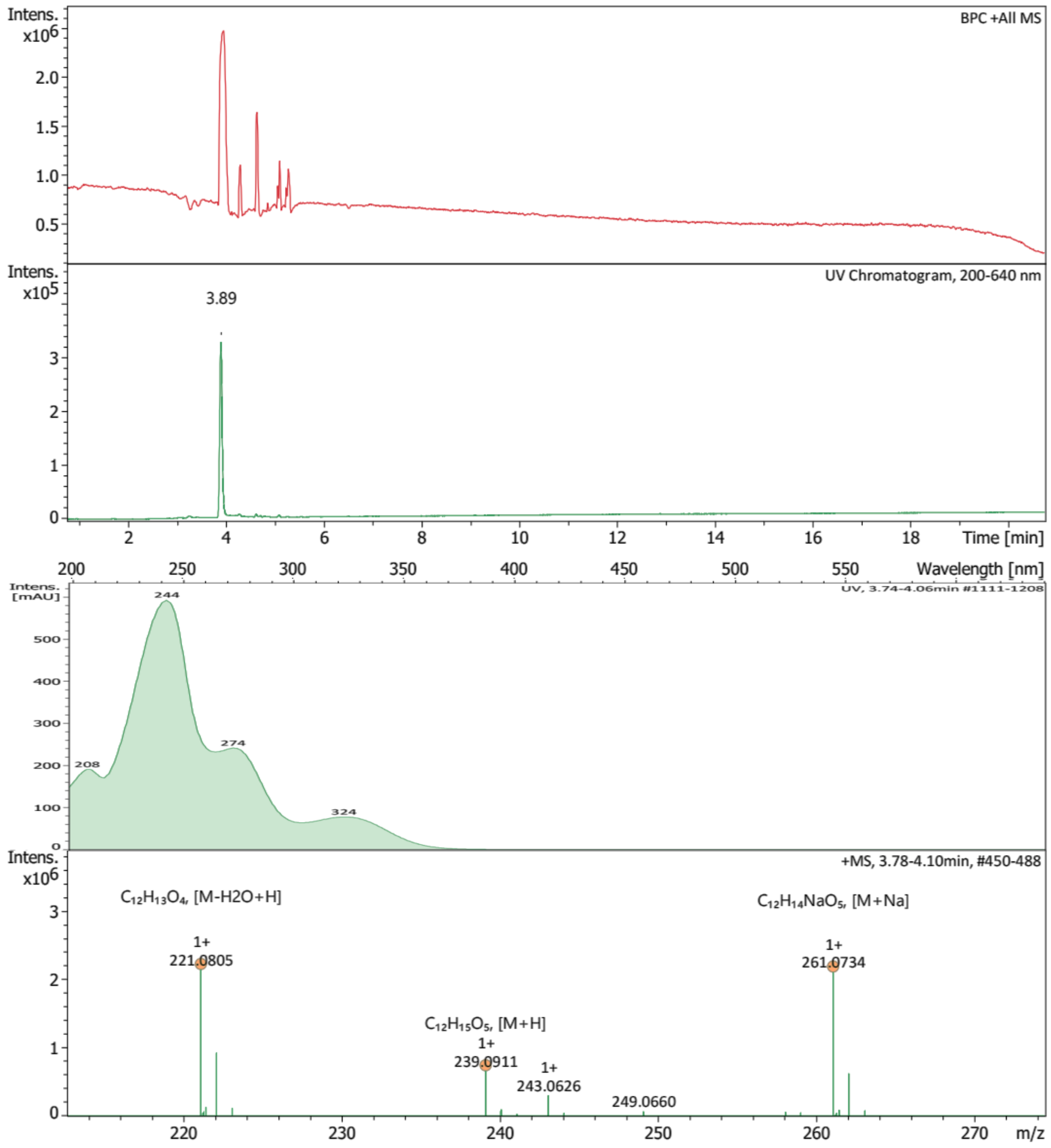

Bruker Compass DataAnalysis 6.1 printed: 10.04.2024 10:26:29 by: sel22 Page 1 of 1

Figure S22. HR-ESI-MS of **3**.

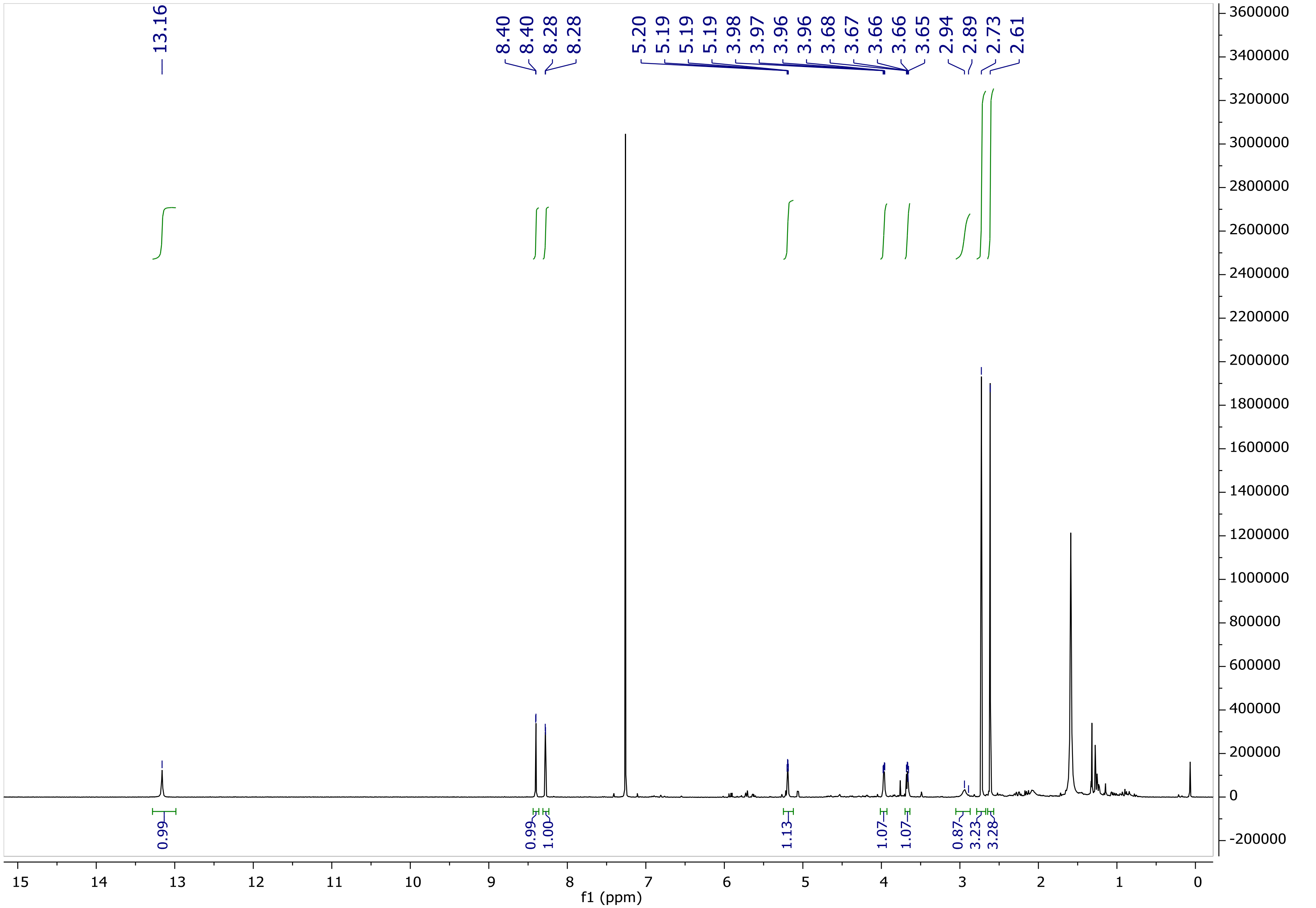

Figure S23. ^{1}H NMR spectrum of **3** in chloroform-*d* at 700 MHz.

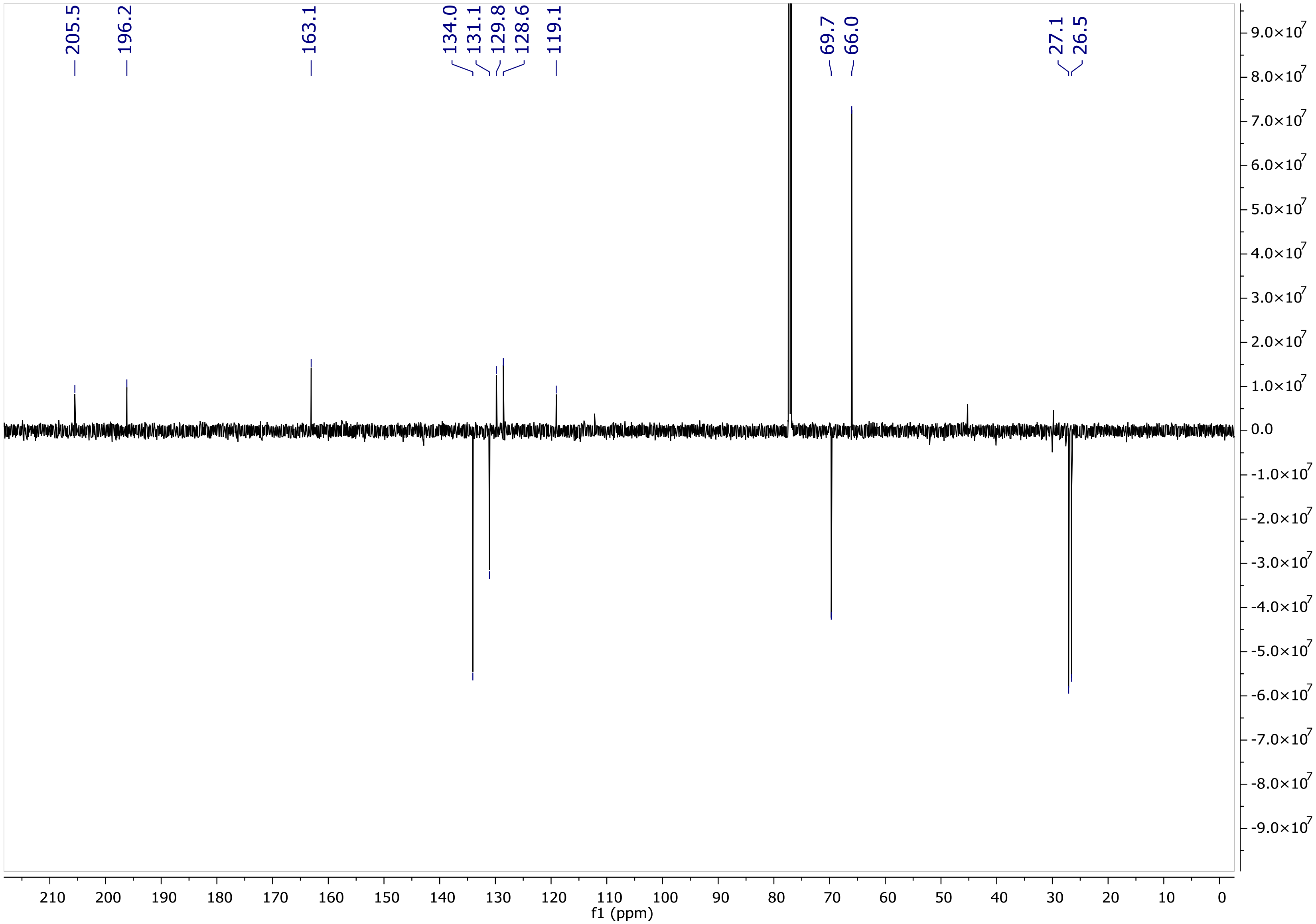

Figure S24. DEPTQ NMR spectrum of **3** in chloroform-*d* at 175 MHz.

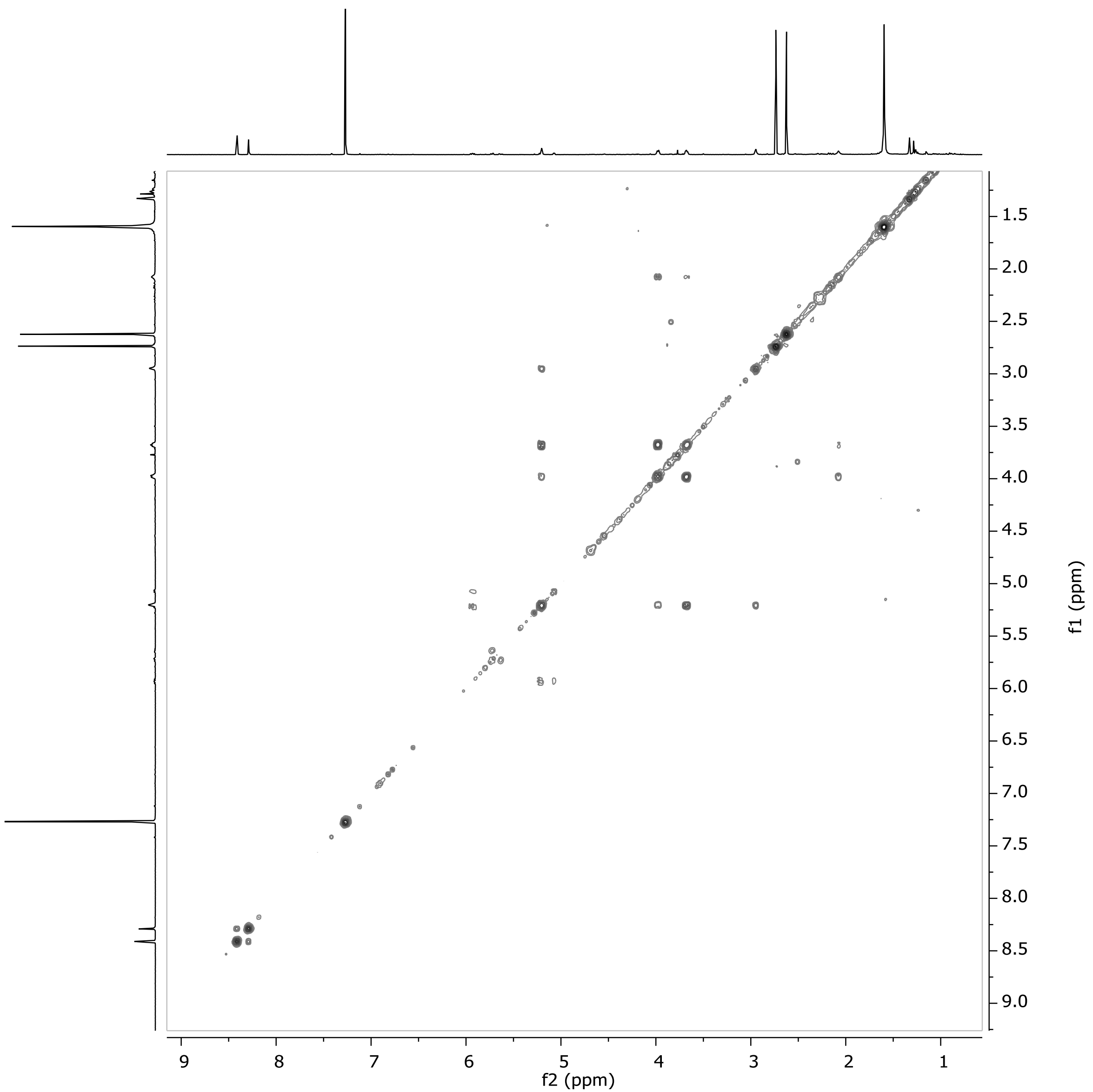

Figure S25. ^{1}H-^{1}H COSY spectrum of **3** in chloroform-*d* at 700 MHz.

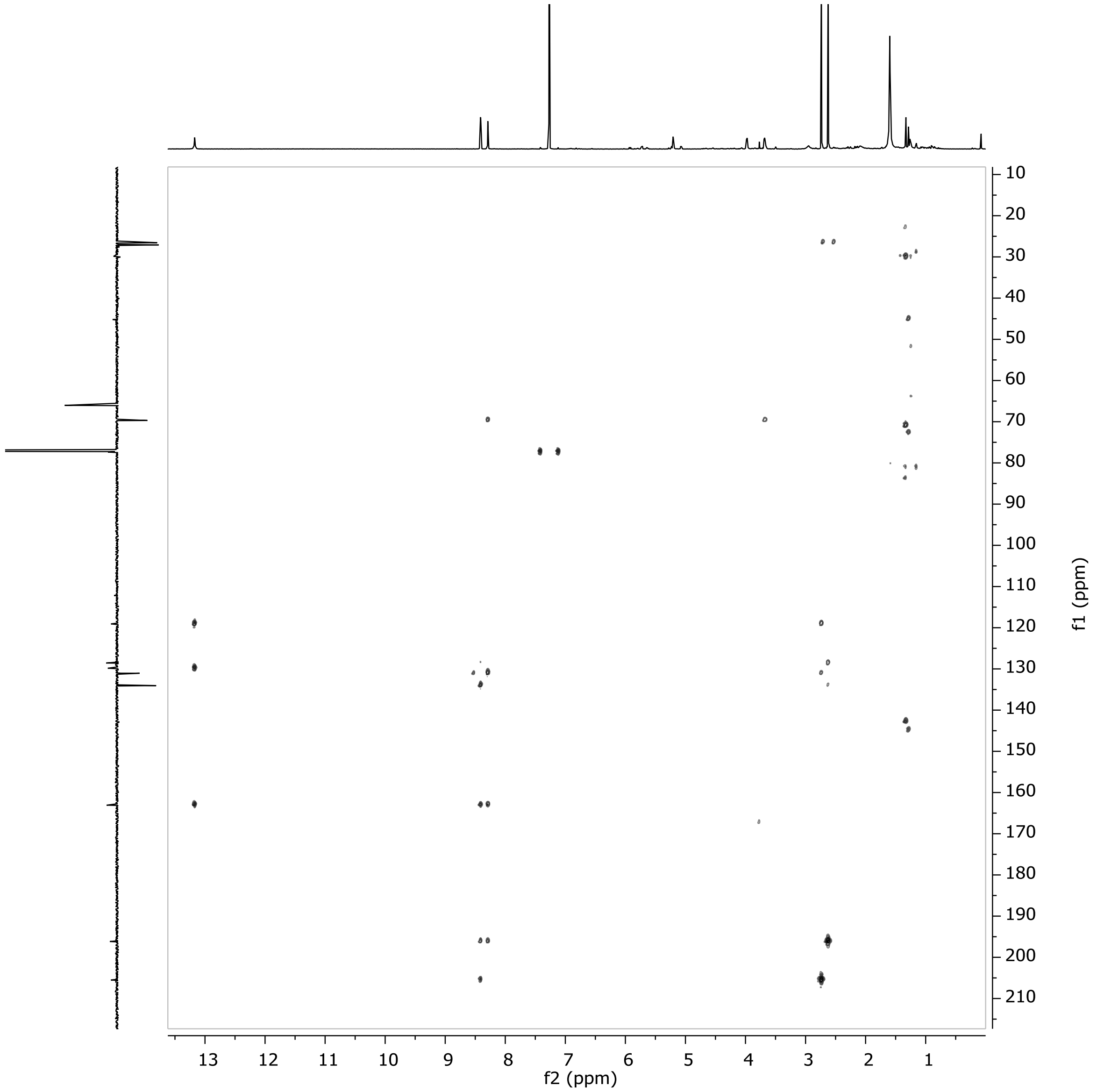

Figure S26. HMBC spectrum of **3** in chloroform-*d* at 700 MHz.

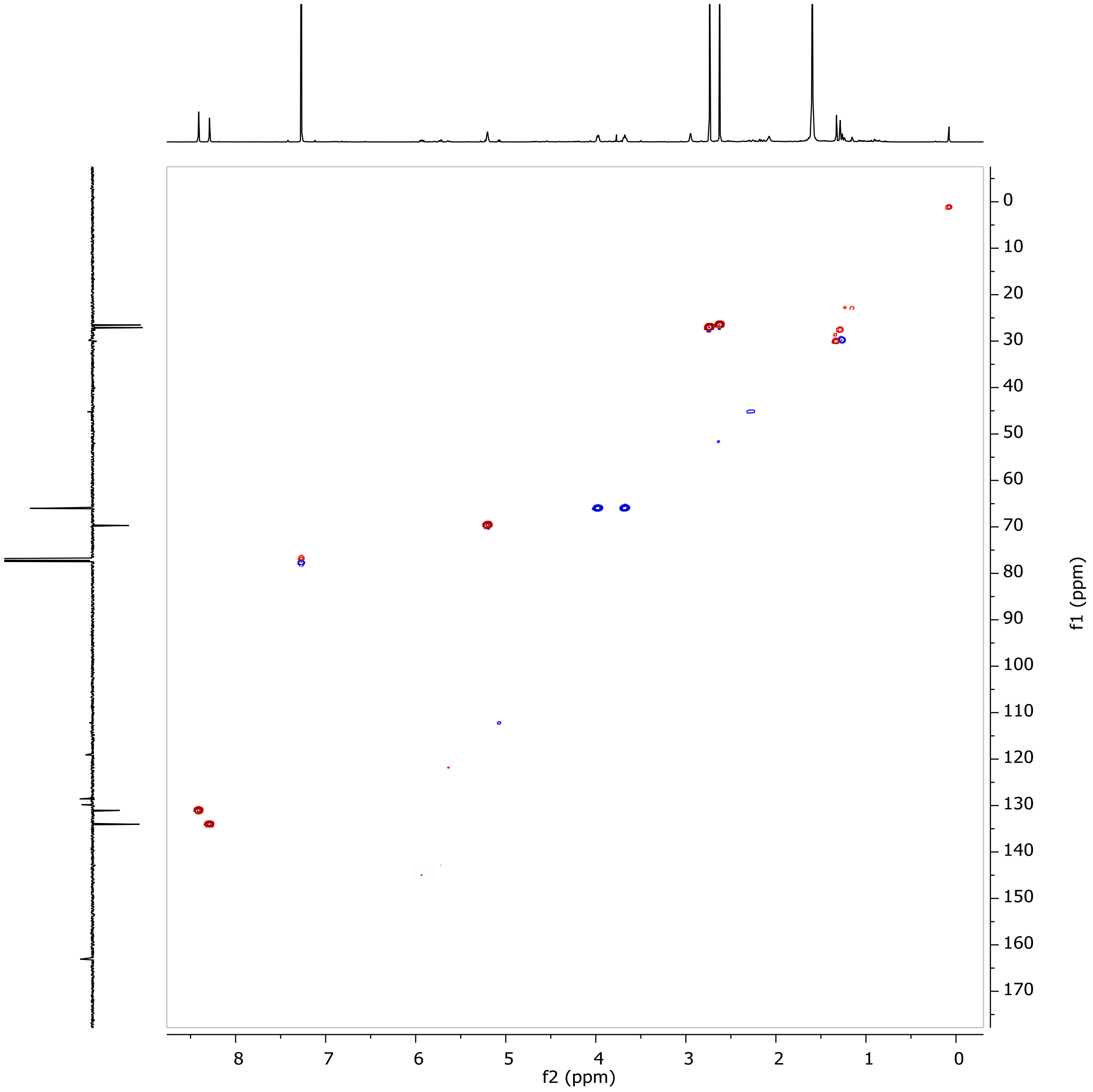

Figure S27. HSQC spectrum of **3** in chloroform-*d* at 700 MHz.

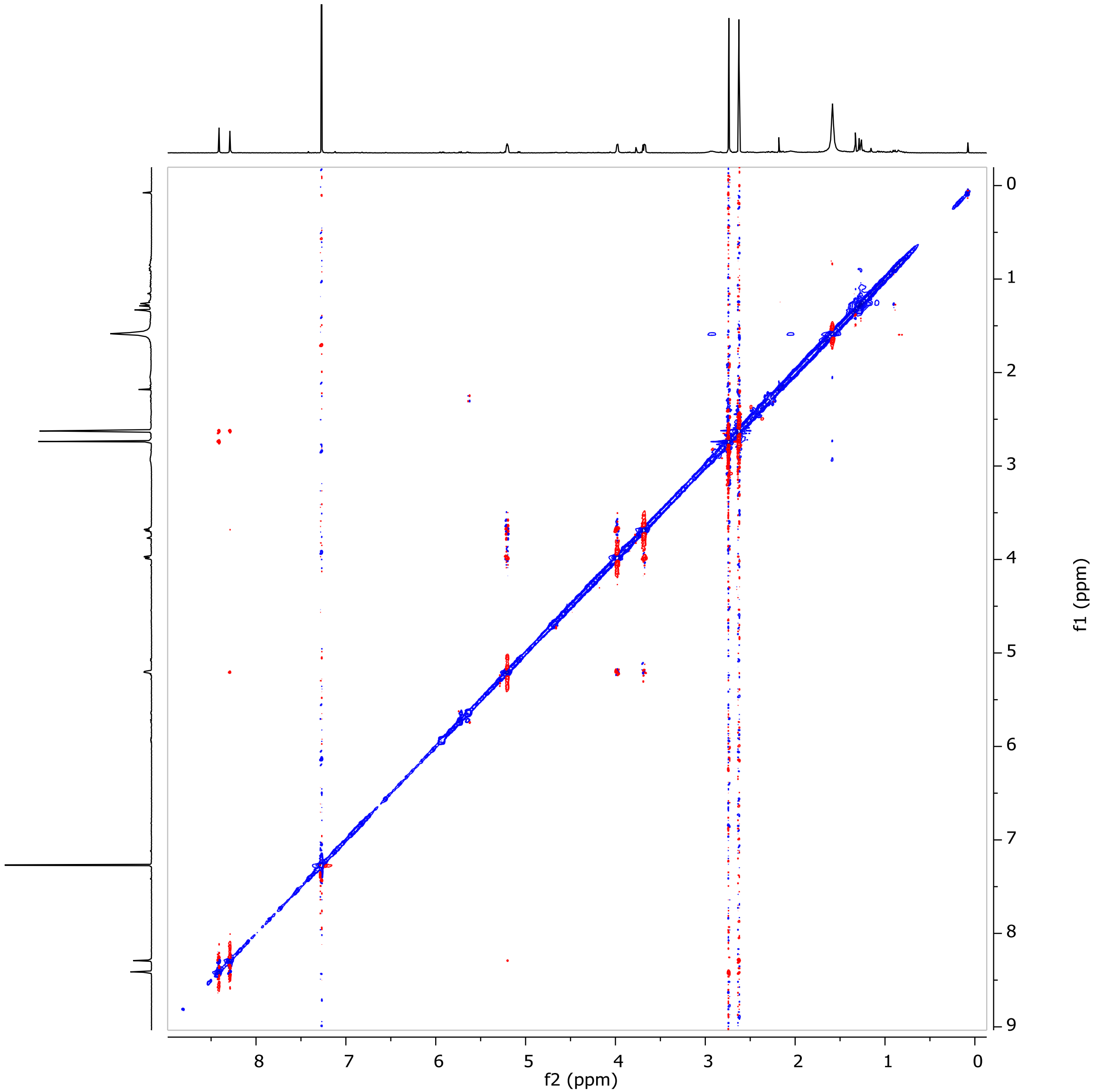

Figure S28. ROESY spectrum of **3** in chloroform-*d* at 700 MHz.

Table S1. 1D (^{1}H and ^{13}C) NMR data of **3** and dentipellinol.[1]

	**3**		Dentipellinol[1]	
**pos.**	**δ_C,^{a} type**	**δ_H^{b} (multi, *J*[Hz])**	**δ_C,^{c} type**	**δ_H^{d} (multi, *J*[Hz])**
1	163.1, C		163.9, C	
2	119.1, C		119.9, C	
3	131.1, CH	8.40 d (2.2)	132.5, CH	8.49 d (2.2)
4	128.6, C		129.4, C	
5	134.0, CH	8.28 d (2.2)	134.9, CH	8.28 d (2.2)
6	129.8, C		132.4, C	
7	205.5, CO		207.1, CO	
8	27.1, CH_3	2.73 s	27.0, CH_3	2.74 s
9	196.2, CO		198.7, CO	
10	26.5, CH_3	2.61 s	26.4, CH_3	2.61 s
11	69.7, CH	5.19 dd (7.2, 3.5)	69.6, CH	5.15 dd (6.8, 3.3)
12	66.0, CH_2	*α* 3.67 dd (11.2, 7.2)<br>*β* 3.97 dd (11.2, 3.5)	66.7, CH_2	*α* 3.55 dd (11.4, 6.8)<br>*β* 3.79 dd (11.4, 3.3)
1-OH	-	13.16 br s		

Measured in chloroform-*d* at ^{a} 175 / ^{b} 700 MHz.
Measured in methanol-d_4 at ^{c} 150 / ^{d} 600 MHz.
[1] Ki, D.-W.; Kim, C.-W.; Choi, D.-C.; Oh, G.-W.; Doan, T.-P.; Kim, J.-Y.; Oh, W.-K.; Lee, I.-K.; Yun, B.-S. Chemical constituents of the culture broth of *Dentipellis fragilis* and their anti-inflammatory activities. Phytochemistry 2023, 214, 113828.

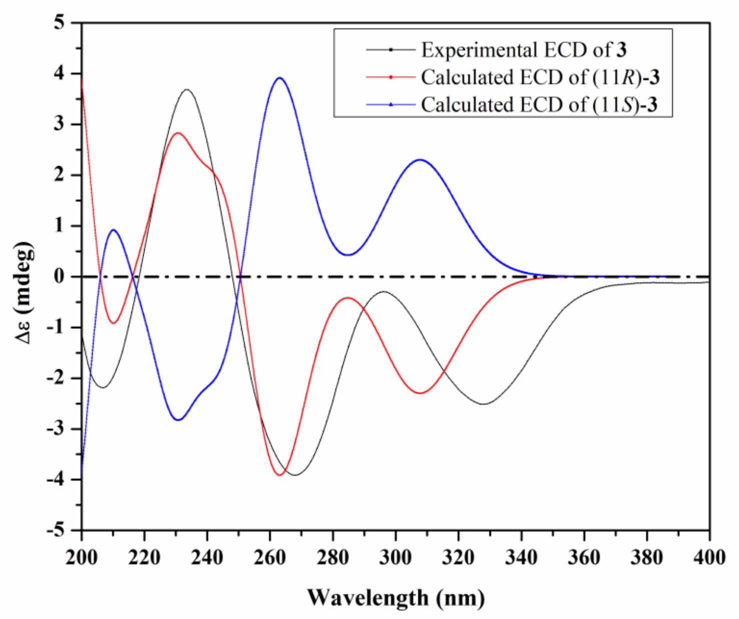

Figure S29. Experimental and calculated ECD spectra of **3**.

Display Report

Analysis Info

Analysis Name	F:\Volume D\HZI Projects\Winnie\11-Dentipellis fragilis\Amazon\Dent Rice F13_RC3_01_10235.d	Acquisition Date	18.02.2022 18:35:02
Method	Screening_Set-05iso-uv210_10235.m	Operator	lab
Sample Name	Dent Rice F13	Instrument	amaZon speed
Comment			

Acquisition Parameter

Ion Source Type	ESI	Ion Polarity	Positive	Alternating Ion Polarity	on
Mass Range Mode	UltraScan	Scan Begin	100 m/z	Scan End	2000 m/z
Accumulation Time	4000 µs	RF Level	100 %	Trap Drive	62.1
SPS Target Mass	1000 m/z	Averages	6 Spectra		

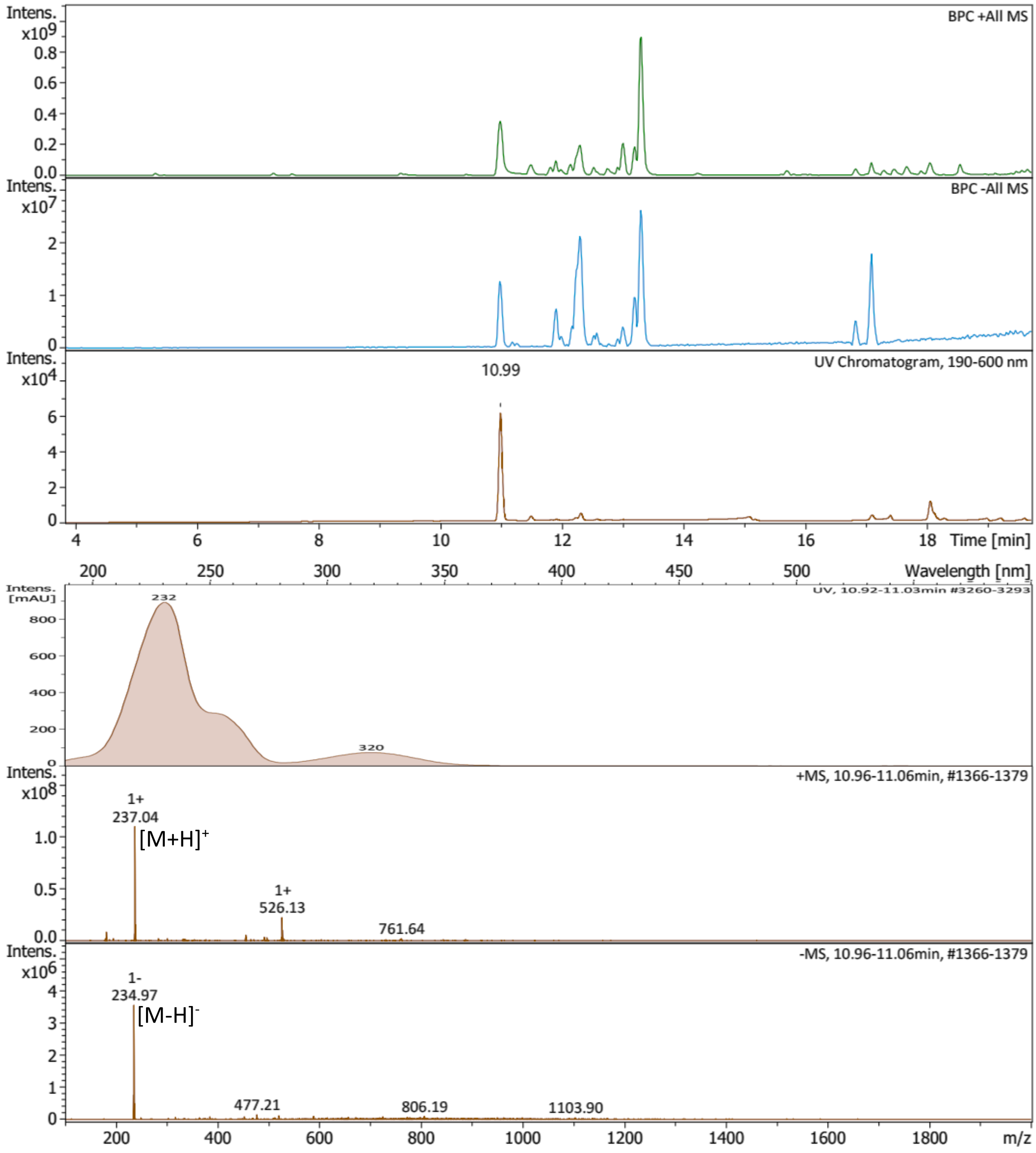

Bruker Compass DataAnalysis 6.1 printed: 10.04.2024 10:12:40 by: sel22 Page 1 of 1

Figure S30. LR-ESI-MS of **4**.

Display Report

Analysis Info

Analysis Name	S:\DATA\MaXis\wsu20_Winnie Sum Chemutai\22_02\Dent Rice F13_P1-B-4_01_9611.d	Acquisition Date	23.02.2022 13:09:09
Method	pos_säure_10000_screening_ms_100_2500_line.m	Operator	ate06
Sample Name	Dent Rice F13	Instrument	maXis 255552.00037
Comment	Screening01<br>Waters Acquity UPLC BEH C_18 1,7um 2.1x50mm		

Acquisition Parameter

Source Type	ESI	Ion Polarity	Positive	Set Nebulizer	4.0 Bar
Focus	Not active	Set Capillary	4500 V	Set Dry Heater	200 °C
Scan Begin	50 m/z	Set End Plate Offset	-500 V	Set Dry Gas	10.0 l/min
Scan End	2500 m/z	Set Charging Voltage	0 V	Set Divert Valve	Waste
		Set Corona	0 nA	Set APCI Heater	0 °C

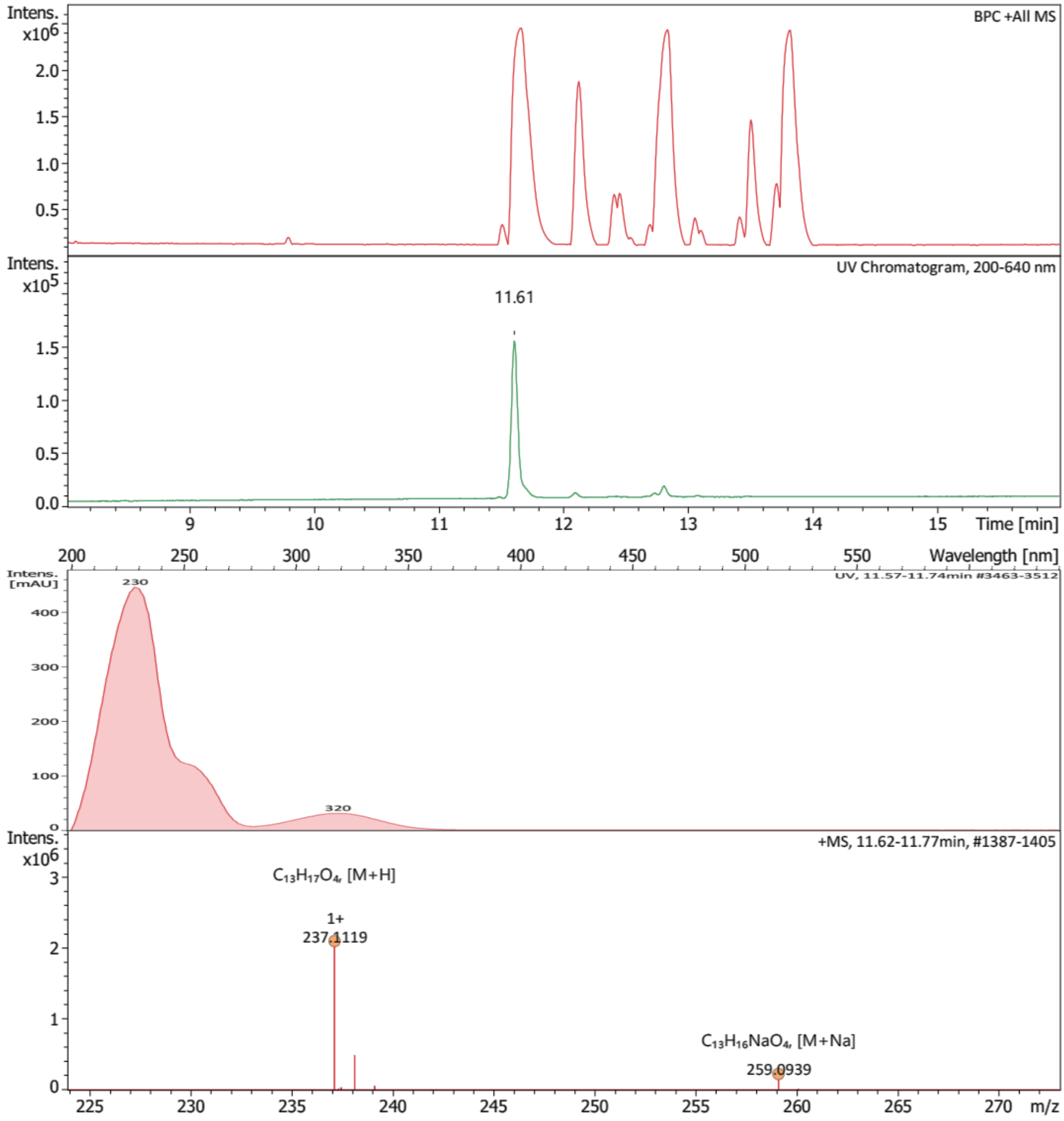

Dent Rice F13_P1-B-4_01_9611.d

Bruker Compass DataAnalysis 6.1 printed: 05.04.2024 16:16:50 by: sel22 Page 1 of 1

Figure S31. HR-ESI-MS of **4**.

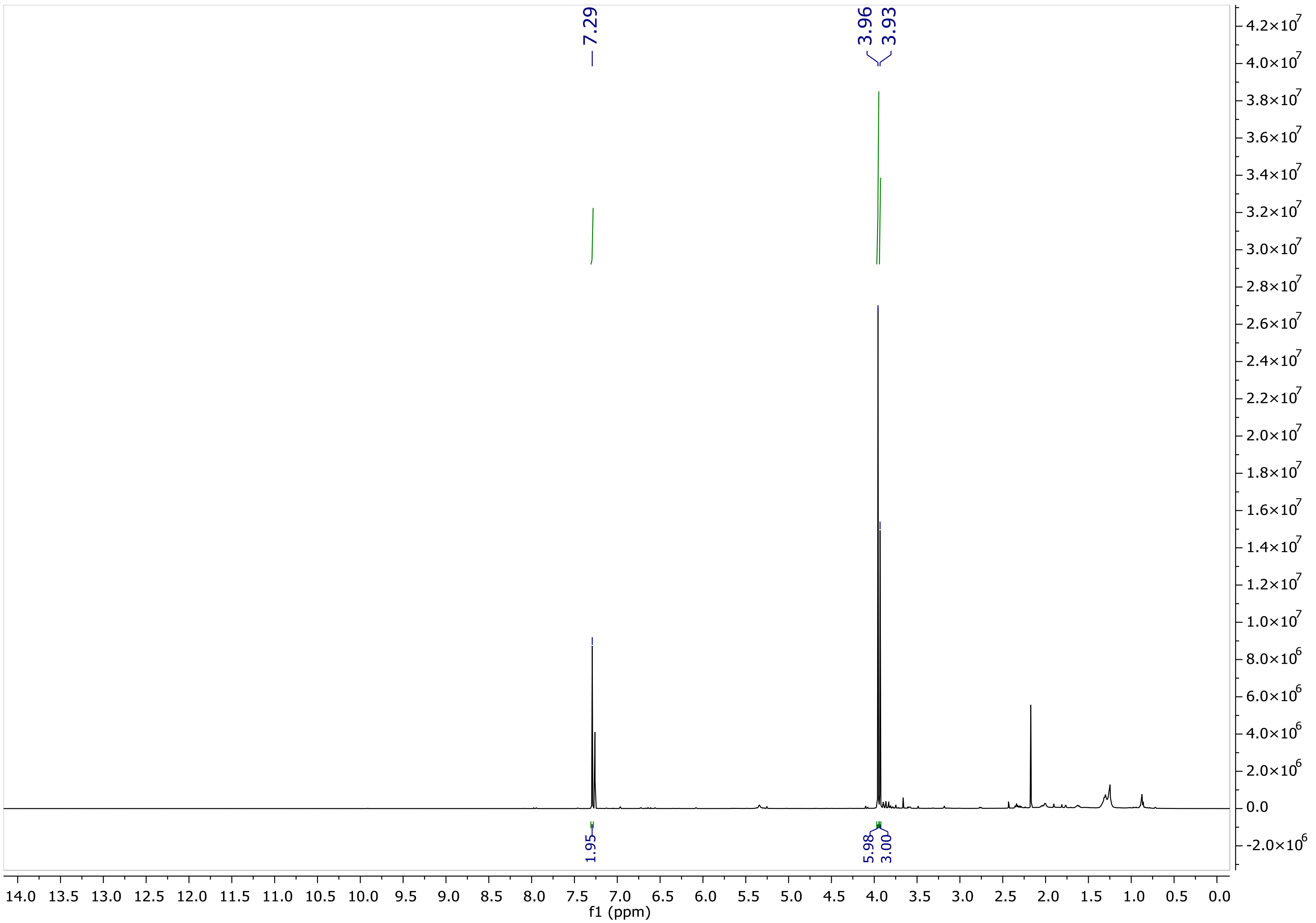

Figure S32. ^{1}H NMR spectrum of **4** in chloroform-*d* at 500 MHz.

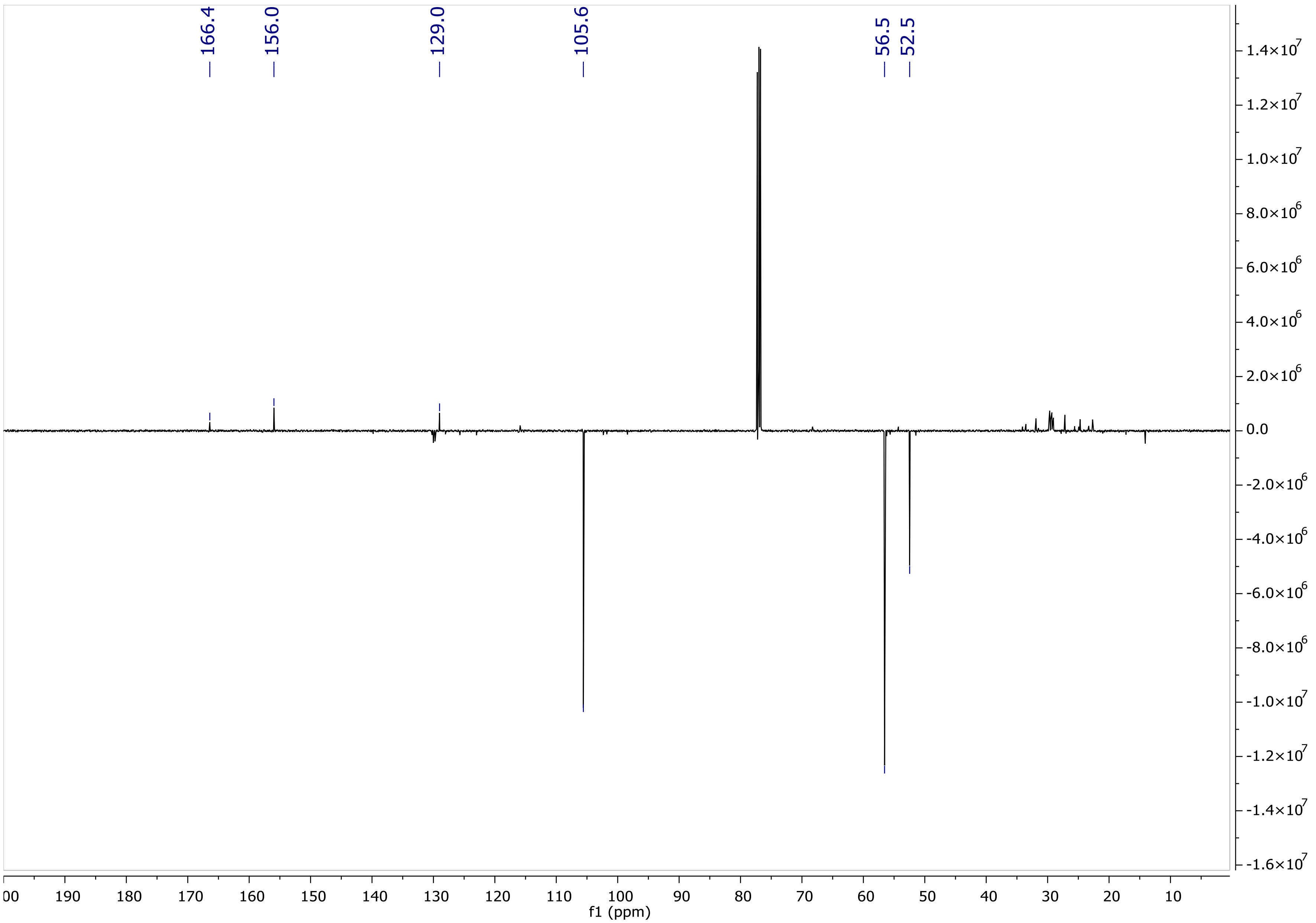

Figure S33. DEPTQ spectrum of **4** in chloroform-*d* at 125 MHz.

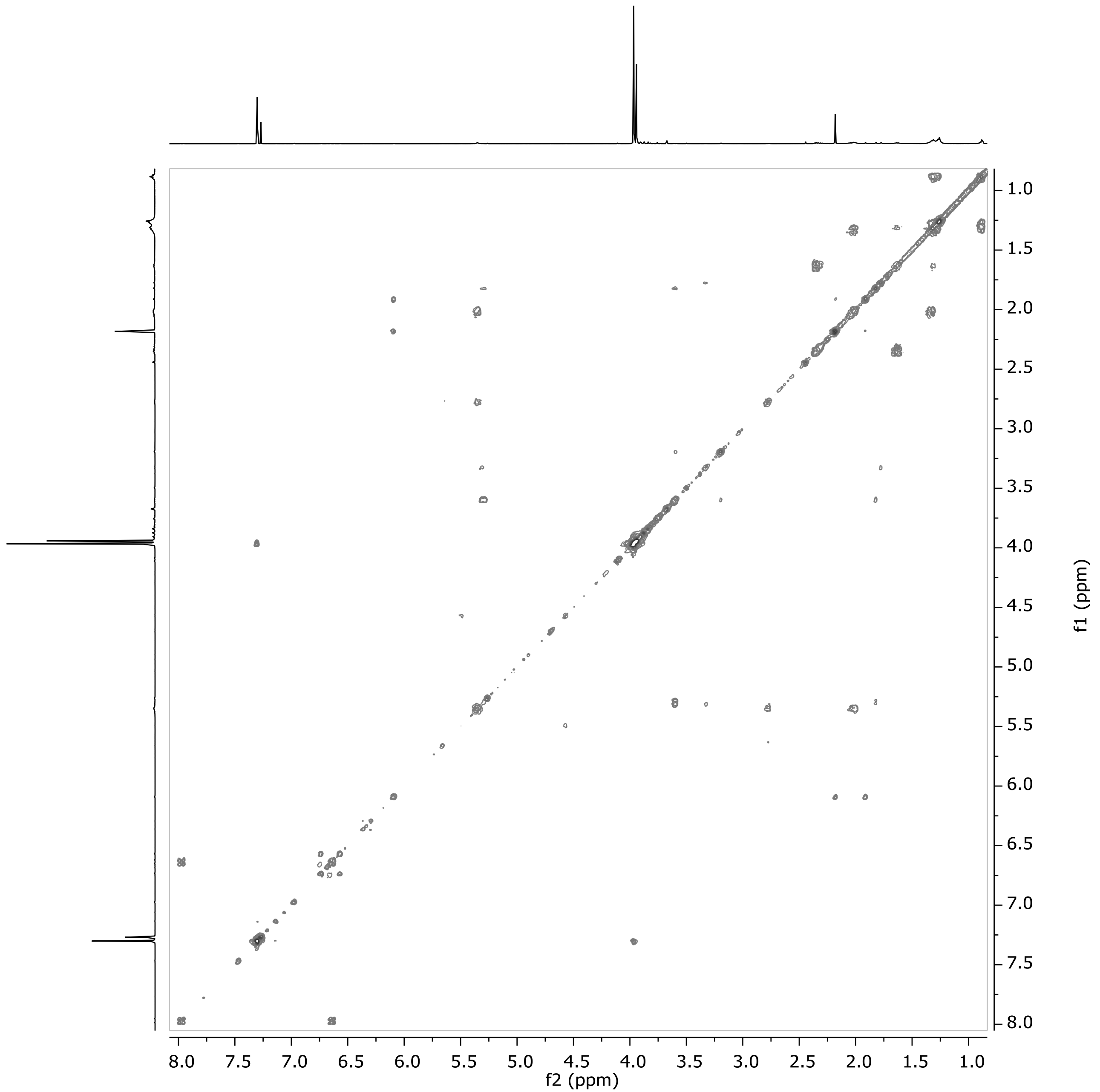

Figure S34. ^{1}H-^{1}H COSY spectrum of **4** in chloroform-*d* at 500 MHz.

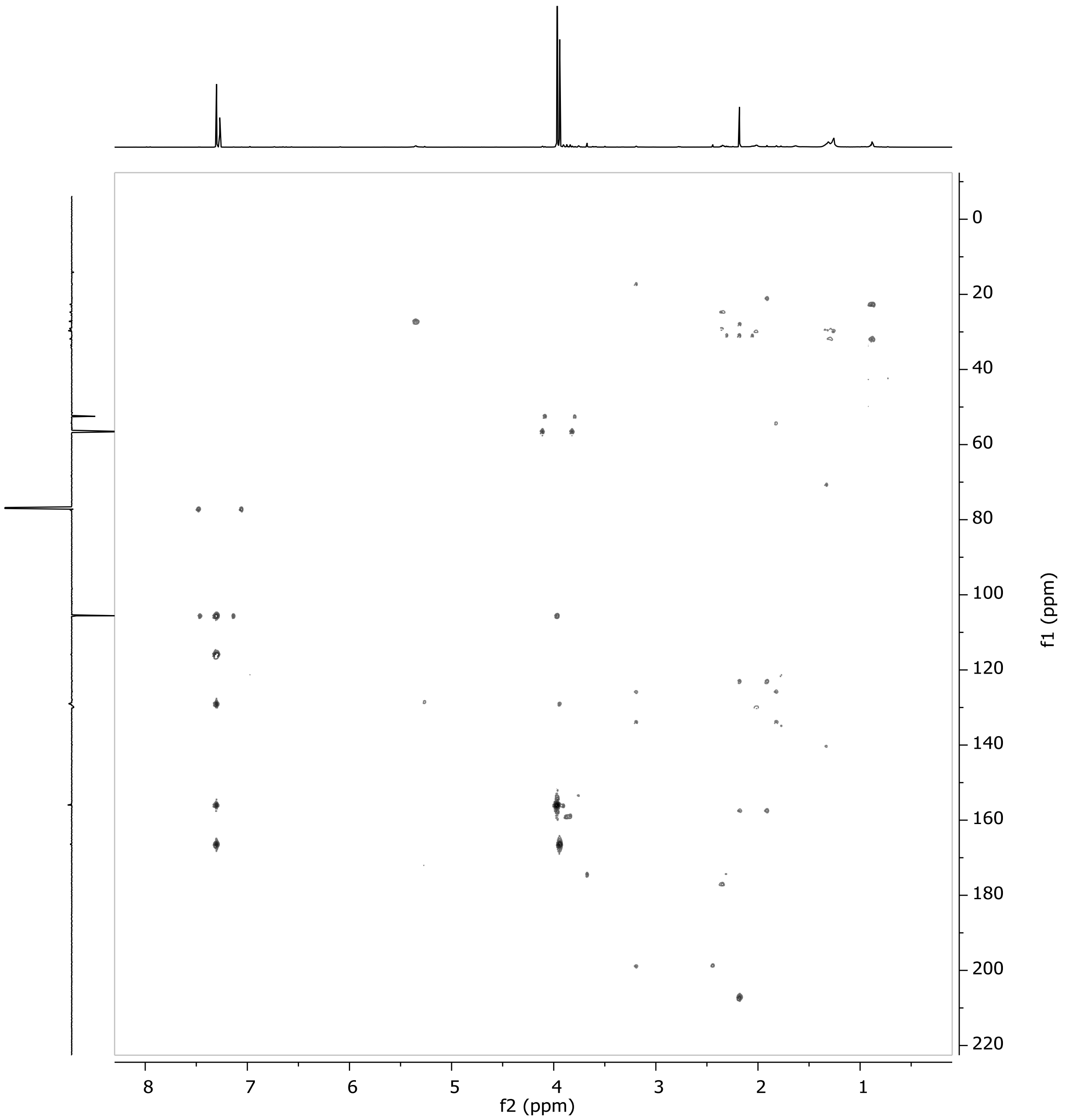

Figure S35. HMBC spectrum of **4** in chloroform-*d* at 500 MHz.

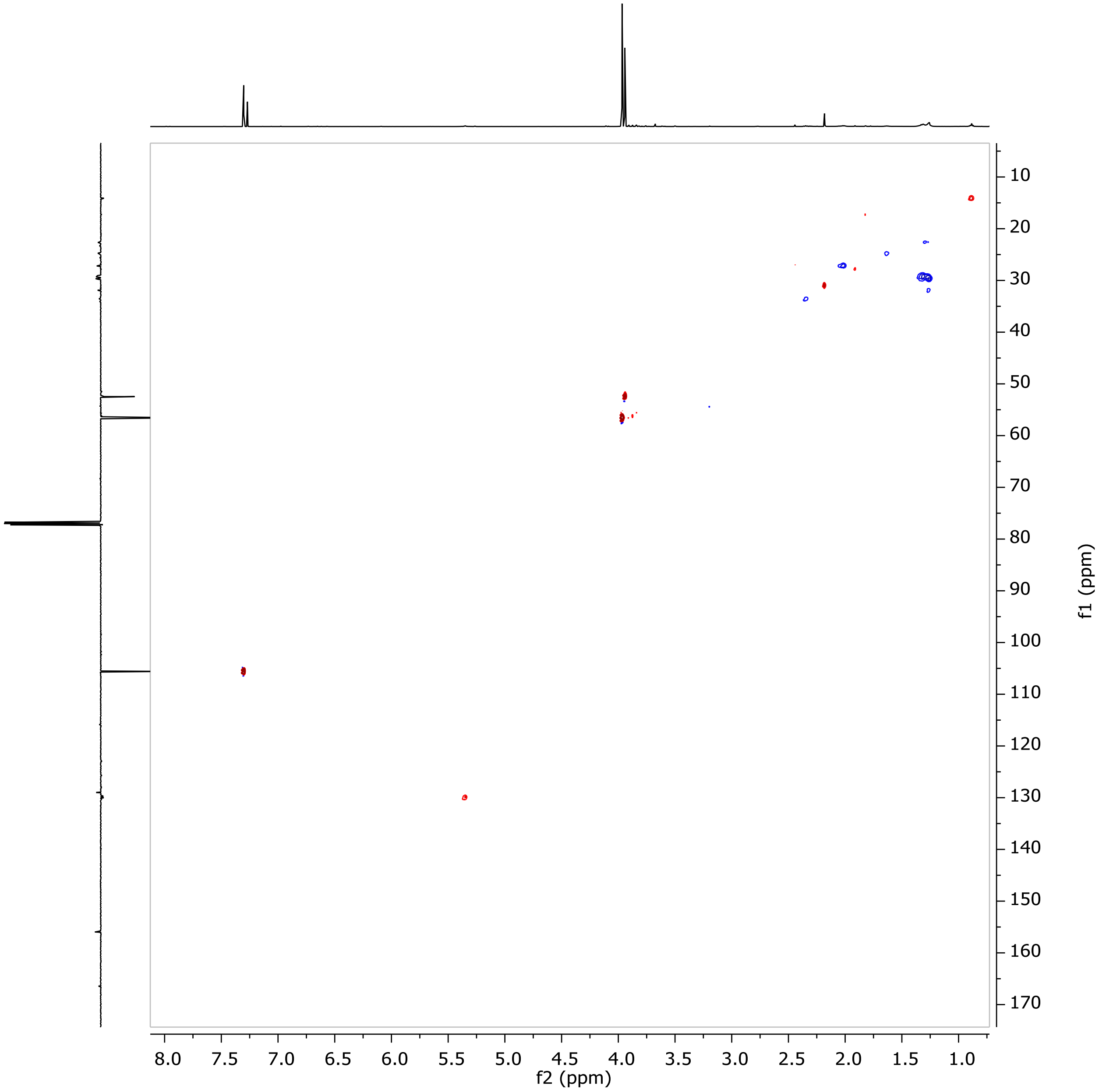

Figure S36. HSQC spectrum of **4** in chloroform-*d* at 500 MHz.

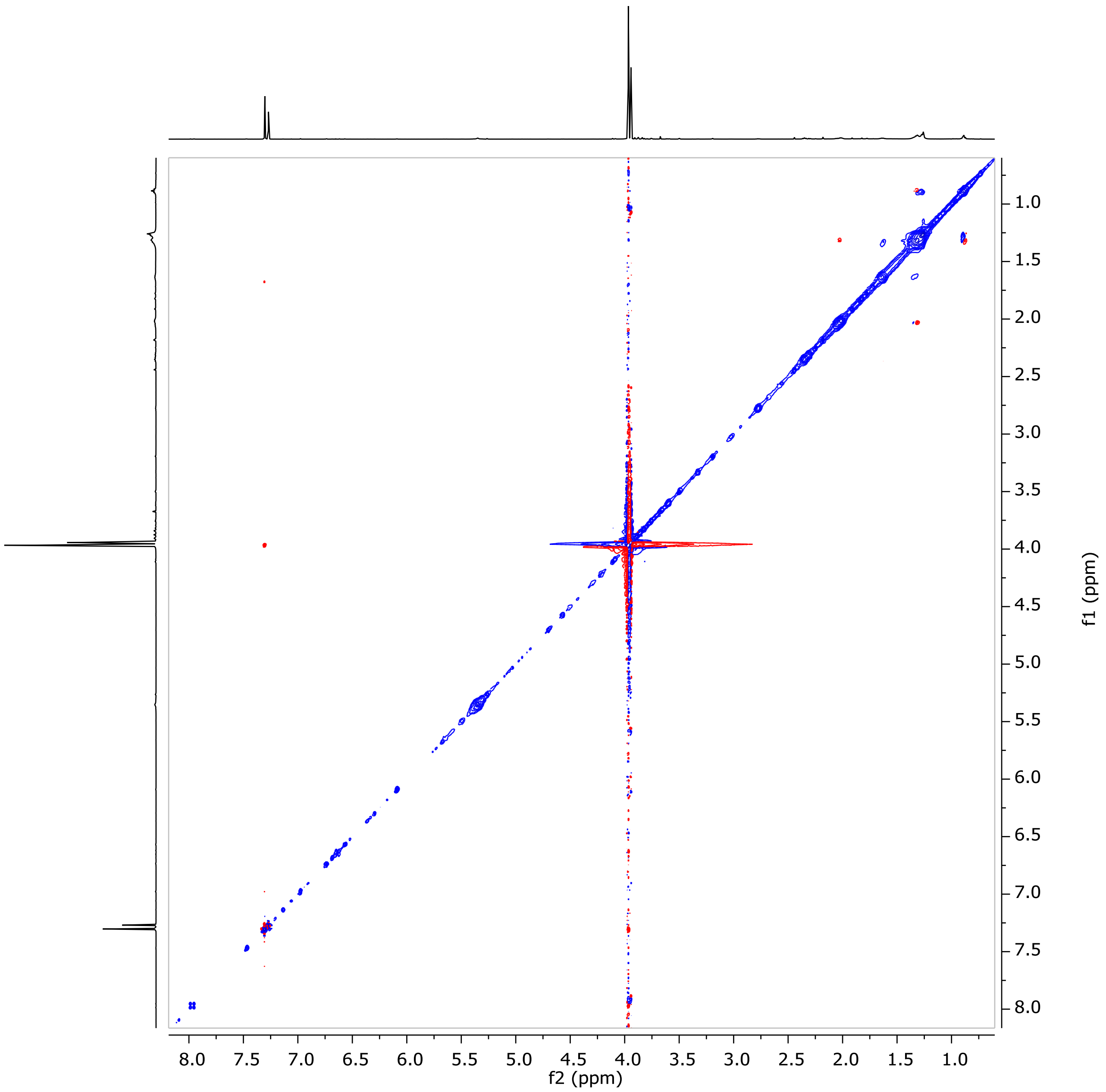

Figure S37. ROESY spectrum of **4** in chloroform-*d* at 500 MHz.

Display Report

Analysis Info

		Acquisition Date	18.02.2022 19:47:25
Analysis Name	F:\Volume D\HZI Projects\Winnie\11-Dentipellis fragilis\Amazon\Dent Rice F15_RC5_01_10237.d		
Method	Screening_Set-05iso-uv210_10237.m	Operator	lab
Sample Name	Dent Rice F15	Instrument	amaZon speed
Comment			

Acquisition Parameter

Ion Source Type	ESI	Ion Polarity	Negative	Alternating Ion Polarity	on
Mass Range Mode	UltraScan	Scan Begin	100 m/z	Scan End	2000 m/z
Accumulation Time	4000 µs	RF Level	100 %	Trap Drive	68.9
SPS Target Mass	1000 m/z	Averages	6 Spectra		

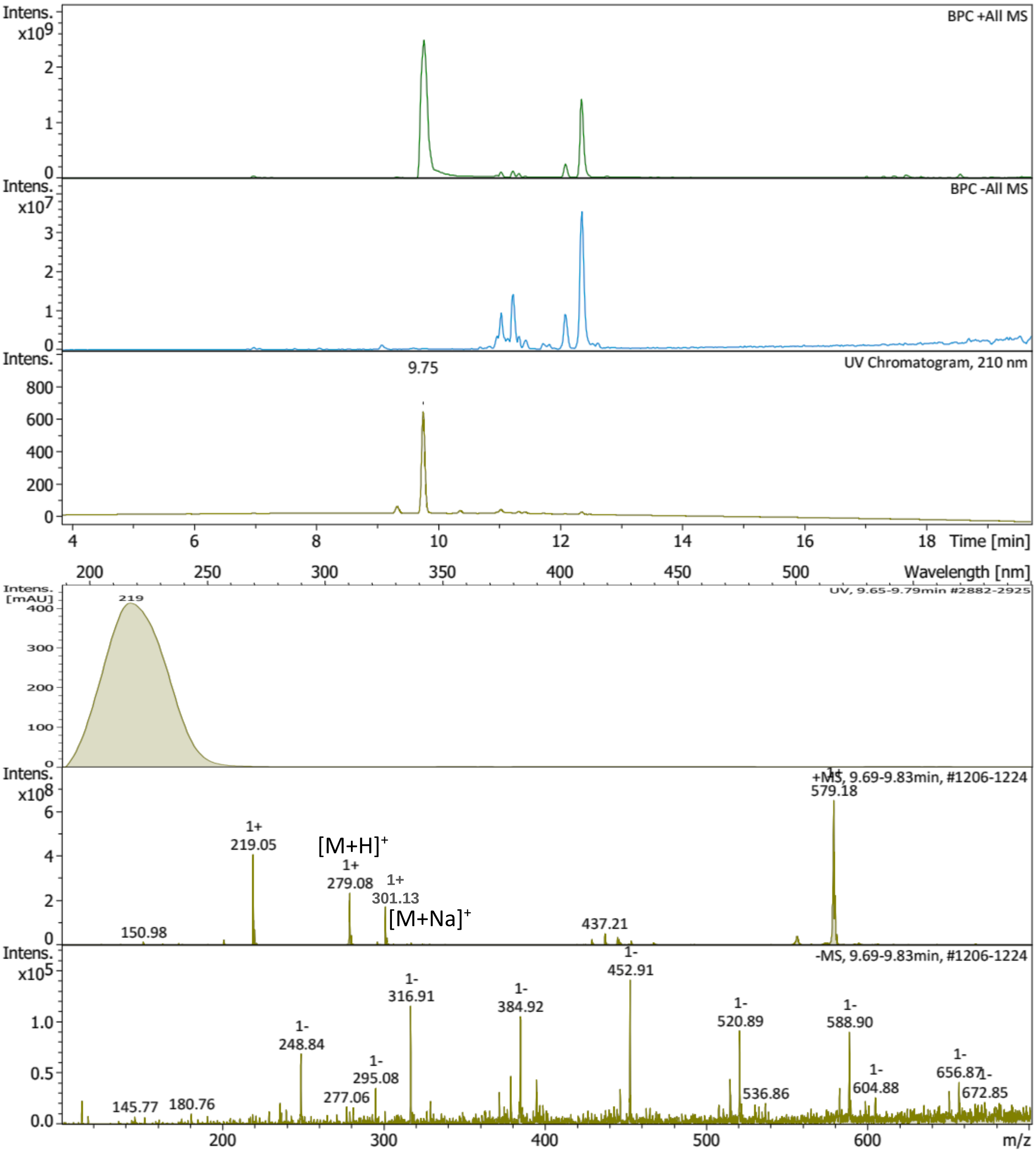

Figure S38. LR-ESI-MS of **5**.

Display Report

Analysis Info

		Acquisition Date	23.02.2022 13:40:10
Analysis Name	S:\DATA\MaXis\wsu20_Winnie Sum Chemutai\22_02\Dent Rice F15_P1-B-5_01_9612.d		
Method	pos_säure_10000_screening_ms_100_2500_line.m	Operator	ate06
Sample Name	Dent Rice F15	Instrument	maXis 255552.00037
Comment	Screening01<br>Waters Acquity UPLC BEH C_18 1,7um 2.1x50mm		

Acquisition Parameter

Source Type	ESI	Ion Polarity	Positive	Set Nebulizer	4.0 Bar
Focus	Not active	Set Capillary	4500 V	Set Dry Heater	200 °C
Scan Begin	50 m/z	Set End Plate Offset	-500 V	Set Dry Gas	10.0 l/min
Scan End	2500 m/z	Set Charging Voltage	0 V	Set Divert Valve	Waste
		Set Corona	0 nA	Set APCI Heater	0 °C

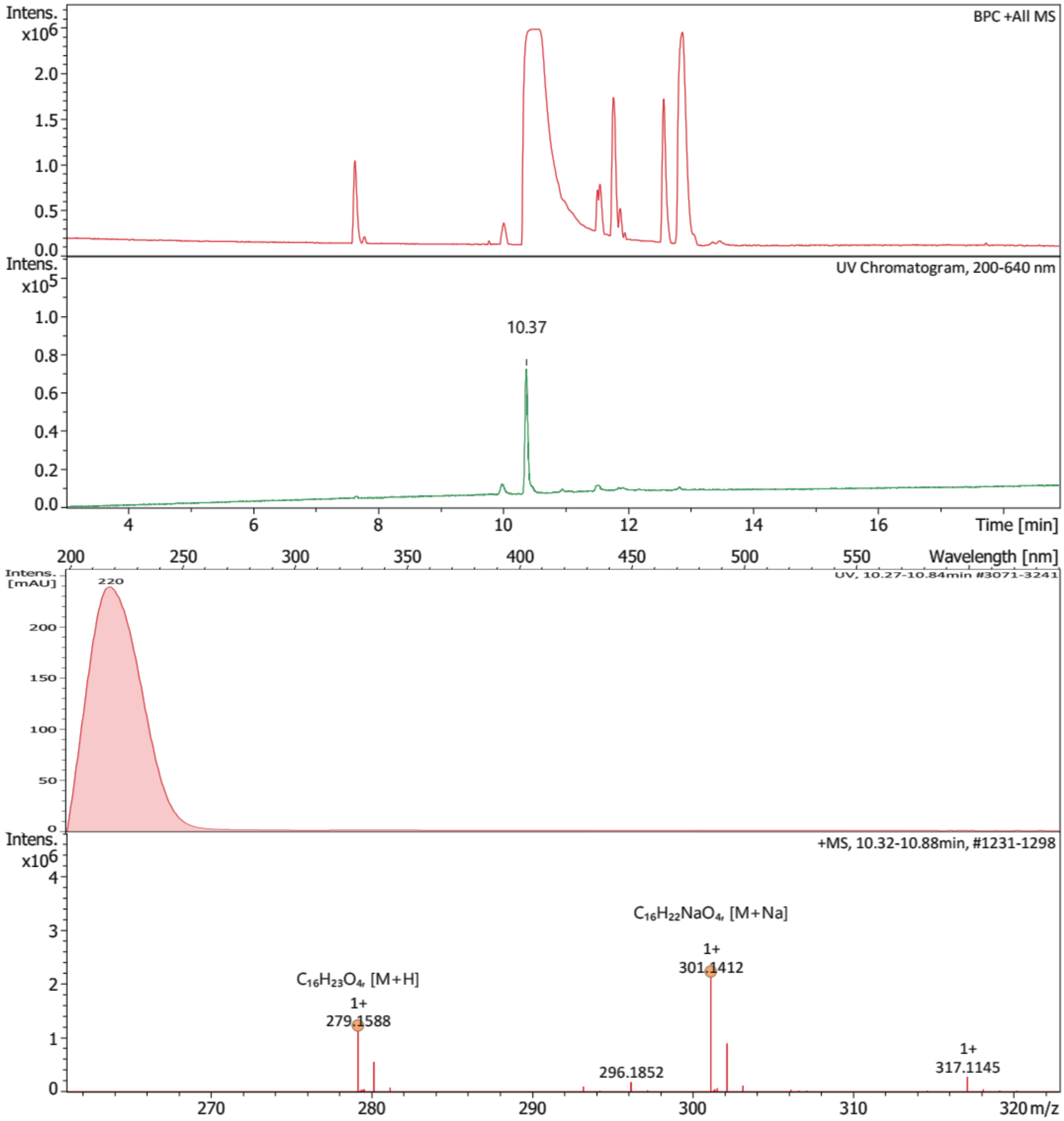

Dent Rice F15_P1-B-5_01_9612.d

Bruker Compass DataAnalysis 6.1	printed: 05.04.2024 16:10:17	by: sel22	Page 1 of 1

Figure S39. HR-ESI-MS of **5**.

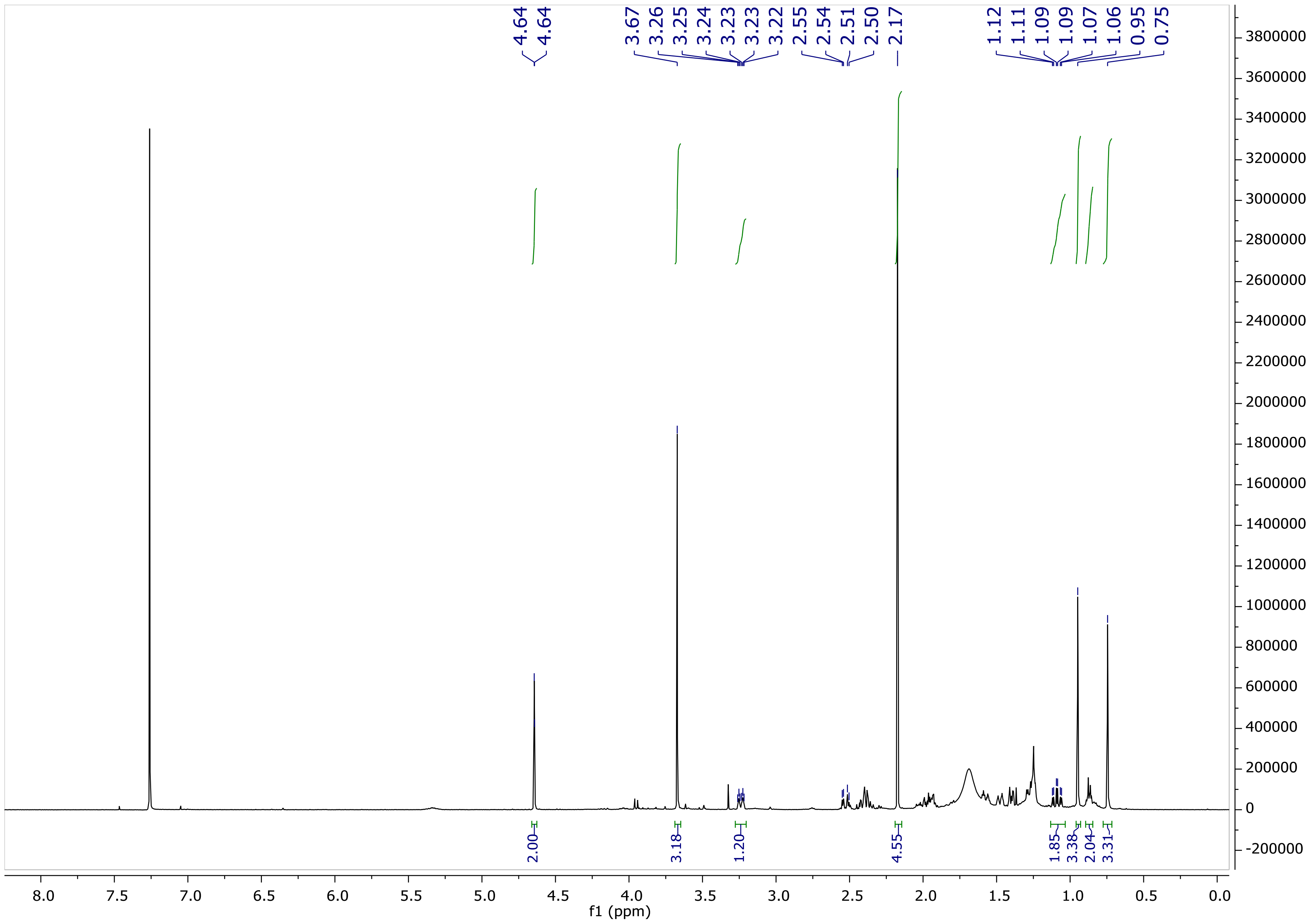

Figure S40. ^{1}H NMR spectrum of **5** in chloroform-*d* at 500 MHz.

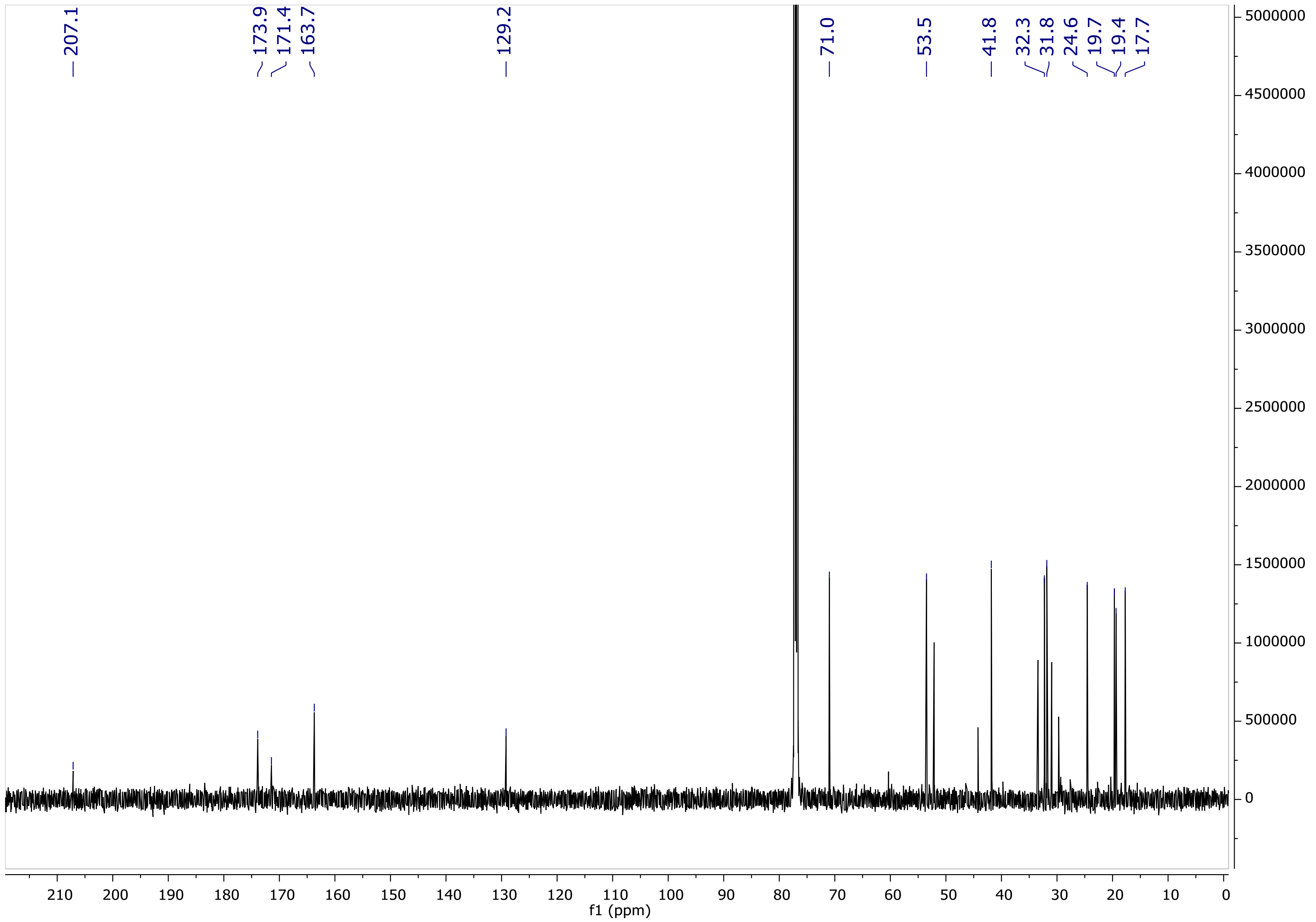

Figure S41. ^{13}C NMR spectrum of **5** in chloroform-*d* at 125 MHz.

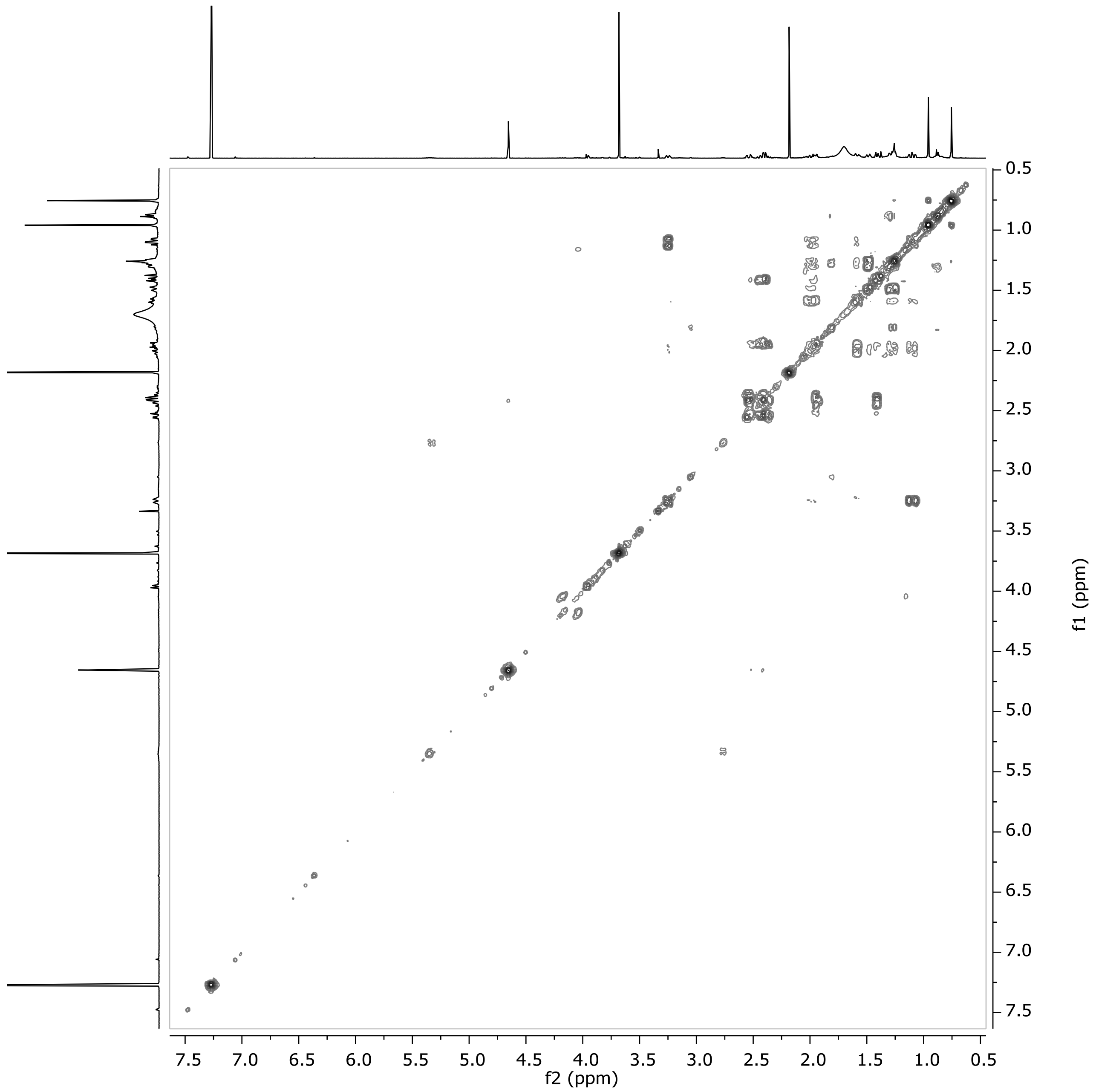

Figure S42. ^{1}H-^{1}H COSY spectrum of **5** in chloroform-*d* at 500 MHz.

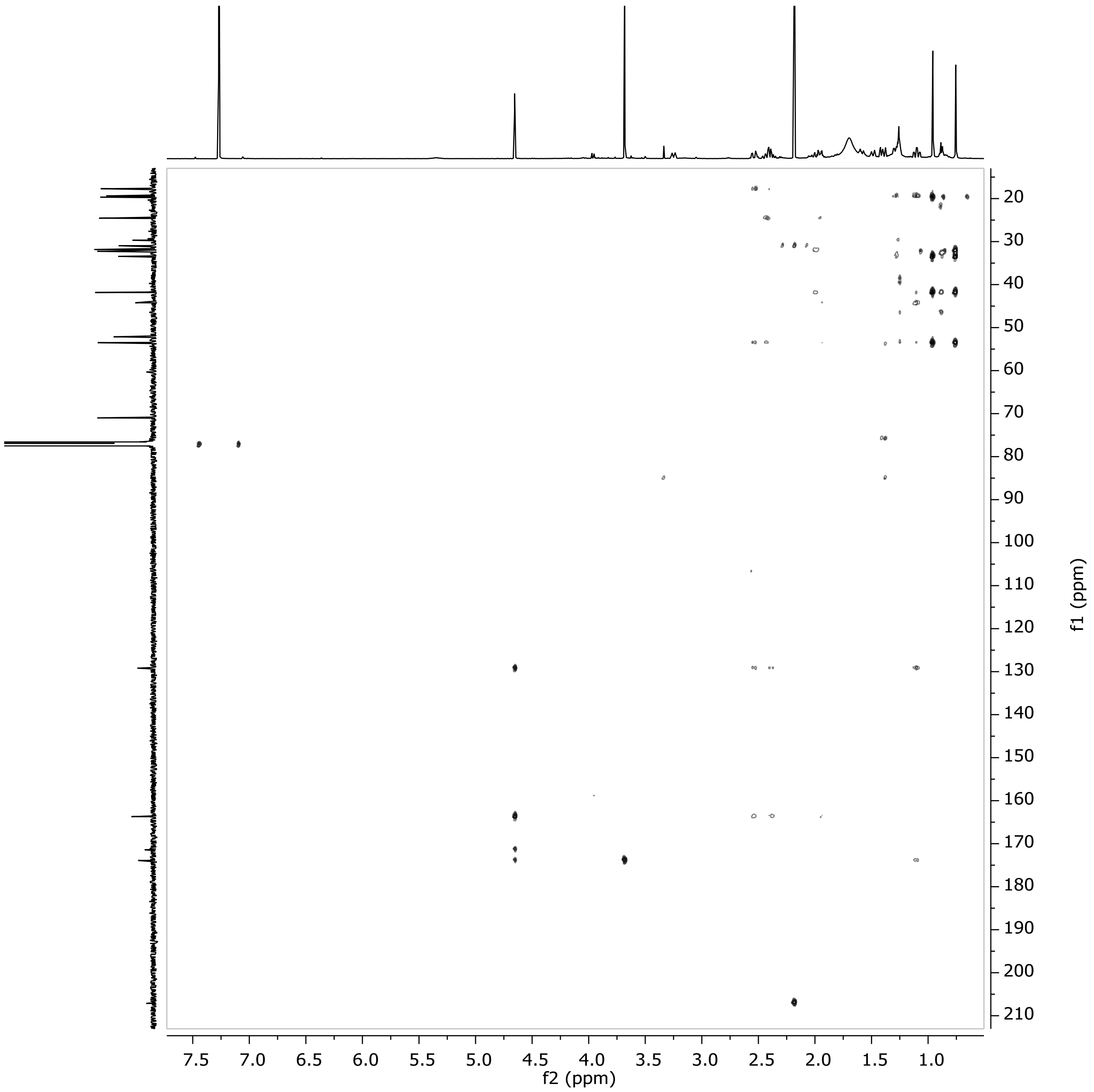

Figure S43. HMBC spectrum of **5** in chloroform-*d* at 500 MHz.

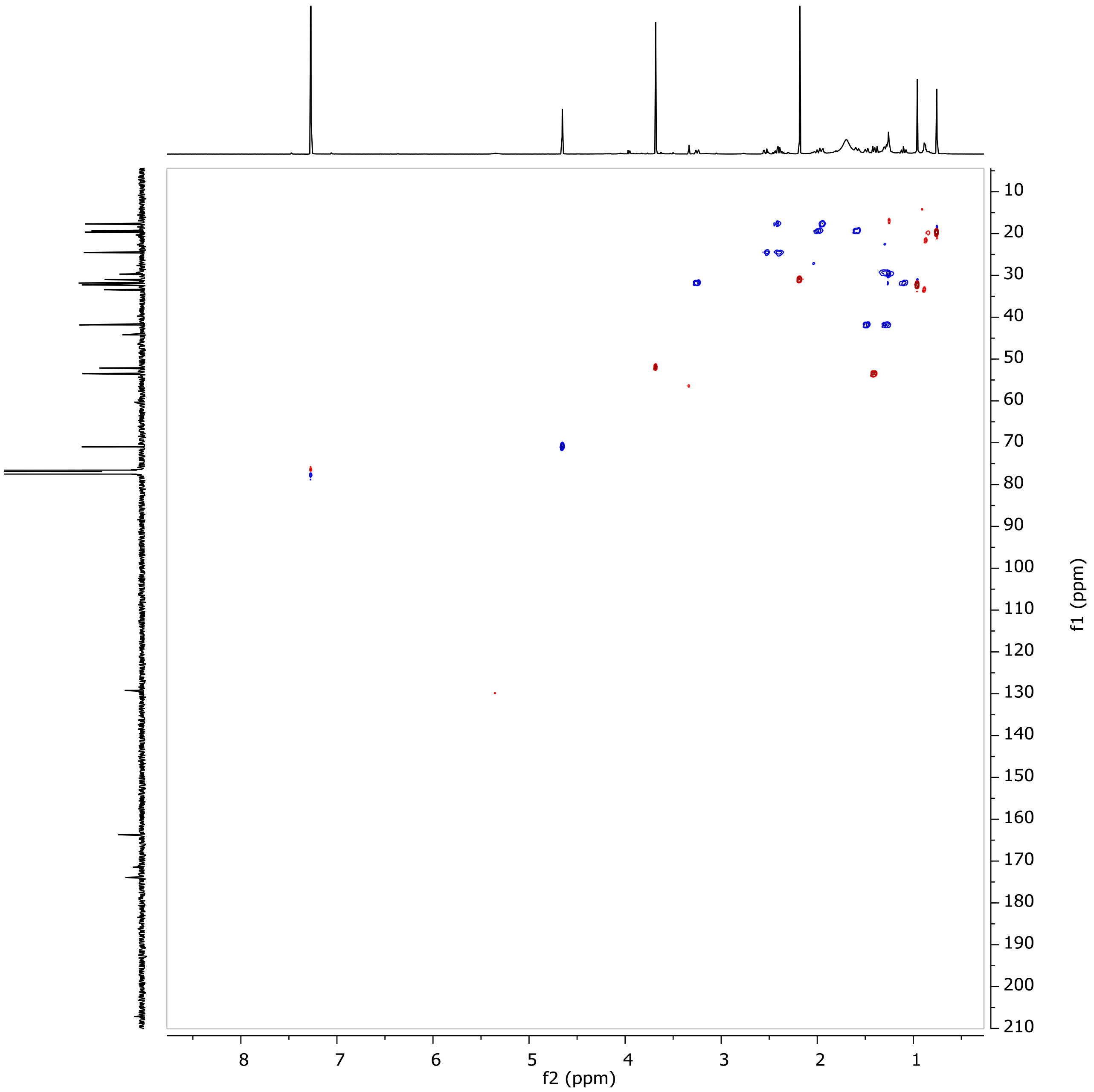

Figure S44. HSQC spectrum of **5** in chloroform-*d* at 500 MHz.

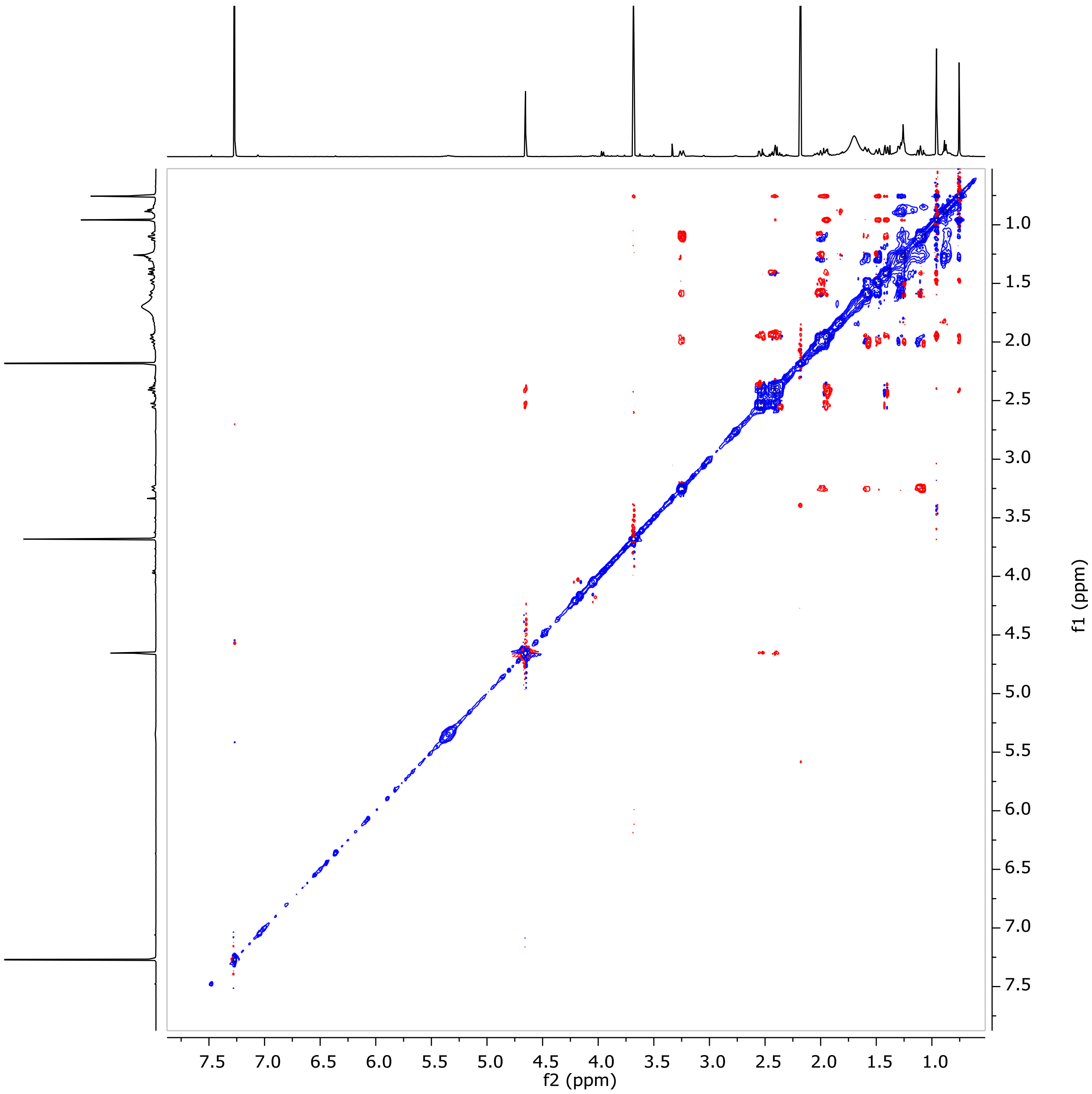

Figure S45. ROESY spectrum of **5** in chloroform-*d* at 500 MHz.
